# Supplementary material for: Molecularly engineered supramolecular fluorescent chemodosimeter for measuring epinephrine dynamics
Source: Nat Commun. 2025 Feb 21;16:1848. doi: 10.1038/s41467-025-57100-5 (PMC11845772; doi:10.1038/s41467-025-57100-5)
Supplement: Supplementary file 1 — Supplementary Information [file 41467_2025_57100_MOESM1_ESM.pdf]

Supplementary Information

Molecularly engineered supramolecular fluorescent chemodosimeter for measuring epinephrine dynamics

Yudan Zhao, Yuxiao Mei*, Zhichao Liu, Jing Sun, Yang Tian*

Shanghai Key Laboratory of Green Chemistry and Chemical Processes, School of Chemistry and Molecular Engineering, East China Normal University, Dongchuan Road 500, Shanghai 200241, P.R. China

E-mail: yxmei@chem.ecnu.edu.cn.

ytian@chem.ecnu.edu.cn.

Contents

1. Synthesis and characterization of G1-G3
2. Optical properties of G1-G3 molecule, MB molecule assembled with different size cavities
3. Selectivity, competition tests and response speed of different probes toward determination of EP
4. The theoretical calculation proving high selectivity and fast response speed of CMG2
5. Optical properties of CMG2 probe in response to EP
6. Sensing mechanism of Host-guest self-assembly of CMG2 and CMG2+EP
7. FACS, MTT and photostability measurements of CMG2 probe in live cells
8. Fluorescence imaging and simultaneous quantification EP in vivo
9. The optical properties characterization, co-localization and biocompatibility of the optical fiber array

1. Synthesis and characterization of G1, G2 and G3

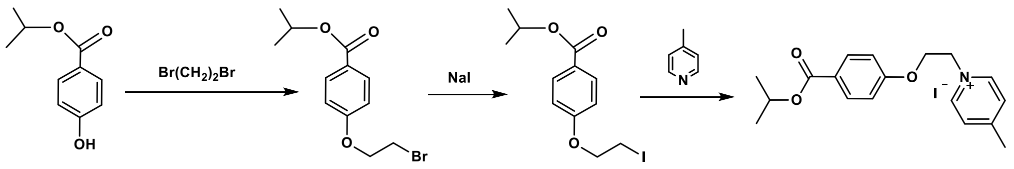

(1) (4) (7)

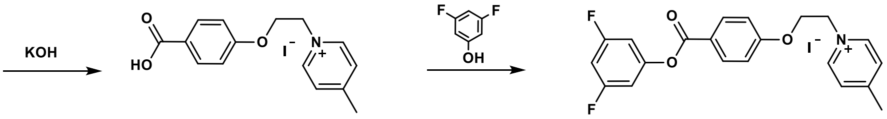

(10) (13)

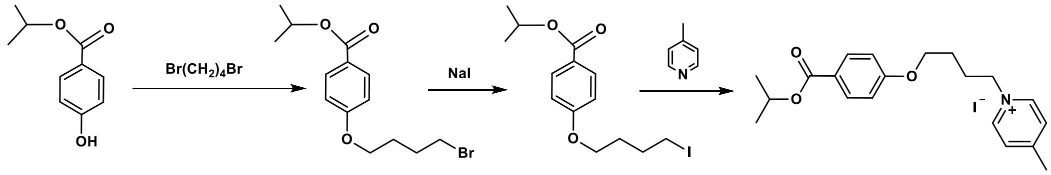

(2) (5) (8)

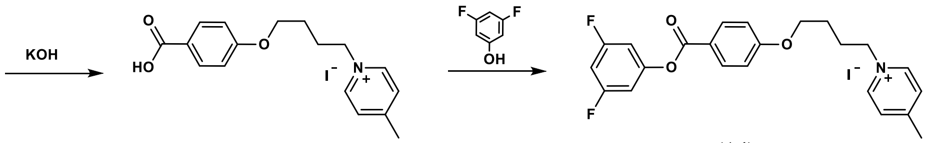

(11) (14)

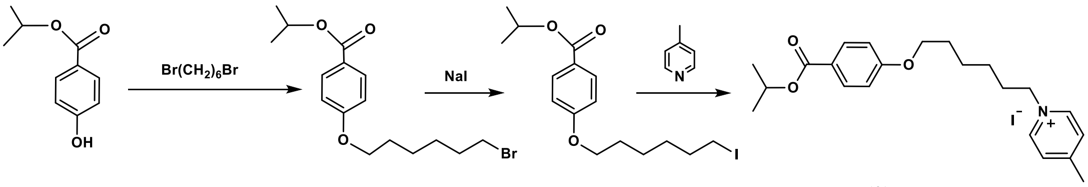

(3) (6) (9)

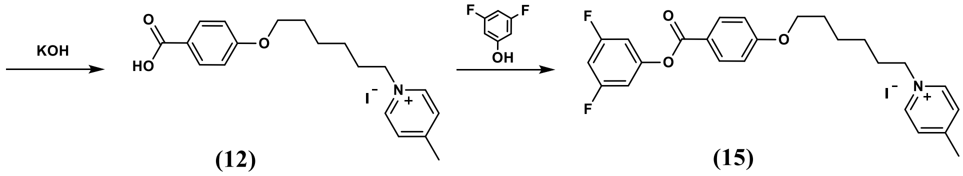

(12) (15)

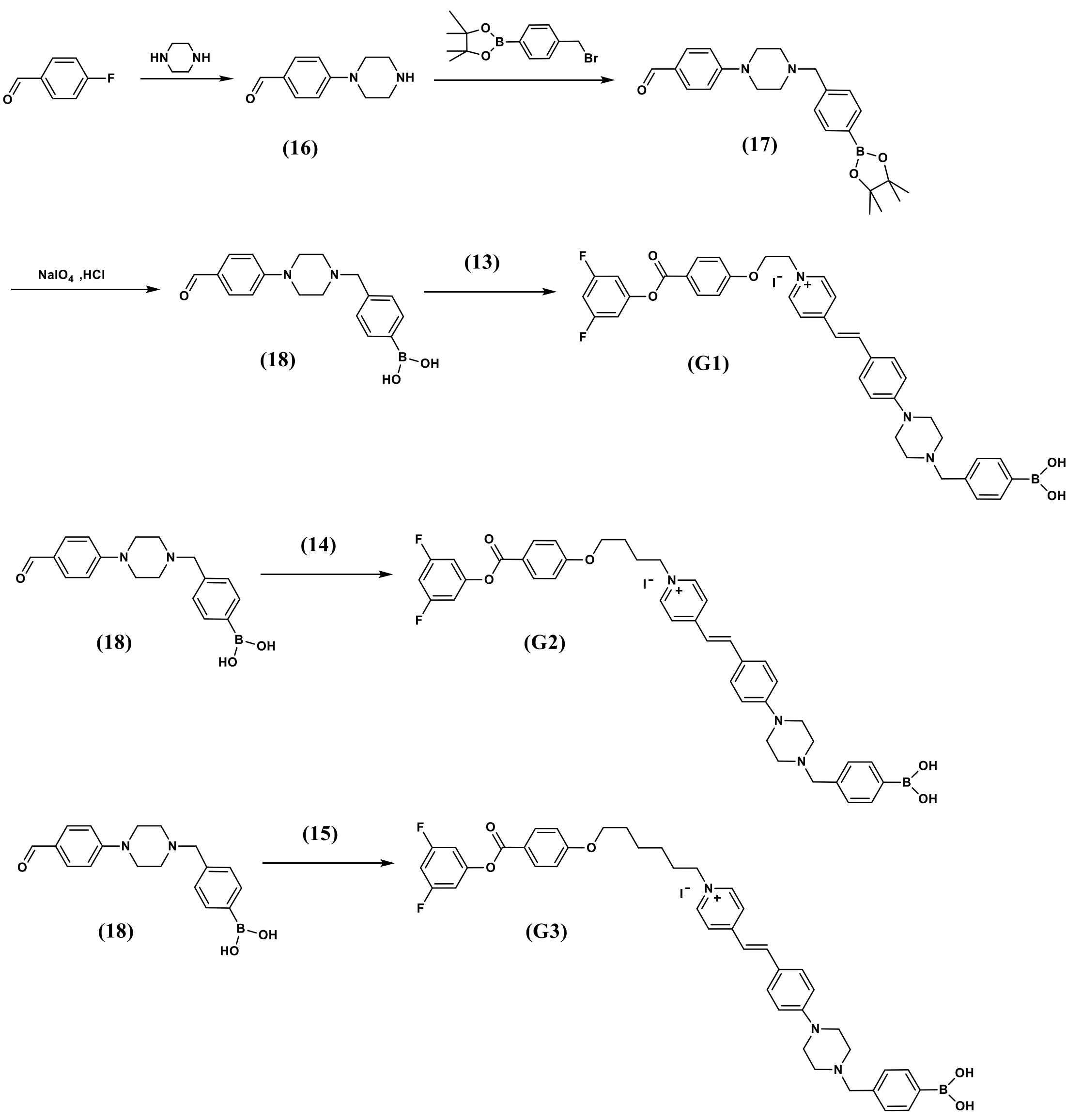

Supplementary Fig. 1 The synthesis procedures of guest molecules G1-G3.

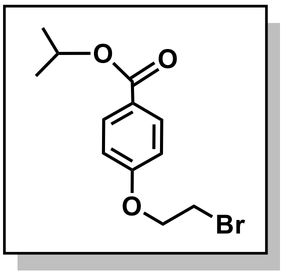

Synthesis of Compound 1: A mixture of isopropyl paraben (1.8 g, 10.0 mmol), 1,2-dibromoethane (2.8 g, 15.0 mmol), and K_2CO_3 (4.0 g, 30.0 mmol) in acetonitrile (200 mL) were stirred at 100 °C overnight under a nitrogen atmosphere. After being cooled to room temperature, the resulting mixture was evaporated to dryness. The crude product was purified by silica gel column chromatography using PE/EA (50:1, v/v) as eluent to get Compound 1 as a white solid (2.27 g, 79%). ^{1}H NMR (500 MHz, 298 K, Chloroform-*d*) δ 8.01-7.98 (m, 2H), 6.93-6.90 (m, 2H), 5.27-5.22 (m, 1H), 4.37-4.35 (m, 2H), 3.69-3.66 (m, 2H), 1.38-1.37 (d, *J* = 6.5 Hz, 6H). ^{13}C NMR (125 MHz, 298 K, Chloroform-*d*) δ

165.75, 161.65, 131.63, 124.13, 114.15, 68.10, 67.85, 28.68, 22.03. HR-MS (ESI): m/z calcd for $C_{12}H_{15}BrNaO_3$ $[M+Na]^+$: 309.0097; found: 309.0085.

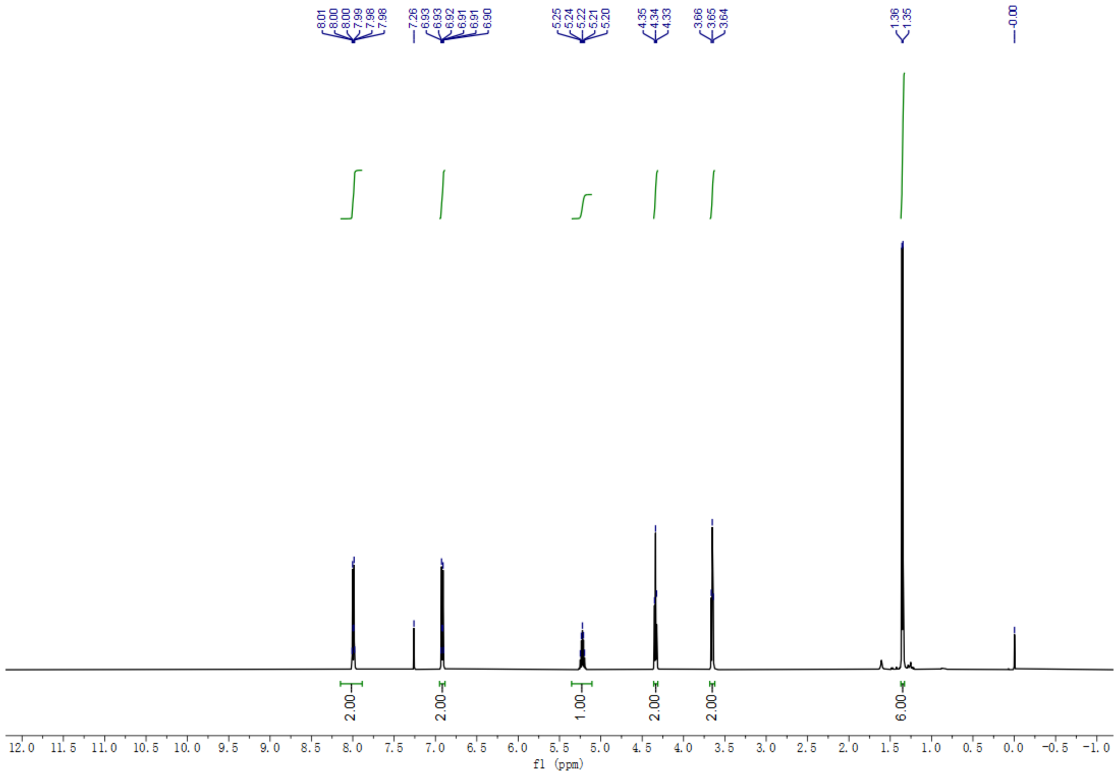

Supplementary Fig. 2 ^{1}H NMR spectrum of compound 1.

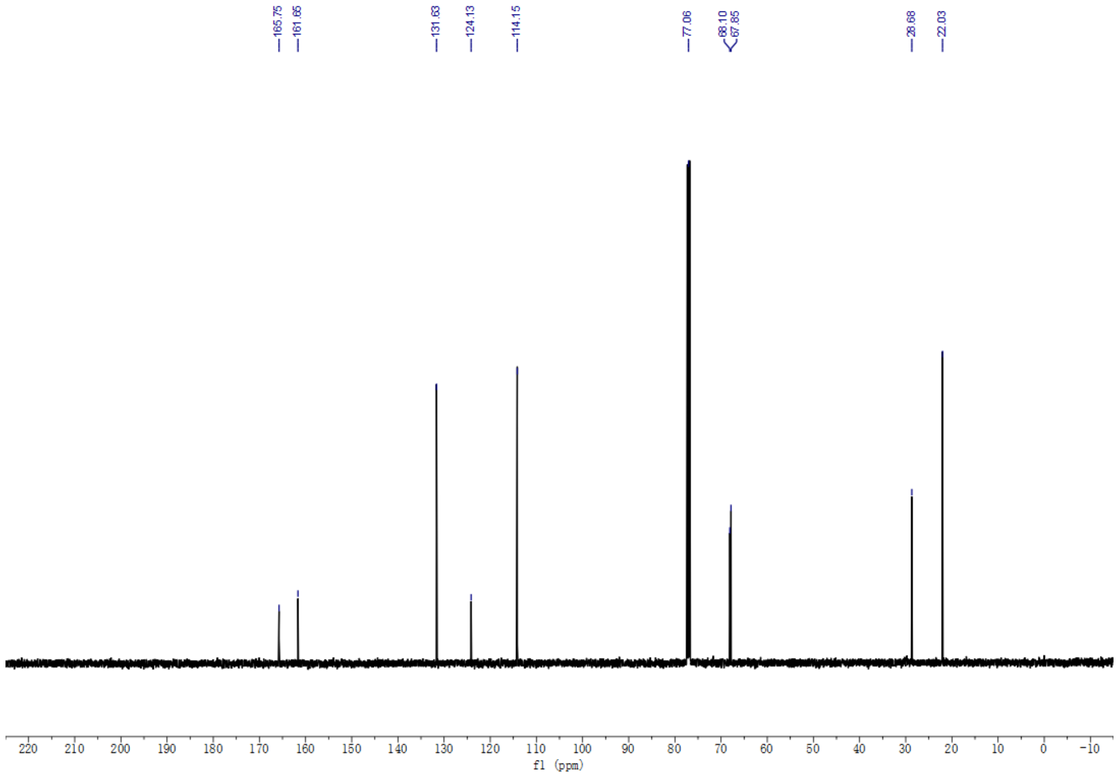

Supplementary Fig. 3 ^{13}C NMR spectrum of compound 1.

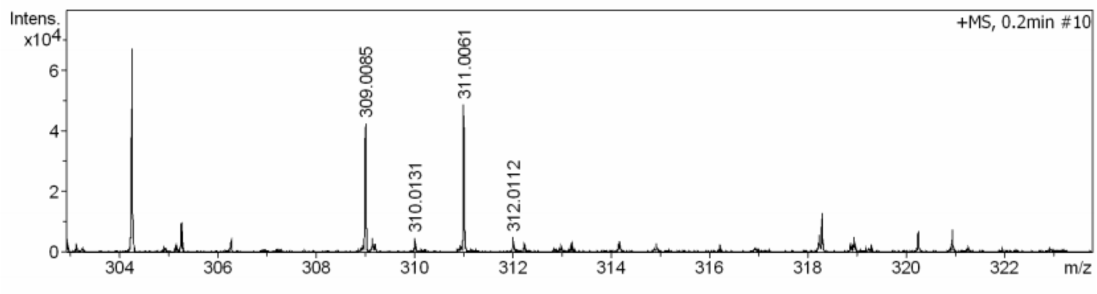

#	m/z	Res.	S/N	I	I %	FWHM
1	309.0085	17611	89.8	42380	87.5	0.0175
2	310.0131	17884	9.9	4660	9.6	0.0173
3	311.0061	21023	102.6	48448	100.0	0.0148
4	312.0112	13407	10.2	4832	10.0	0.0233

Meas. m/z	#	Ion Formula	m/z	err [ppm]	mSigma	#	Score	rdb	e⁻ Conf	N-Rule
309.0085	1	C12H15BrNaO3	309.0097	3.7	60.5	2	100.00	4.5	even	ok

Supplementary Fig. 4 HR-ESI-MS spectrum of compound 1.

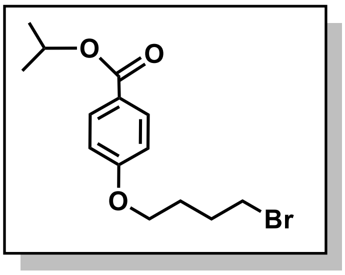

Synthesis of Compound 2: The synthesis of compound 2 was the same as compound 1 (2.62 g, 83%). ^{1}H NMR (500 MHz, 298 K, Chloroform-*d*) δ 7.99-7.96 (m, 2H), 6.90-6.87 (m, 2H), 5.24-5.19 (m, 1H), 4.06-4.03 (m, 2H), 3.50-3.47 (m, 2H), 2.10-2.04 (m, 2H), 1.99-1.95 (m, 2H), 1.35-1.34 (d, J = 6.0 Hz, 6H).^{13}C NMR (125 MHz, 298 K, Chloroform-*d*) δ 165.88, 162.48, 131.53, 123.45, 113.95, 67.98, 67.05, 33.32, 29.40, 27.80, 22.04. HR-MS (ESI): m/z calcd for $C_{14}H_{19}BrNaO_3$ $[M+Na]^+$: 337.0410; found: 337.0406.

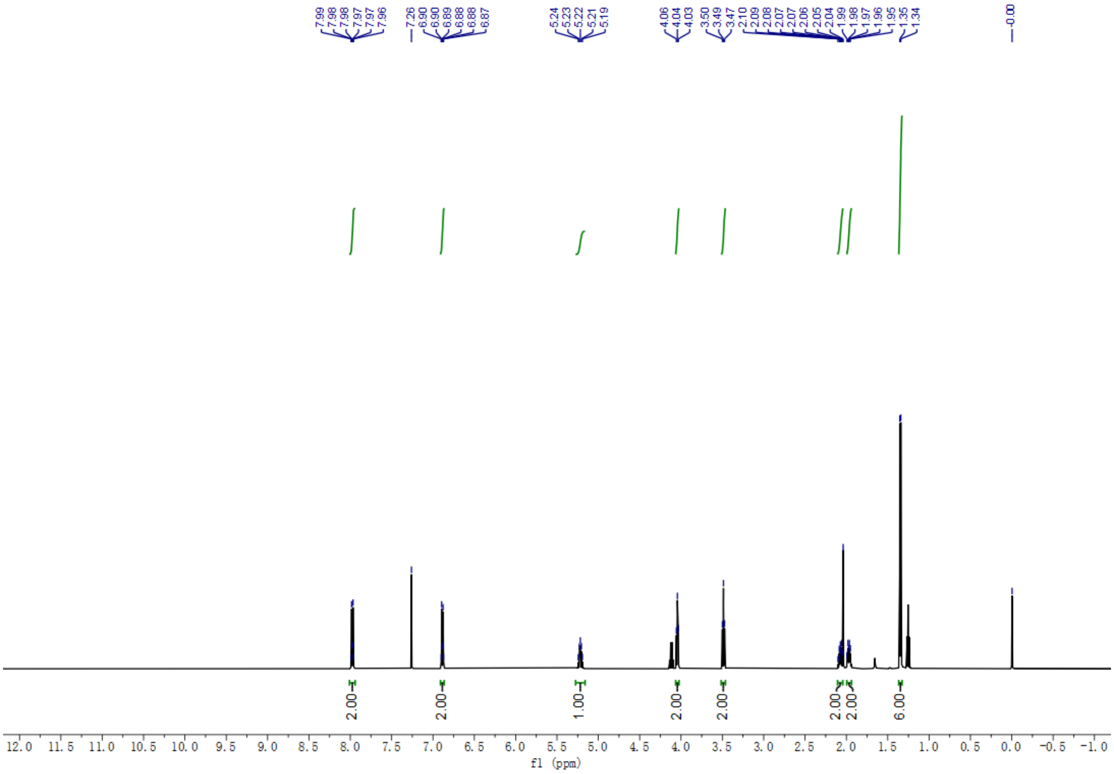

Supplementary Fig. 5 ^{1}H NMR spectrum of compound 2.

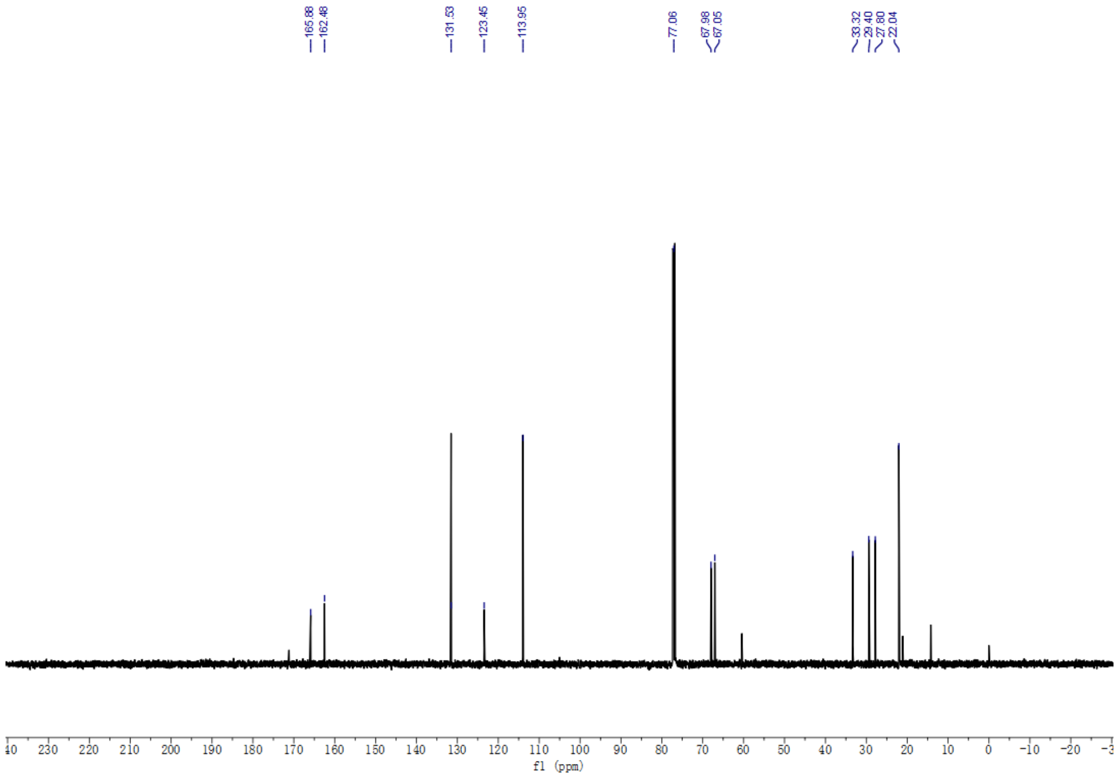

Supplementary Fig. 6 ^{13}C NMR spectrum of compound 2.

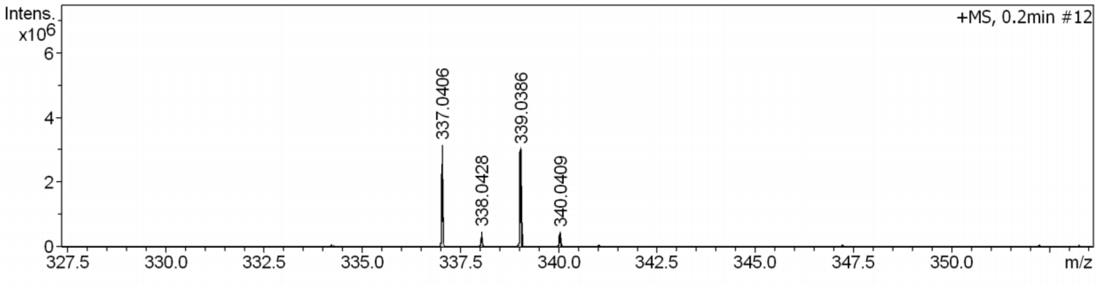

#	m/z	Res.	S/N	I	I %	FWHM
1	337.0406	14267	2596.1	3142028	100.0	0.0236
2	338.0428	18807	375.7	455232	14.5	0.0180
3	339.0386	14139	2478.3	3007616	95.7	0.0240
4	340.0409	18229	359.1	436424	13.9	0.0187

Meas. m/z	#	Ion Formula	m/z	err [ppm]	mSigma	#	Score	rdb	e⁻ Conf	N-Rule
337.0406	1	C14H19BrNaO3	337.0410	1.1	16.4	1	100.00	4.5	even	ok

Supplementary Fig. 7 HR-ESI-MS spectrum of compound 2.

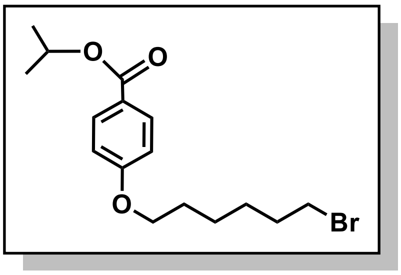

Synthesis of Compound 3: The synthesis of compound 3 was the same as compound 1 (2.82 g, 82%). ^{1}H NMR (500 MHz, 298 K, Chloroform-*d*) δ 7.98-7.96 (m, 2H), 6.90-6.87 (m, 2H), 5.24-5.19 (m, 1H), 4.01-3.99 (m, 2H), 3.43-3.40 (m, 2H), 1.92-1.86 (m, 2H), 1.84-1.78 (m, 2H), 1.52-1.49 (m, 4H), 1.35-1.34 (d, *J* = 6.0 Hz, 6H). ^{13}C NMR (125 MHz, 298

K, Chloroform-*d*) δ 165.90, 162.68, 131.48, 123.21, 113.94, 67.89, 33.74, 32.64, 28.95, 27.89, 25.24, 22.01. HR-MS (ESI): m/z calcd for $C_{16}H_{23}BrNaO_3$ [M+Na]$^+$: 365.0723; found: 363.0711.

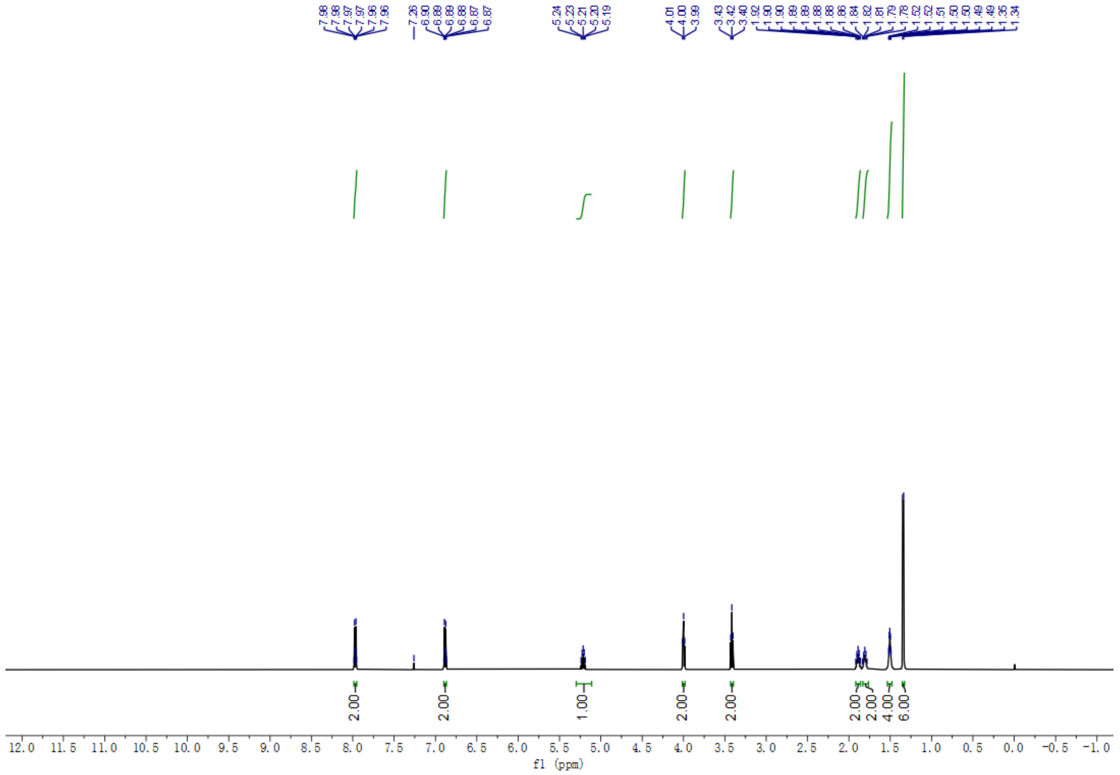

Supplementary Fig. 8 ^{1}H NMR spectrum of compound 3.

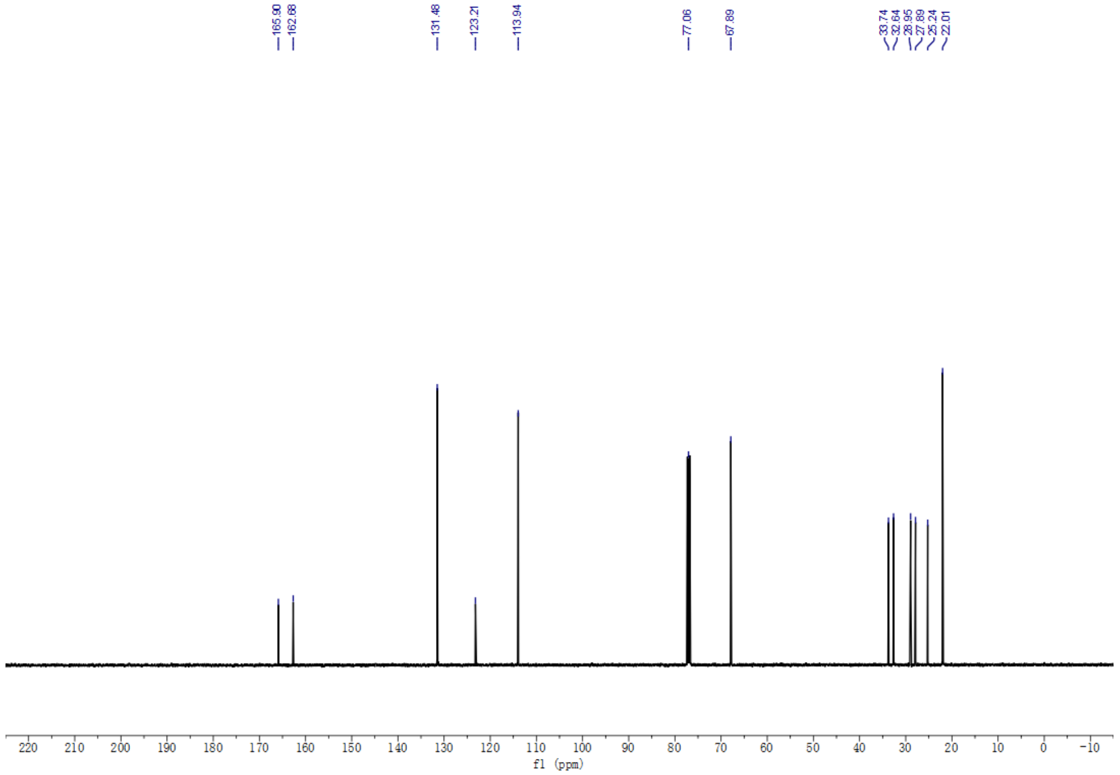

Supplementary Fig. 9 ^{13}C NMR spectrum of compound 3.

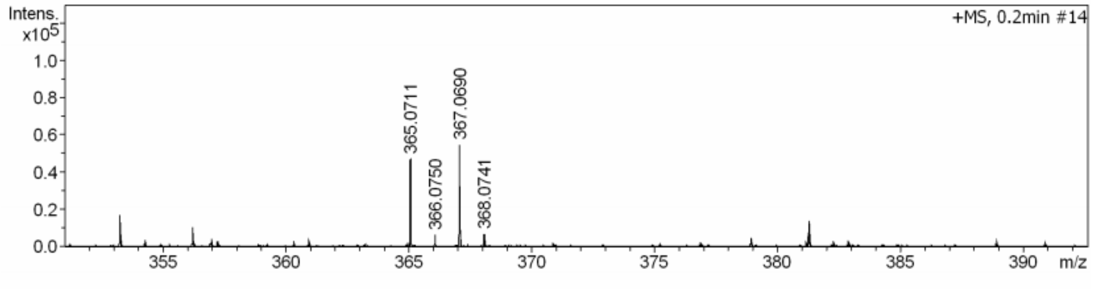

#	m/z	Res.	S/N	I	I %	FWHM
1	365.0711	20151	112.3	46904	86.0	0.0181
2	366.0750	15890	14.1	5872	10.8	0.0230
3	367.0690	21451	130.7	54536	100.0	0.0171
4	368.0741	18984	15.4	6440	11.8	0.0194

Meas. m/z	#	Ion Formula	m/z	err [ppm]	mSigma		Score	rdb	e⁻ Conf	N-Rule
365.0711	1	C16H23BrNaO3	365.0723	3.2	74.3	4	98.15	4.5	even	ok

Supplementary Fig. 10 HR-ESI-MS spectrum of compound 3.

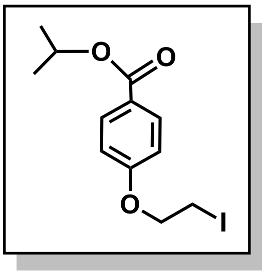

Synthesis of Compound 4: Compound 1 (0.858 g, 3.0 mmol) and NaI (1.8 g, 12 mmol) were dissolved in tetrahydrofuran (50 mL). The reaction mixture was stirred and refluxed for 12 h under a nitrogen atmosphere, the resulting mixture was evaporated to dryness. The solvent was evaporated under reduced pressure to get Compound 4 as white solid (952 mg, 95%). ^{1}H NMR (500 MHz, 298 K, DMSO-d_6) δ 7.90-7.88 (dd, J_1 = 2.0 Hz, J_2 = 6.5 Hz, 2H), 7.06-7.04 (dd, J_1 = 2.0 Hz, J_2 = 6.5 Hz, 2H), 5.12-5.07 (m, 1H), 4.34-4.32 (m, 2H), 3.55-3.52 (m, 2H), 1.30-1.29 (d, J = 2.5 Hz, 6H).^{13}C NMR (125 MHz, 298 K, DMSO-d_6) δ 164.82, 161.55, 131.19, 122.89, 114.59, 68.42, 67.68, 21.72, 3.36. HR-MS (ESI): m/z calcd for $C_{12}H_{15}INaO_3$ $[M+Na]^+$: 356.9958; found: 356.9962.

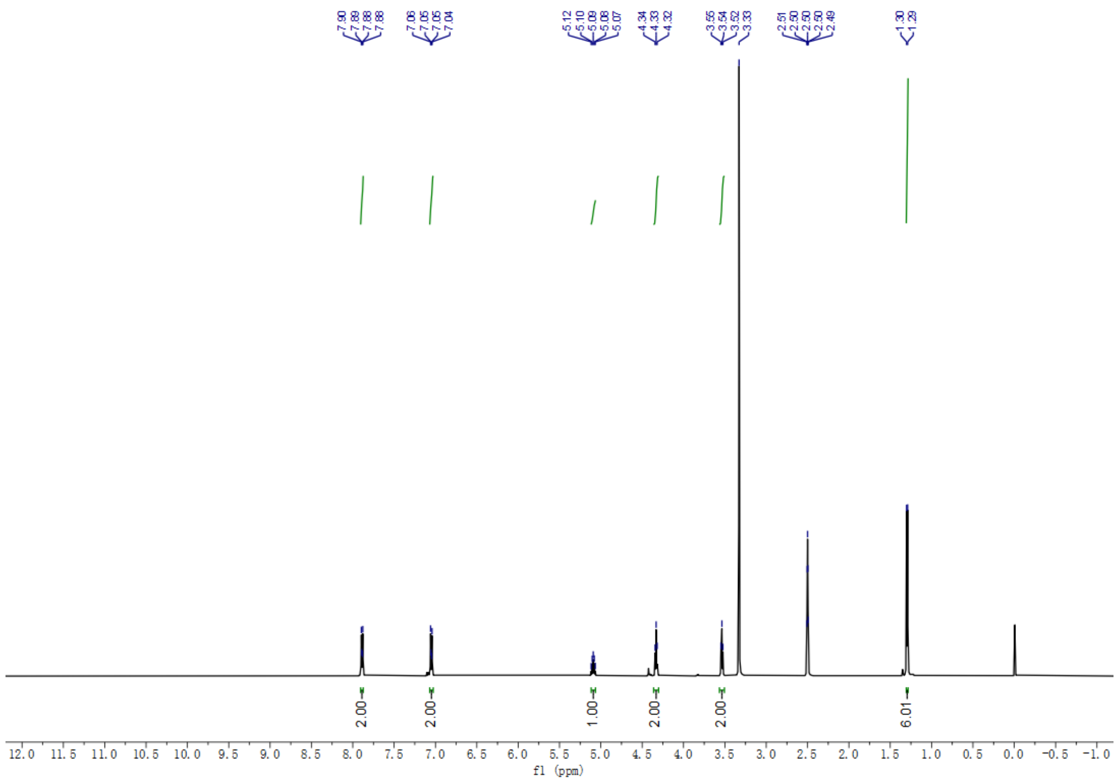

Supplementary Fig. 11 ^{1}H NMR spectrum of compound 4.

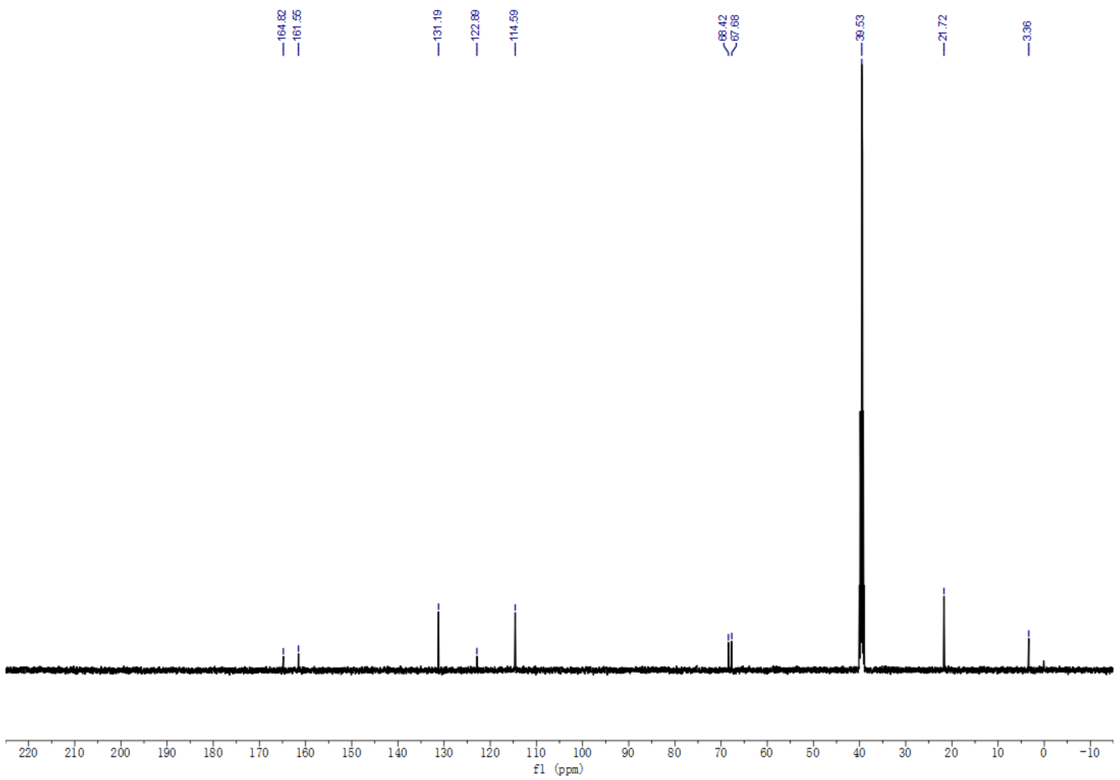

Supplementary Fig. 12 ^{13}C NMR spectrum of compound 4.

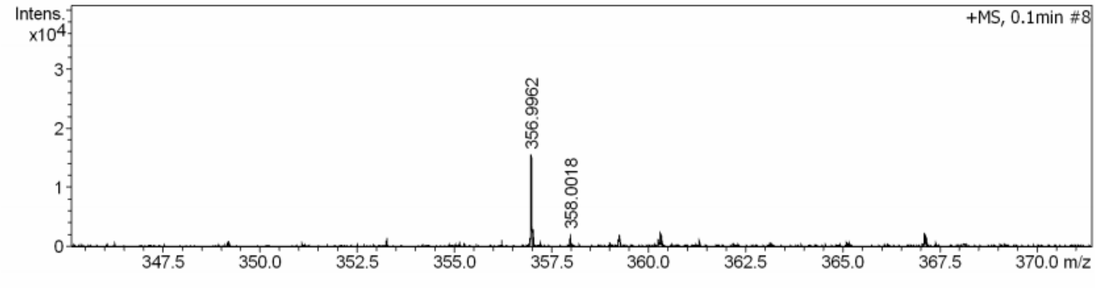

#	m/z	Res.	S/N	I	I %	FWHM
1	356.9962	17940	40.7	15492	100.0	0.0199
2	358.0018	24826	5.4	2064	13.3	0.0144

Meas. m/z	#	Ion Formula	m/z	err [ppm]	mSigma	#	Score	rdb	e⁻ Conf	N-Rule
356.9962	1	C12H15INaO3	356.9958	-1.0	8.2	1	100.00	4.5	even	ok

Supplementary Fig. 13 HR-ESI-MS spectrum of compound 4.

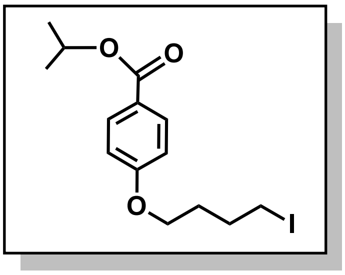

Synthesis of Compound 5: The synthesis of compound 4 was the same as compound 4 (1.03 g, 95%). ^{1}H NMR (500 MHz, 298 K, Chloroform-*d*) δ 7.99-7.97 (m, 2H), 6.90-6.87 (m, 2H), 5.24-5.20 (m, 1H), 4.05-4.03 (m, 2H), 3.27-3.25 (m, 2H), 2.06-2.01 (m, 2H), 1.95-1.90 (m, 2H), 1.36-1.35 (d, *J* = 5.5 Hz, 6H).^{13}C NMR (125 MHz, 298 K, Chloroform-*d*) δ 165.90, 162.49, 131.55, 123.45, 113.96, 67.98, 66.85, 30.05, 30.05, 22.05, 6.23. HR-MS (ESI): m/z calcd for $C_{14}H_{19}BrIO_3$ $[M+Na]^+$: 385.0271; found: 385.0261.

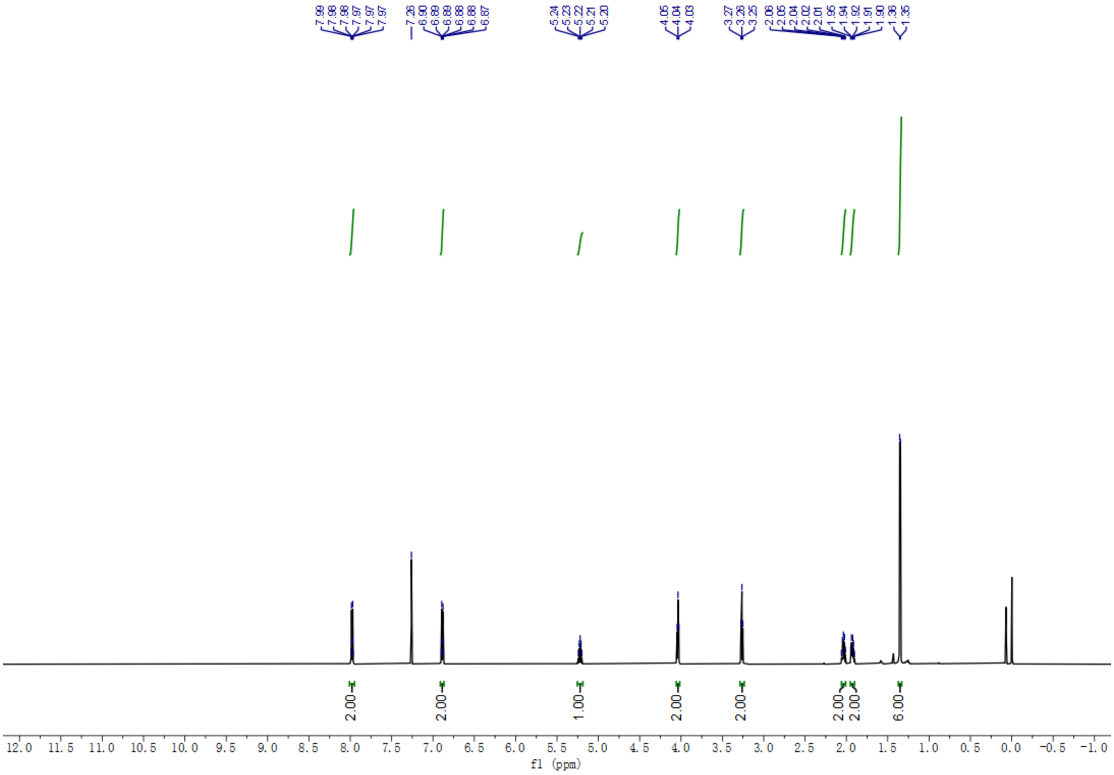

Supplementary Fig. 14 ^{1}H NMR spectrum of compound 5.

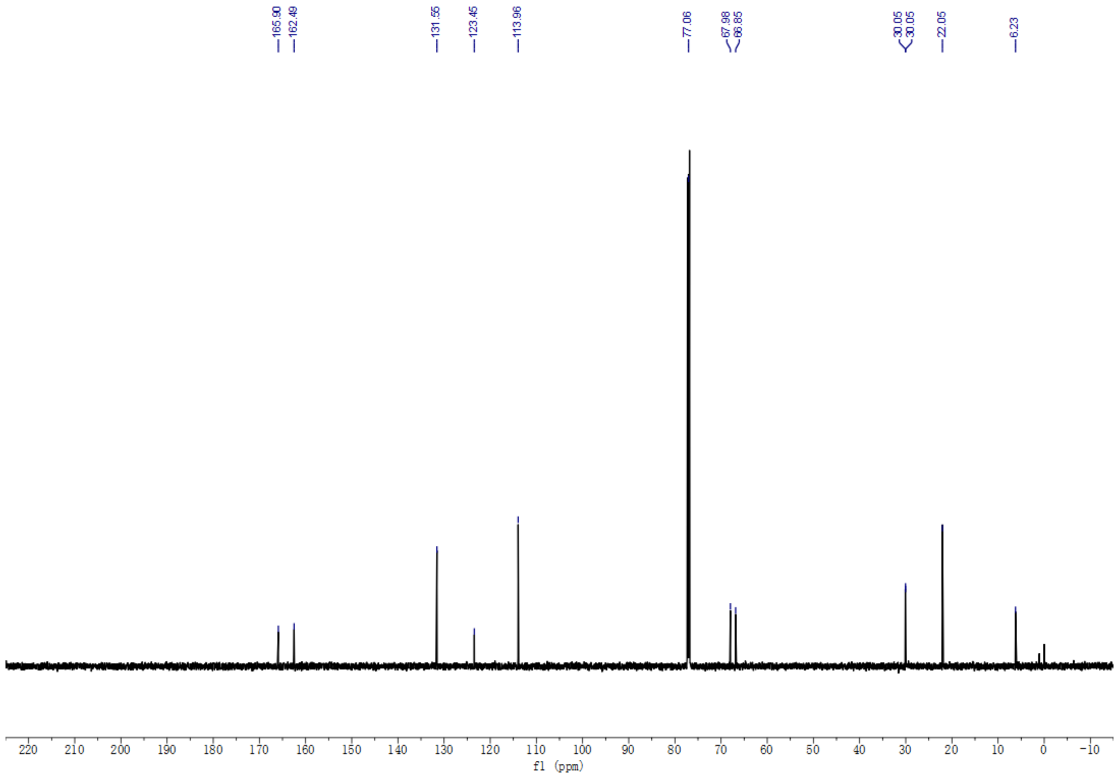

Supplementary Fig. 15 ^{13}C NMR spectrum of compound 5.

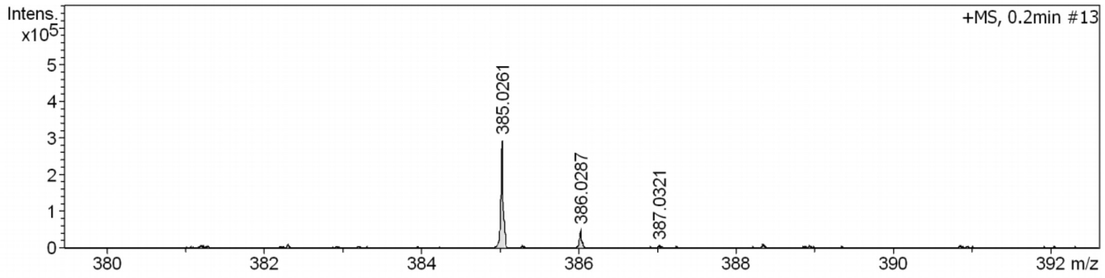

#	m/z	Res.	S/N	I	I %	FWHM
1	385.0261	16357	230.7	293600	100.0	0.0235
2	386.0287	16171	40.3	51288	17.5	0.0239
3	387.0321	14185	5.3	6736	2.3	0.0273

Meas. m/z	#	Ion Formula	m/z	err [ppm]	mSigma		Score	rdb	e⁻ Conf	N-Rule
385.0261	1	C14H19INaO3	385.0271	2.6	12.0	1	100.00	4.5	even	ok

Supplementary Fig. 16 HR-ESI-MS spectrum of compound 5.

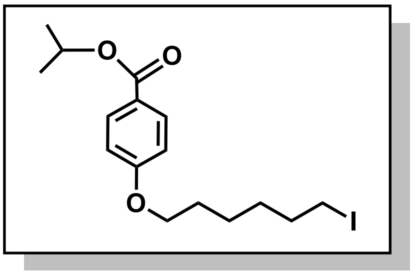

Synthesis of Compound 6: The synthesis of compound 6 was the same as compound 1 (1.11 g, 95%). ^{1}H NMR (500 MHz, 298 K, DMSO-*d*$_6$) δ 7.88-7.85 (m, 2H), 7.02-6.99 (m, 2H), 5.11-5.06 (m, 1H), 4.03-4.01 (m, 2H), 3.28-3.26 (m, 2H), 1.80-1.69 (m, 4H), 1.44-1.38 (m, 4H), 1.29-1.28 (d, *J* = 6.5 Hz, 6H). ^{13}C NMR (125 MHz, 298 K, DMSO-*d*$_6$) δ 164.91, 162.46, 131.13,

122.30, 114.3, 67.73, 67.57, 32.80, 29.59, 28.33, 24.37, 21.74, 8.95. HR-MS (ESI): m/z calcd for $C_{16}H_{23}INaO_3$ $[M+Na]^+$: 413.0584; found: 413.0589.

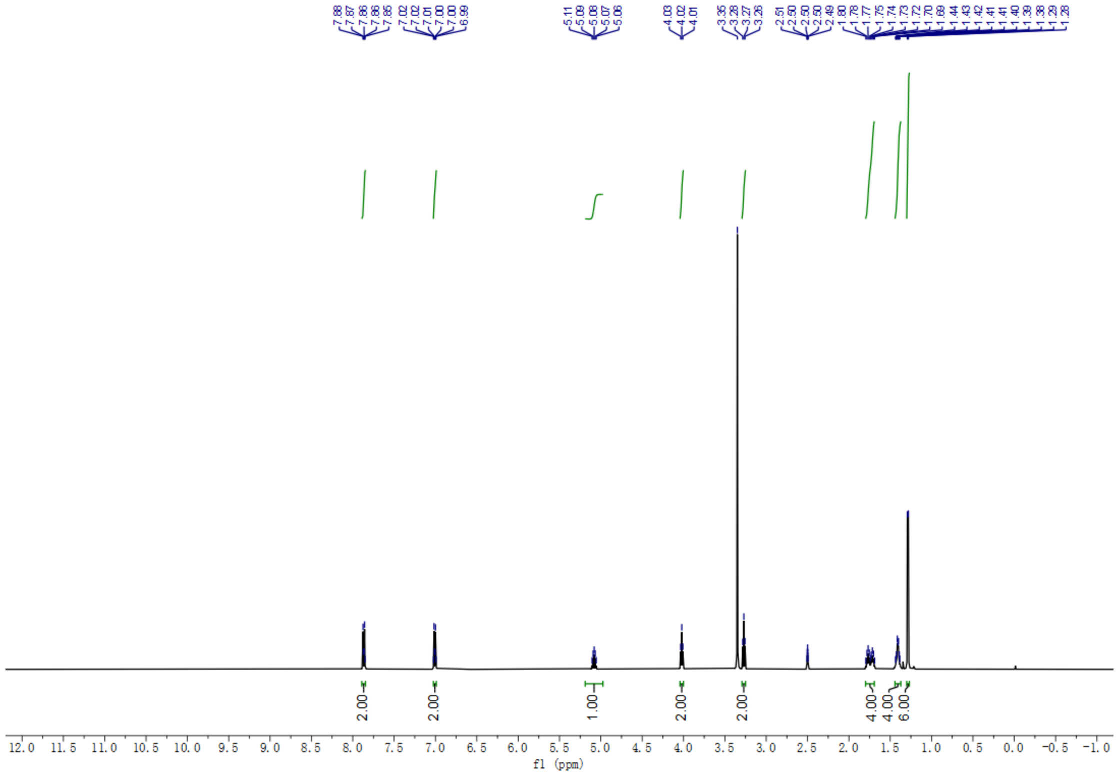

Supplementary Fig. 17 ^1H NMR spectrum of compound 6.

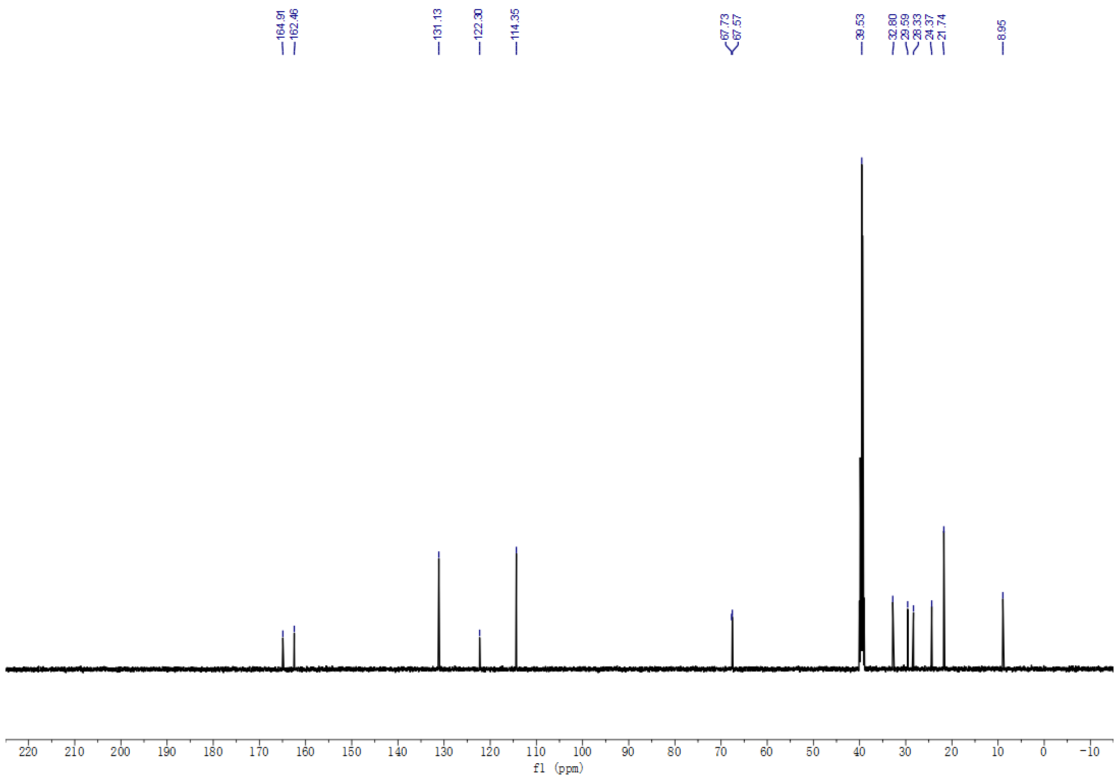

Supplementary Fig. 18 ^{13}C NMR spectrum of compound 6.

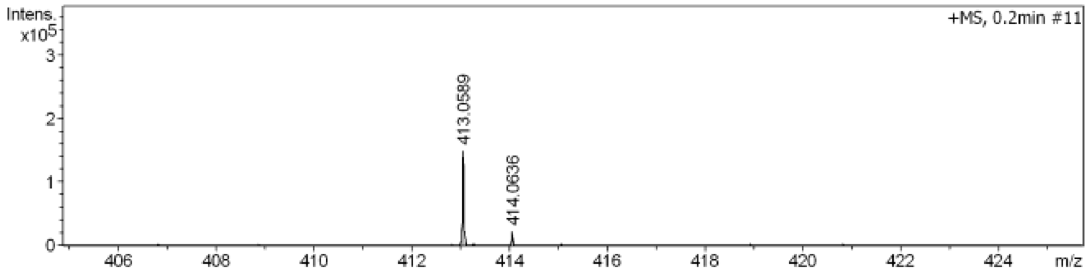

#	m/z	Res.	S/N	I	I %	FWHM
1	413.0589	21407	346.2	149028	100.0	0.0193
2	414.0636	22454	51.2	22044	14.8	0.0184
3	415.0680	15425	6.0	2600	1.7	0.0269

Meas. m/z	#	Ion Formula	m/z	err [ppm]	mSigma	#	Score	rdb	e⁻ Conf	N-Rule
413.0589	1	C16H23INaO3	413.0584	-1.3	16.8	1	100.00	4.5	even	ok

Supplementary Fig. 19 HR-ESI-MS spectrum of compound 6.

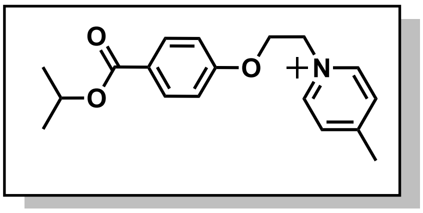

Synthesis of Compound 7: Compound 4 (1.0 g, 3.0 mmol) and 4-methylpyridine (308 mg, 3.31 mmol) were dissolved in acetonitrile (20 mL). The reaction mixture was stirred and refluxed for 8 h under a nitrogen atmosphere, the resulting mixture was evaporated to dryness. After the solvent was evaporated under reduced pressure, the crude product was purified by silica gel column chromatography using DCM/MeOH (100:1, v/v) as eluent to get Compound 7 as pink solid (1.09 g, 85%). ^{1}H NMR (500 MHz, 298 K, DMSO-*d*$_6$) δ 8.99-8.98 (d, *J* = 5.0 Hz, 2H), 8.02-8.01 (d, *J* = 5.0 Hz, 2H), 7.88-7.87 (d, *J* = 7.0 Hz, 2H), 7.03-7.02 (d, *J* = 7.0 Hz, 2H), 5.10-5.05 (m, 1H), 5.00-4.99 (m, 2H), 4.58-4.57 (m, 2H), 2.61 (s, 3H), 1.29-1.27 (d, *J* = 5.5 Hz, 6H). ^{13}C NMR (125 MHz, 298 K, DMSO-*d*$_6$) δ 164.78, 161.15, 159.59, 144.38, 131.19, 128.25, 123.30, 114.59, 67.80, 66.35, 58.98, 21.73, 21.53. HR-MS (ESI): m/z calcd for $C_{18}H_{22}NO_3$ [M]$^+$: 300.1594; found: 300.1589.

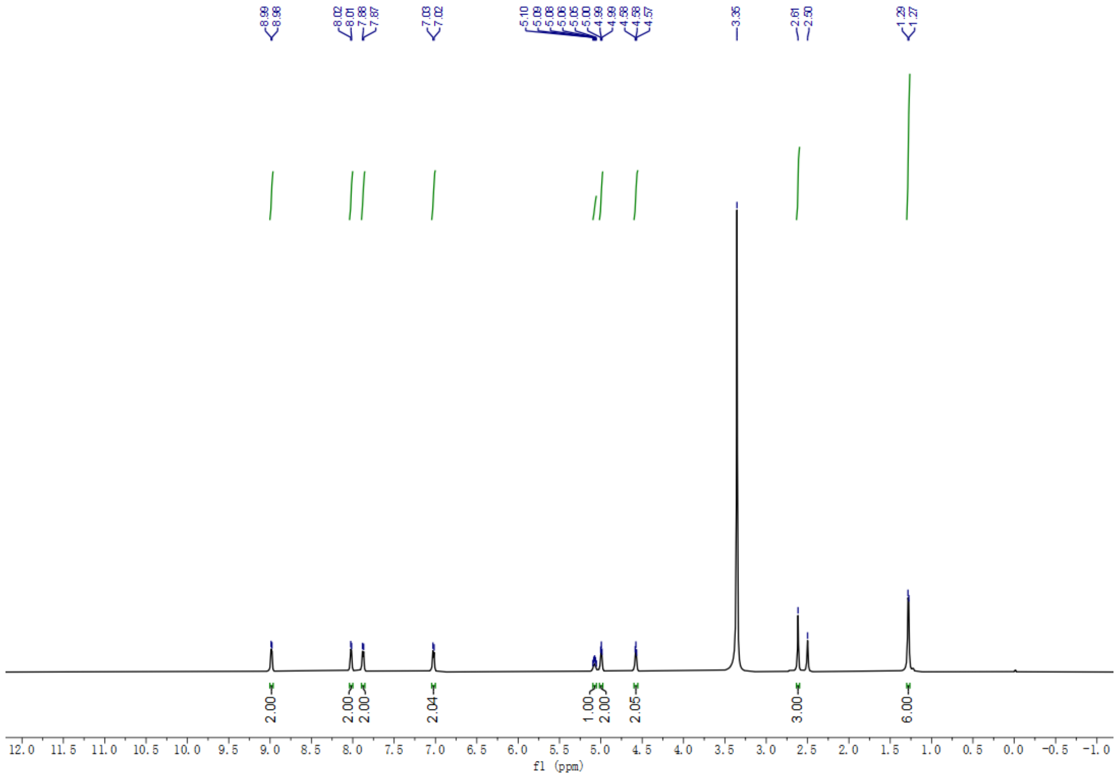

Supplementary Fig. 20 ^{1}H NMR spectrum of compound 7.

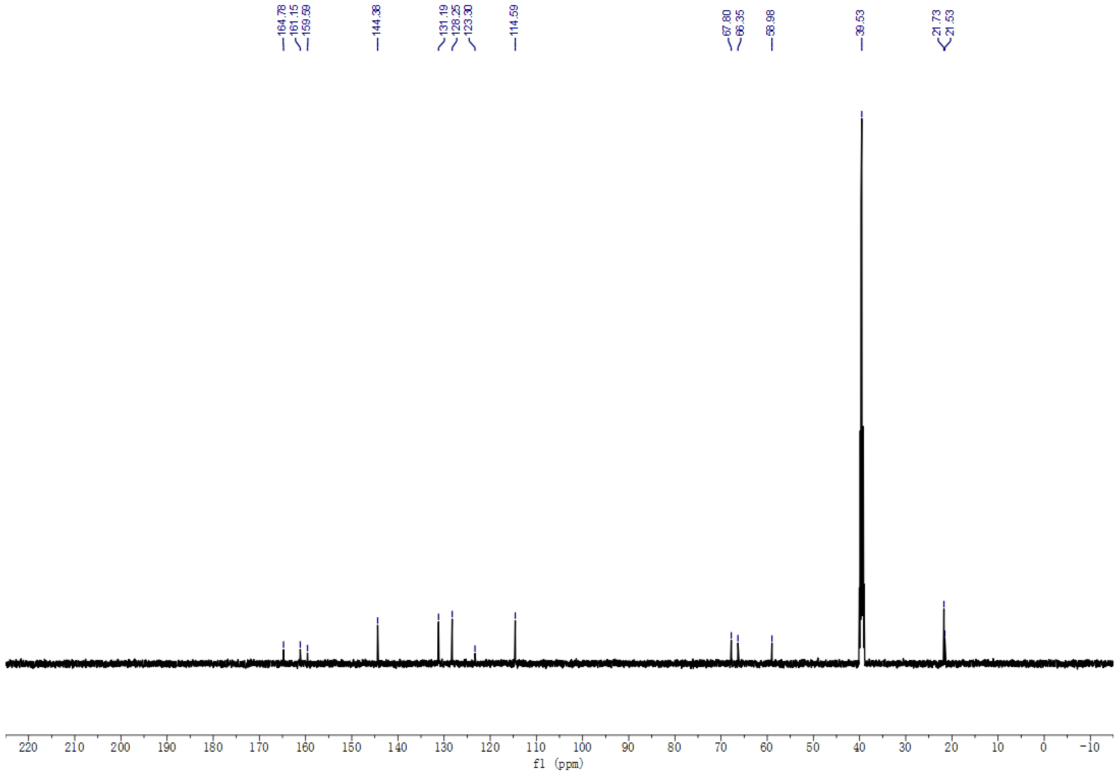

Supplementary Fig. 21 ^{13}C NMR spectrum of compound 7.

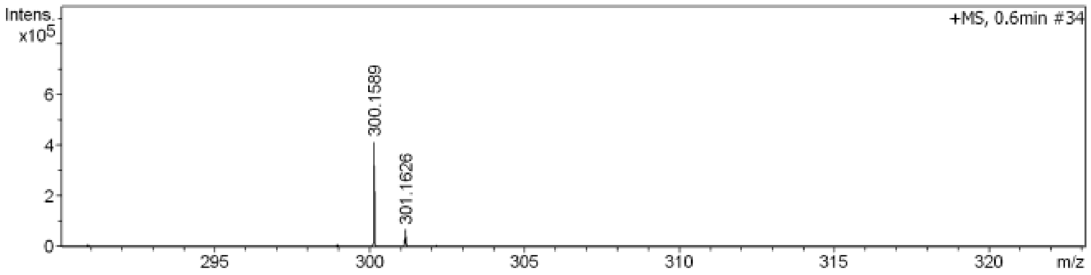

#	m/z	Res.	S/N	I	I %	FWHM
1	300.1589	23912	973.6	411732	100.0	0.0126
2	301.1626	18752	154.9	65468	15.9	0.0161
3	302.1656	17076	14.4	6092	1.5	0.0177

Meas. m/z	#	Ion Formula	m/z	err [ppm]	mSigma		Score	rdb	e^- Conf	N-Rule
300.1589	1	C18H22NO3	300.1594	1.8	25.6	1	100.00	8.5	even	ok

Supplementary Fig. 22 HR-ESI-MS spectrum of compound 7.

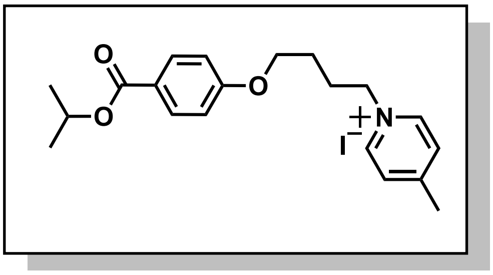

Synthesis of Compound 8: The synthesis of compound 8 was the same as compound 7 (1.16 g, 85%). ^{1}H NMR (500 MHz, 298 K, DMSO-d_6) δ 8.98-8.97 (dd, J_1 = 1.5 Hz, J_2 = 4.0 Hz, 2H), 8.01-8.00 (d, J = 5.0 Hz, 2H), 7.88-7.86 (d, J = 7.0 Hz, 2H), 7.03-7.00 (m, 2H), 5.10-5.06 (m, 1H), 4.64-4.61 (m, 2H), 4.09-4.07 (m, 2H), 2.60 (s, 3H), 2.09-2.04 (m, 2H), 1.76-1.72 (m, 2H), 1.29-1.28 (d, J = 5.0 Hz, 6H).^{13}C NMR (125 MHz, 298 K, DMSO-d_6) δ 164.85, 162.17, 158.83, 143.72, 131.10, 128.39, 122.45, 114.40, 67.60, 67.11, 59.55, 27.40, 25.06, 21.72, 21.40. HR-MS (ESI): m/z calcd for $C_{20}H_{26}NO_3$ $[M]^+$: 328.1907; found: 328.1903.

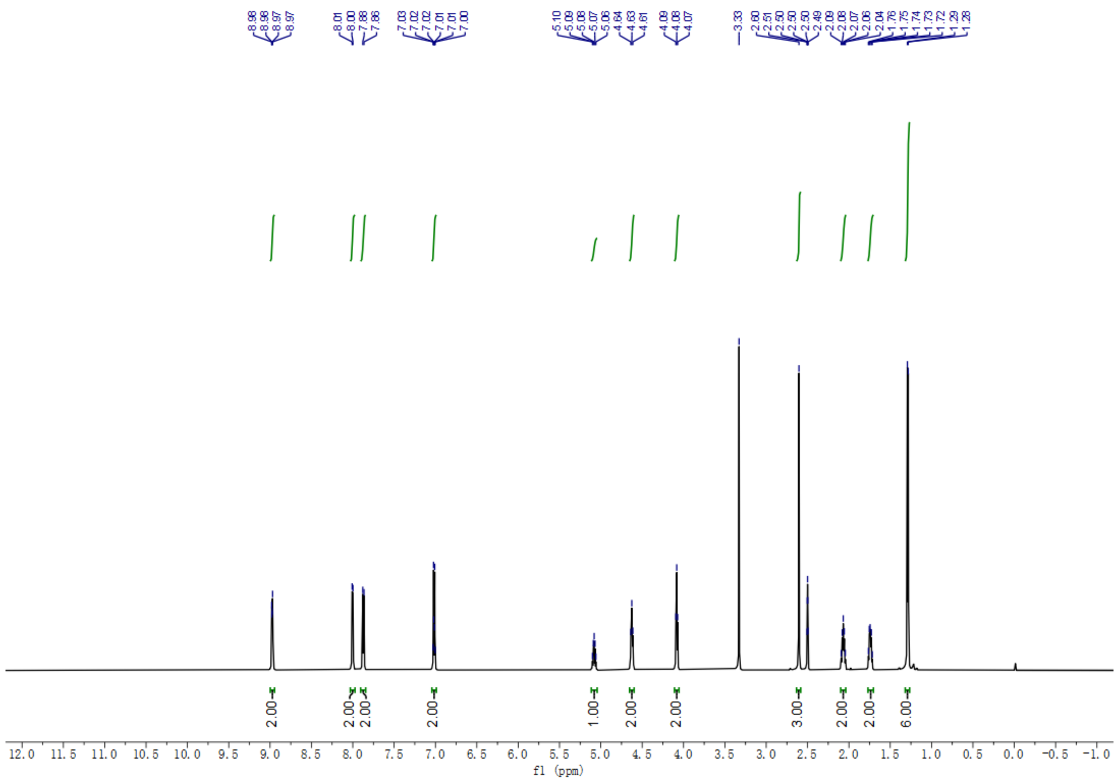

Supplementary Fig. 23 ^{1}H NMR spectrum of compound 8.

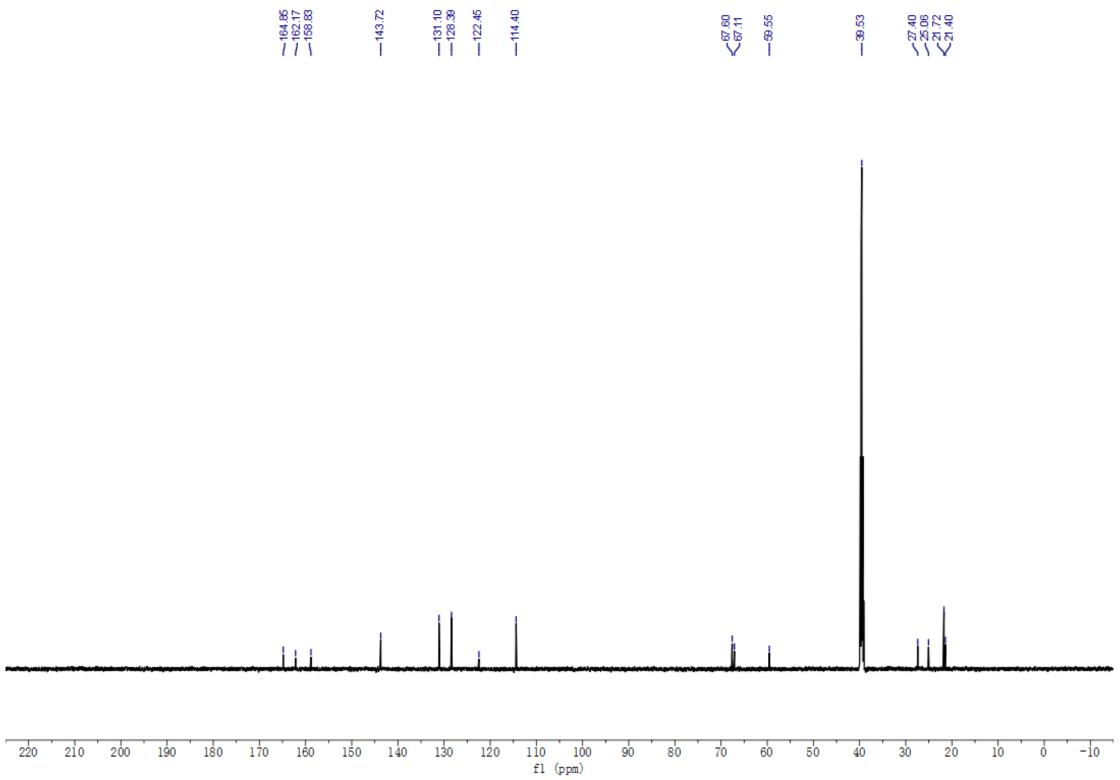

Supplementary Fig. 24 ^{13}C NMR spectrum of compound 8.

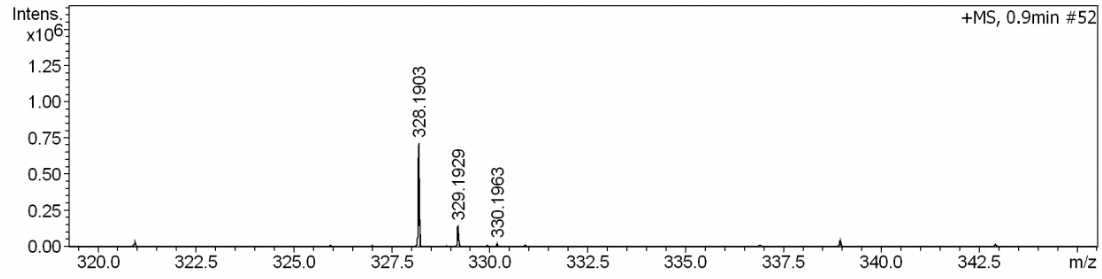

#	m/z	Res.	S/N	I	I %	FWHM
1	328.1903	20054	757.1	712988	100.0	0.0164
2	329.1929	14954	155.9	146900	20.6	0.0220
3	330.1963	17017	24.8	23388	3.3	0.0194

Meas. m/z	#	Ion Formula	m/z	err [ppm]	mSigma	#	Score	rdb	e⁻ Conf	N-Rule
328.1903	1	C20H26NO3	328.1907	1.4	10.5	1	100.00	8.5	even	ok

Supplementary Fig. 25 HR-ESI-MS spectrum of compound 8.

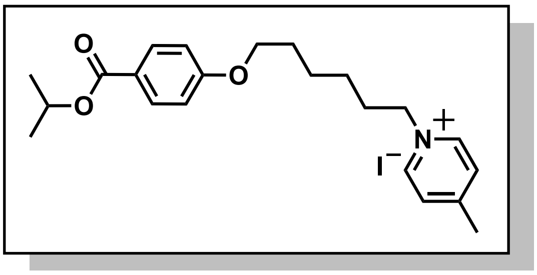

Synthesis of Compound 9: The synthesis of compound 9 was the same as compound 7 (1.23 g, 85%). ^{1}H NMR (500 MHz, 298 K, DMSO-d_6) δ 8.96-8.94 (m, 2H), 7.99-7.98 (d, *J* = 5.0 Hz, 2H), 7.88-7.86 (m, 2H), 7.01-6.99 (m, 2H), 5.10-5.06 (m, 1H), 4.56-4.53 (m, 2H), 4.03-4.01 (m, 2H), 2.60 (s, 3H), 1.94-1.89 (m, 2H), 1.74-1.69 (m, 2H), 1.47-1.42 (m, 2H), 1.34-1.30 (m, 2H), 1.29-1.28 (d, *J* = 5.0 Hz, 6H). ^{13}C NMR (125 MHz, 298 K, DMSO-d_6) δ 164.89, 162.39, 158.75, 143.68, 131.12, 128.35, 122.29, 114.34, 67.65, 67.58, 59.84, 30.43, 28.18, 25.05, 24.84, 21.73, 21.38. HR-MS (ESI): m/z calcd for $C_{22}H_{30}NO_3$ $[M]^+$: 356.2220; found: 356.2209.

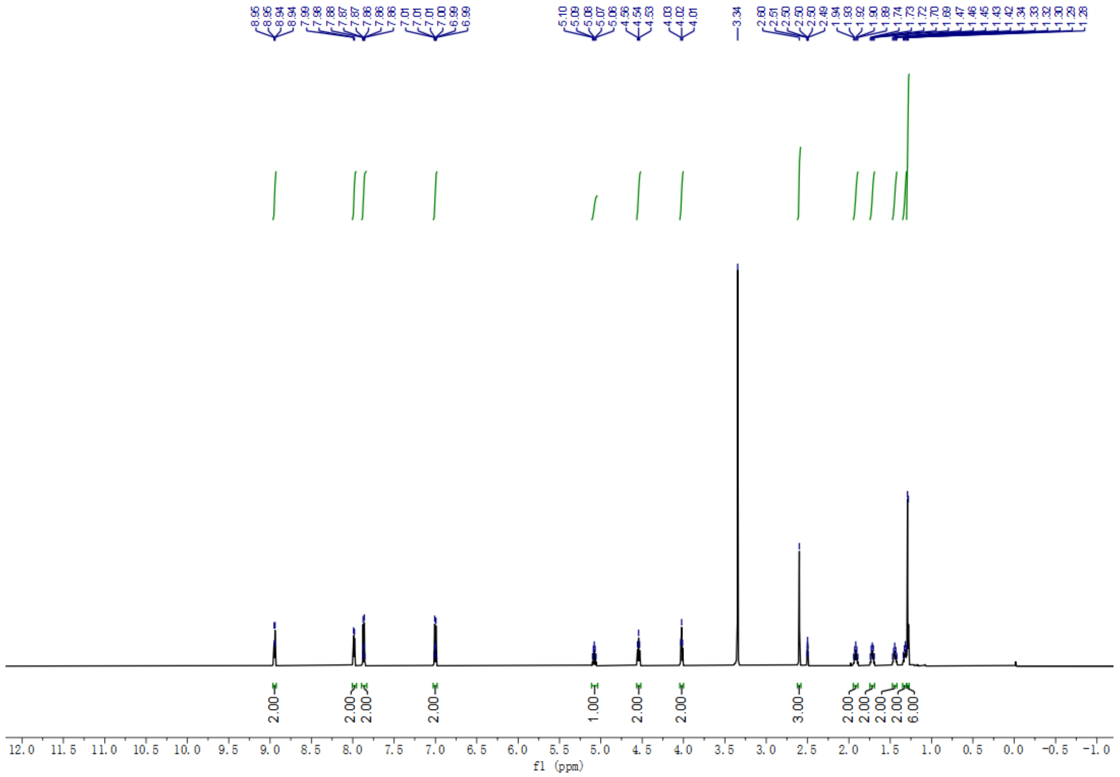

Supplementary Fig. 26 ^{1}H NMR spectrum of compound 9.

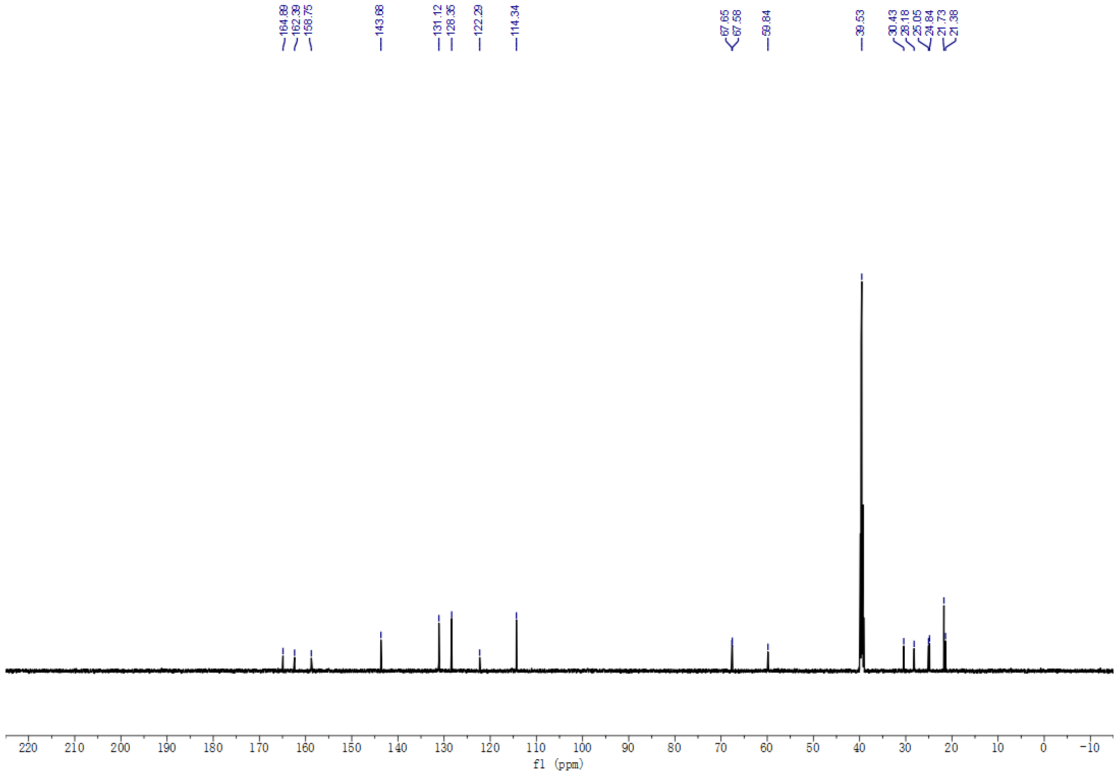

Supplementary Fig. 27 ^{13}C NMR spectrum of compound 9.

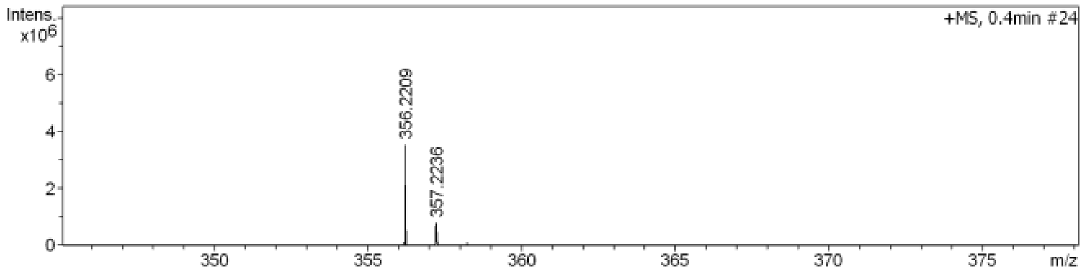

#	m/z	Res.	S/N	I	I %	FWHM
1	356.2209	23079	6787.3	3534092	100.0	0.0154
2	357.2236	29944	1527.0	795780	22.5	0.0119
3	358.2272	20917	156.4	81556	2.3	0.0171

Meas. m/z	#	Ion Formula	m/z	err [ppm]	mSigma	# mSigma	Score	rdb	e¯ Conf	N-Rule
356.2209	1	C22H30NO3	356.2220	3.2	12.2	2	100.00	8.5	even	ok

Supplementary Fig. 28 HR-ESI-MS spectrum of compound 9.

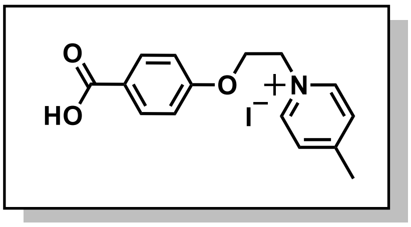

Synthesis of Compound 10: Compound 7 (214 mg, 0.5 mmol) was dissolved in 50 ml 10% KOH and 50 mL tetrahydrofuran. The reaction mixture was stirred and refluxed for 12 h under a nitrogen atmosphere, the resulting mixture was evaporated to remove the tetrahydrofuran. The pH of the water phase is adjusted to about 1 with dilute hydrochloric acid, and the solvent was evaporated under reduced pressure. The crude product was purified by silica gel column chromatography using DCM/MeOH(100:1, v/v) as eluent to get Compound 10 as blown solid (133 mg, 69%).^{1}H NMR (500 MHz, 298 K, DMSO-d_6) δ 12.62 (s, 1H), 8.98-8.96 (d, J = 8.0 Hz, 2H), 8.02-8.01 (d, J = 8.0 Hz, 2H), 7.88-7.86 (dd, J_1 = 2.5 Hz, J_2 = 8.0 Hz, 2H), 7.00-6.97 (dd, J_1 = 3.0 Hz, J_2 = 8.5 Hz, 2H), 4.99-4.97 (m, 2H), 4.57-4.55 (m, 2H), 2.62 (s, 3H). ^{13}C NMR (125 MHz, 298 K, DMSO-d_6) δ 166.85, 160.92, 159.55, 144.37, 131.37, 128.21, 123.93, 114.41, 66.27, 58.98, 21.49. HR-MS (ESI): m/z calcd for $C_{15}H_{16}NO_3$ $[M]^+$: 258.1125; found: 258.1131.

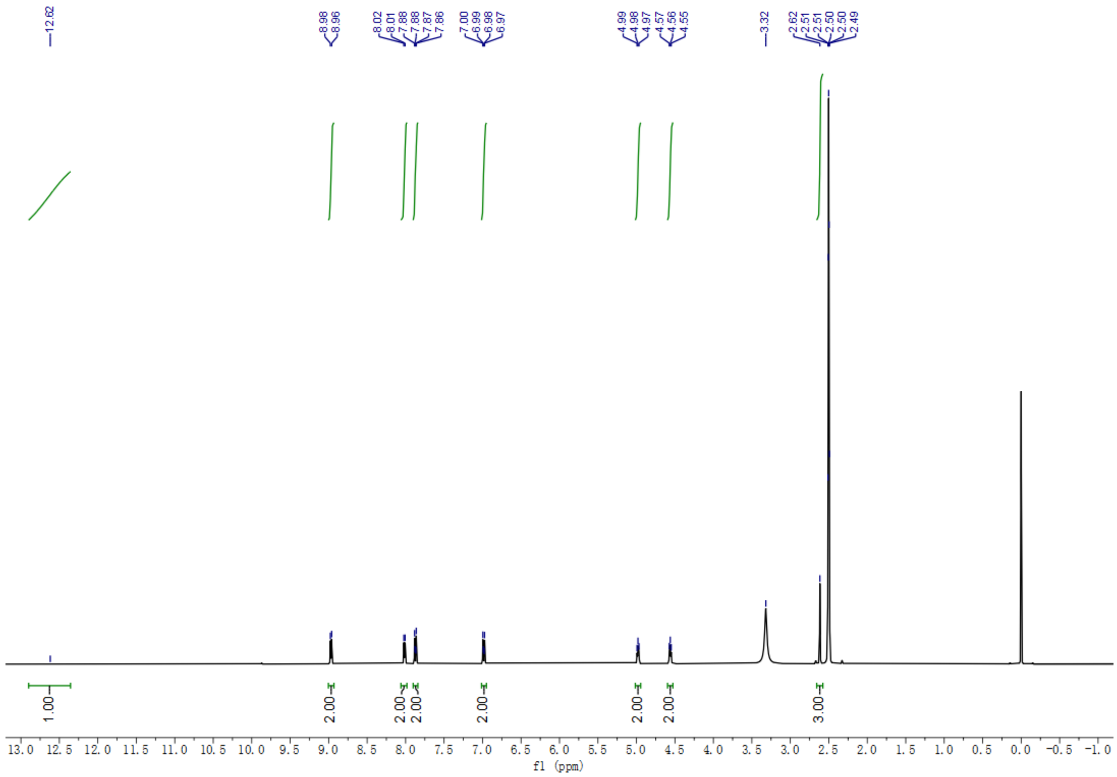

Supplementary Fig. 29 ^{1}H NMR spectrum of compound 10.

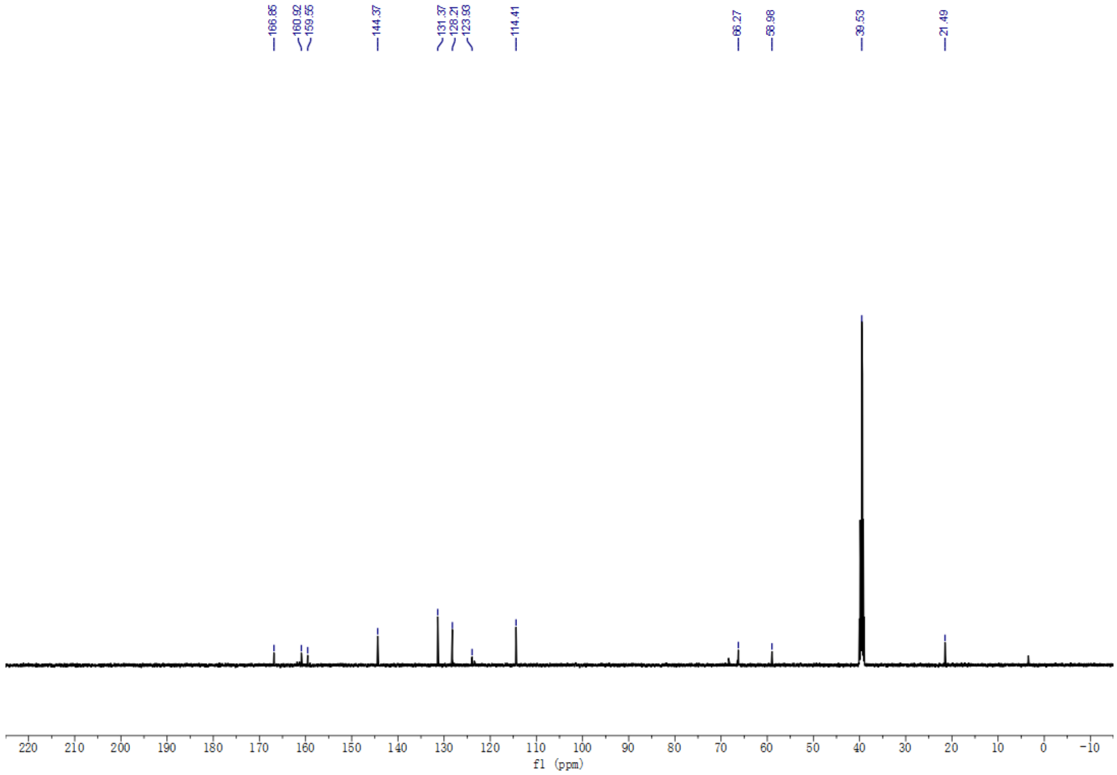

Supplementary Fig. 30 ^{13}C NMR spectrum of compound 10.

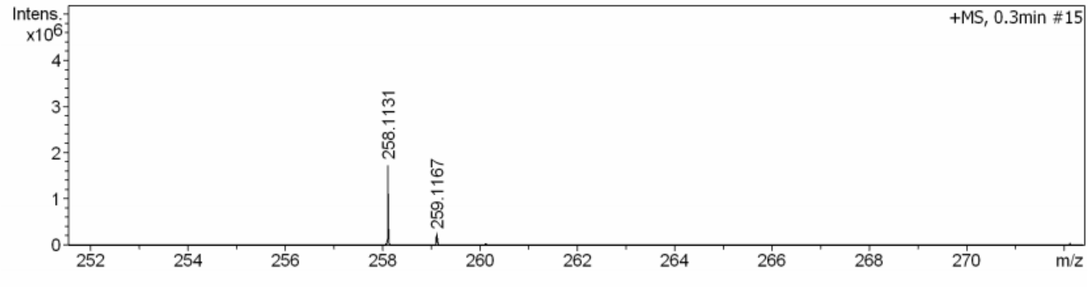

#	m/z	Res.	S/N	I	I %	FWHM
1	258.1131	27747	3439.5	1731156	100.0	0.0093
2	259.1167	16638	442.2	222836	12.9	0.0156
3	260.1202	18039	47.1	23708	1.4	0.0144

Meas. m/z	#	Ion Formula	m/z	err [ppm]	mSigma	#	Score	rdb	e⁻ Conf	N-Rule
258.1131	1	C15H16NO3	258.1125	-2.4	23.4	1	100.00	8.5	even	ok

Supplementary Fig. 31 HR-ESI-MS spectrum of compound 10.

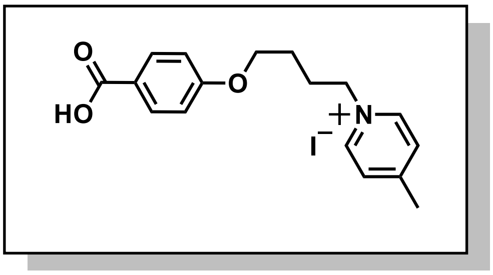

Synthesis of Compound 11: The synthesis of compound 11 was the same as compound 10 (147mg, 71%). ^{1}H NMR (500 MHz, 298 K, DMSO-d_6) δ 12.62 (s, 1H), 9.00-8.99 (d, J = 7.0 Hz, 2H), 8.01-7.99 (d, J = 6.5 Hz, 2H), 7.88-7.86 (dd, J_1 = 2.0 Hz, J_2 = 6.5 Hz, 2H), 7.00-6.99 (dd, J_1 = 2.5 Hz, J_2 = 7.0 Hz, 2H), 4.65-4.63 (m, 2H), 4.09-4.07 (m, 2H), 2.60 (s, 3H), 2.09-2.03 (m, 2H), 1.77-1.71 (m, 2H).^{13}C NMR (125 MHz, 298 K, DMSO-d_6) δ 166.93, 162.00, 158.81, 143.81, 131.31, 128.39, 122.99, 114.29, 67.09, 59.43, 27.48, 25.08, 21.40. HR-MS (ESI): m/z calcd for $C_{17}H_{20}NO_3$ $[M]^+$: 286.1438; found: 286.1444.

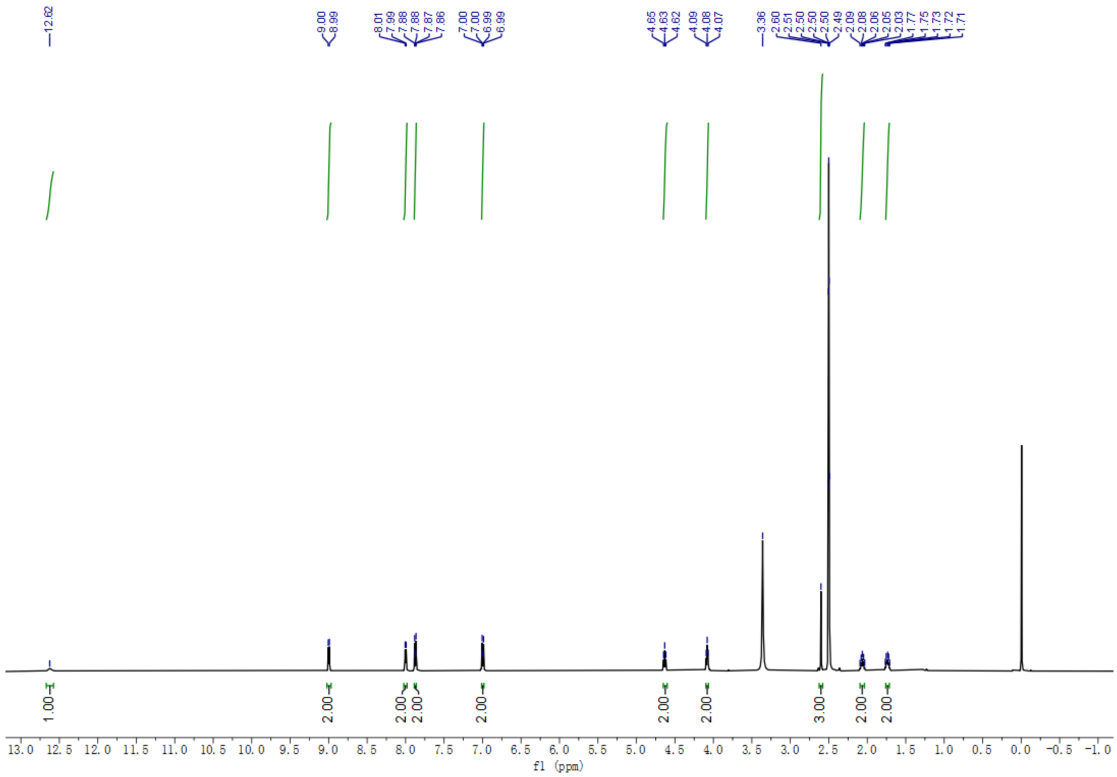

Supplementary Fig. 32 ^{1}H NMR spectrum of compound 11.

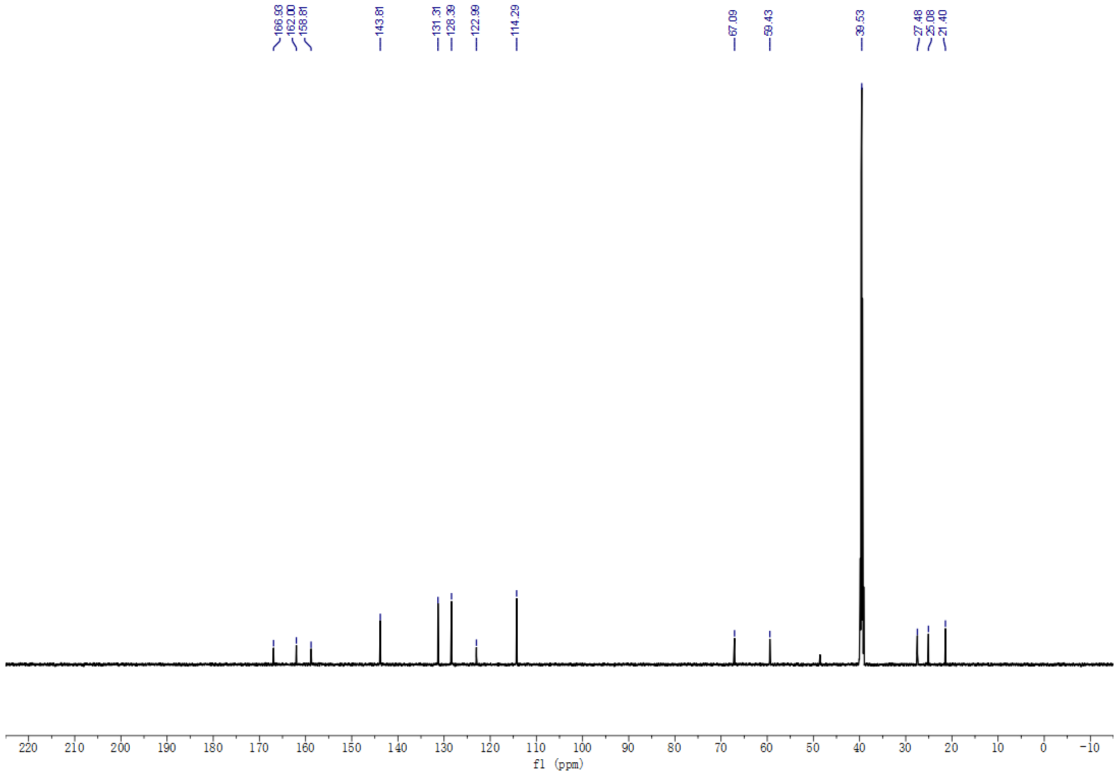

Supplementary Fig. 33 ^{13}C NMR spectrum of compound 11.

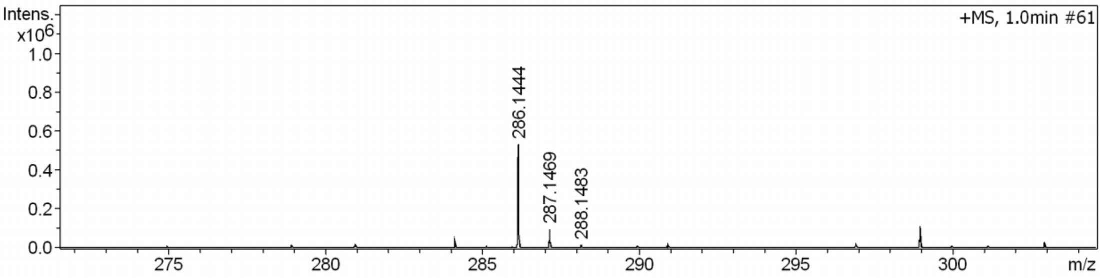

#	m/z	Res.	S/N	I	I %	FWHM
1	286.1444	18024	539.1	530564	100.0	0.0159
2	287.1469	14123	98.7	97052	18.3	0.0203
3	288.1483	15884	12.9	12688	2.4	0.0181

Meas. m/z	#	Ion Formula	m/z	err [ppm]	mSigma	#	Score	rdb	e⁻ Conf	N-Rule
286.1444	1	C17H20NO3	286.1438	-2.4	4.7	1	100.00	8.5	even	ok

Supplementary Fig. 34 HR-ESI-MS spectrum of compound 11.

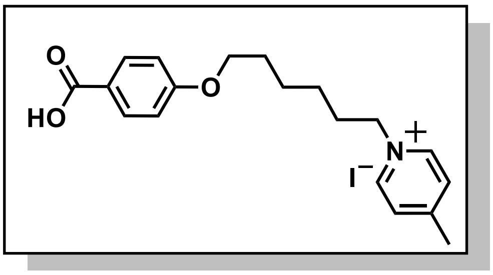

Synthesis of Compound 12: The synthesis of compound 12 was the same as compound 10 (154 mg, 70%). ^{1}H NMR (500 MHz, 298 K, DMSO-d_6) δ 12.60 (s, 1H), 8.94-8.93 (d, J = 7.0 Hz, 2H), 7.99-7.97 (d, J = 6.5 Hz, 2H), 7.88-7.86 (dd, J_1 = 2.0 Hz, J_2 = 6.5 Hz, 2H), 6.99-6.97 (dd, J_1 = 2.5 Hz, J_2 = 7.0 Hz, 2H), 4.55-4.52 (m, 2H), 4.04-4.01 (m, 2H), 2.60 (s, 3H), 1.95-1.89 (m, 2H), 1.75-1.69 (m, 2H), 1.48-1.42 (m, 2H), 1.35-1.29 (m, 2H). ^{13}C NMR (125 MHz, 298 K, DMSO-d_6) δ 166.97, 162.21, 158.73, 143.78, 131.35, 128.35, 122.85, 114.22, 67.59, 59.77, 30.50, 28.21, 25.07, 24.87, 21.37. MS (ESI): m/z calcd for $C_{19}H_{24}NO_3$ $[M]^+$: 314.1751; found: 314.1743.

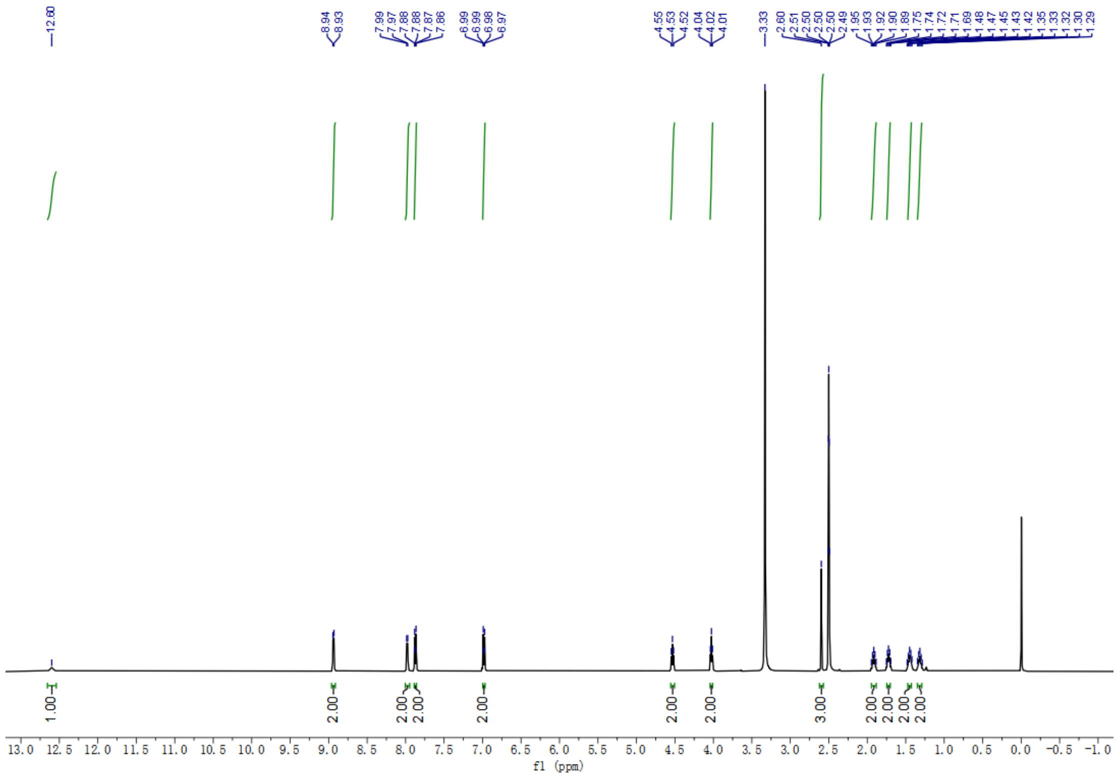

Supplementary Fig. 35 ^{1}H NMR spectrum of compound 12.

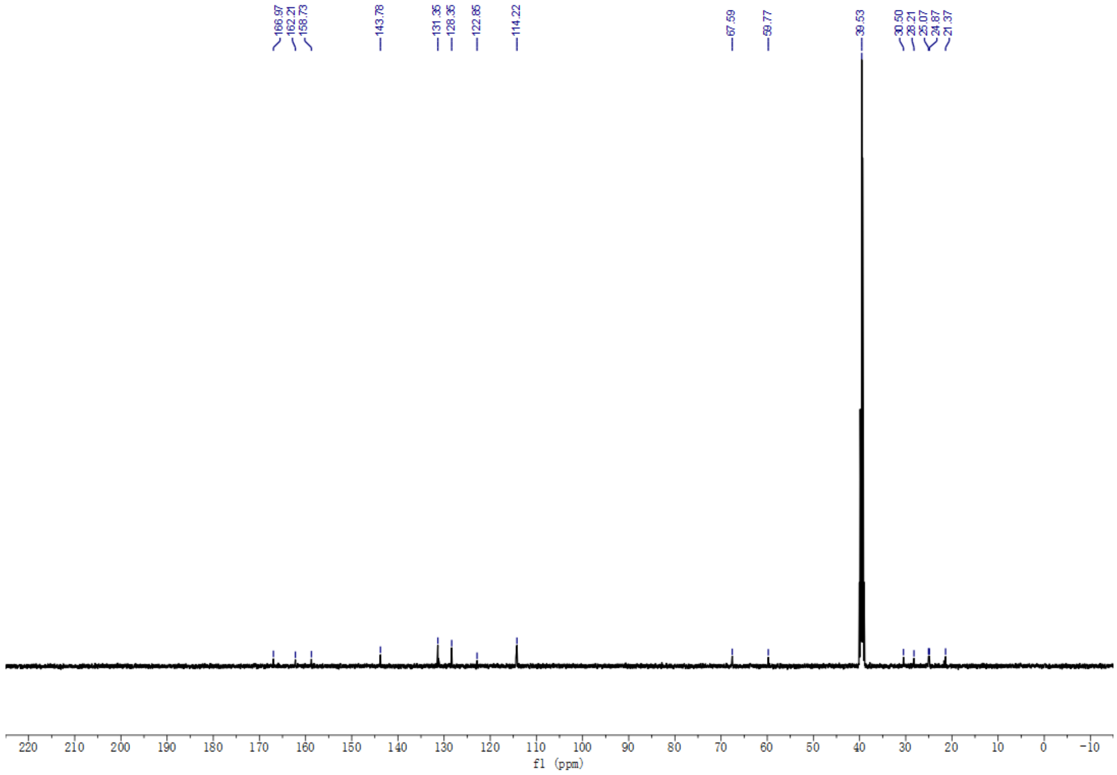

Supplementary Fig. 36 ^{13}C NMR spectrum of compound 12.

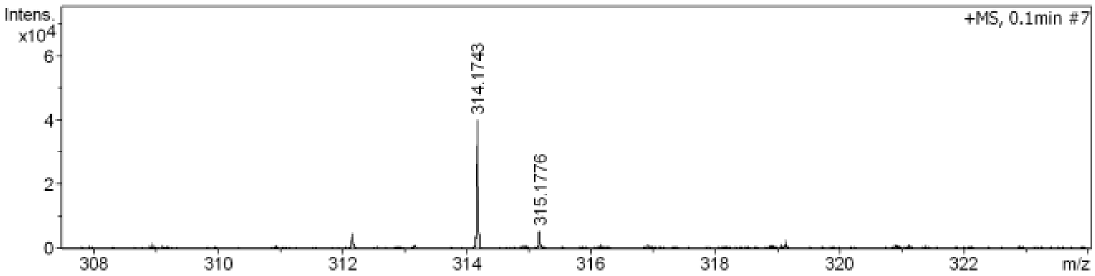

#	m/z	Res.	S/N	I	I %	FWHM
1	314.1743	19862	77.6	39924	100.0	0.0158
2	315.1776	18357	10.6	5476	13.7	0.0172

Meas. m/z	#	Ion Formula	m/z	err [ppm]	mSigma		Score	rdb	e⁻ Conf	N-Rule
314.1743	1	C19H24NO3	314.1751	2.5	46.7	4	100.00	8.5	even	ok

Supplementary Fig. 37 HR-ESI-MS spectrum of compound 12.

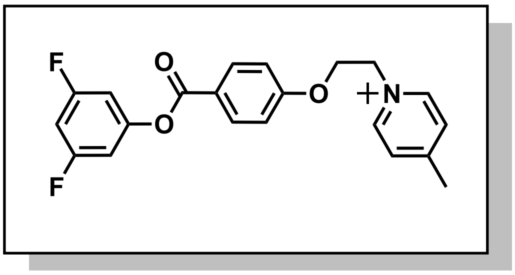

Synthesis of Compound 13: A mixture of Compound 10 (192 mg, 0.5 mmol), 3,5-difluorophenol (78 mg, 0.6 mmol), EDCI (96 mg，0.5 mmol), and DMAP (6.1mg, 0.05 mmol) in tetrahydrofuran (20 mL) was stirred and refluxed for 12 h under a nitrogen atmosphere. After being cooled to room temperature, the reaction mixture was diluted with EA, washed with brine, and dried over Na_2SO_4. After removal of solvent, Product 13 was isolated as an orange solid (126 mg, 48%) after column chromatography on silica gel using DCM/MeOH (100:1, v/v) as eluent.^{1}H NMR (500 MHz, 298 K, DMSO-d_6) δ 9.01-8.99 (dd, J_1 = 1.5 Hz, J_2 = 4.0 Hz, 2H), 8.22-8.21 (dd, J_1 = 1.5 Hz, J_2 = 5.0 Hz, 1H), 8.08-8.06 (dd, J_1 = 2.0 Hz, J_2 = 5.5 Hz, 2H), 8.04-8.03 (d, J = 5.5 Hz, 2H), 7.26-7.22 (m, 1H), 7.20-7.18 (m, 1H), 7.14-7.11 (m, 1H), 6.98-6.96 (m, 1H), 5.03-5.01 (m, 2H), 4.64-4.62 (m, 2H), 2.63 (s, 3H). ^{13}C NMR (125 MHz, 298 K, DMSO-d_6) δ 163.40, 163.22, 162.08, 159.60, 156.86, 144.41, 139.45, 132.25, 128.23, 121.16, 114.92, 106.96, 66.46, 58.92, 21.50. HR-MS (ESI): m/z calcd for $C_{21}H_{18}F_2NO_3$ $[M]^+$: 370.1249; found: 370.1238.

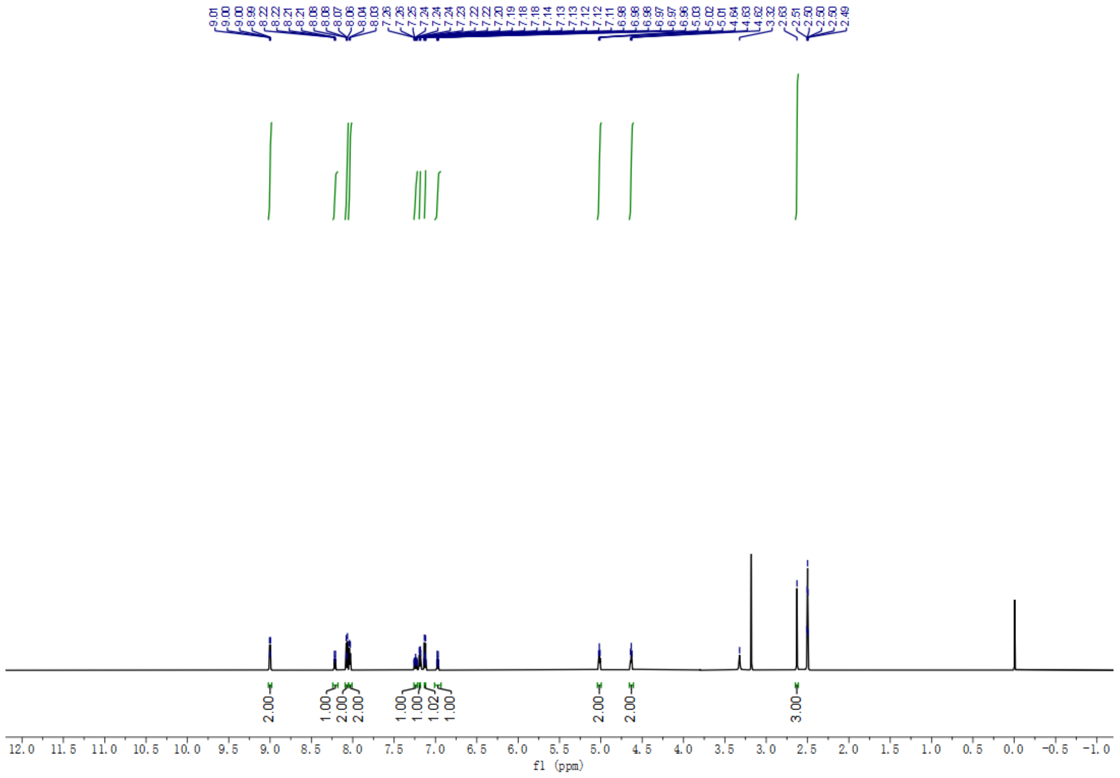

Supplementary Fig. 38 ^{1}H NMR spectrum of compound 13.

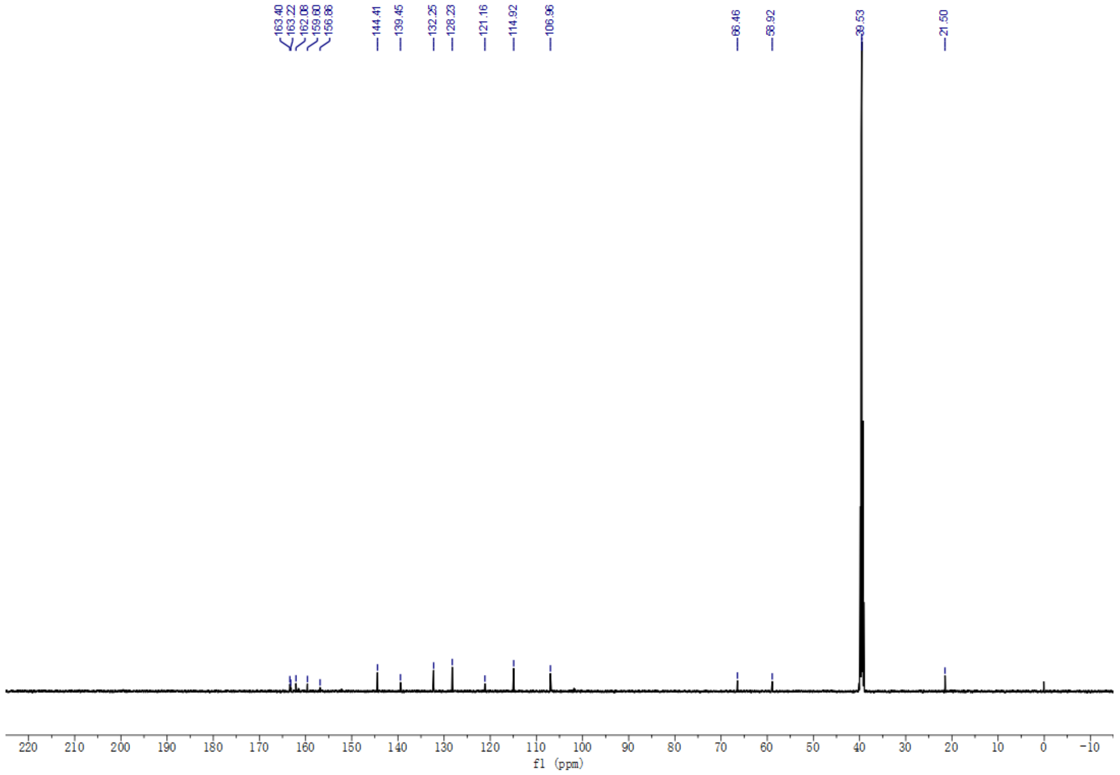

Supplementary Fig. 39 ^{13}C NMR spectrum of compound 13.

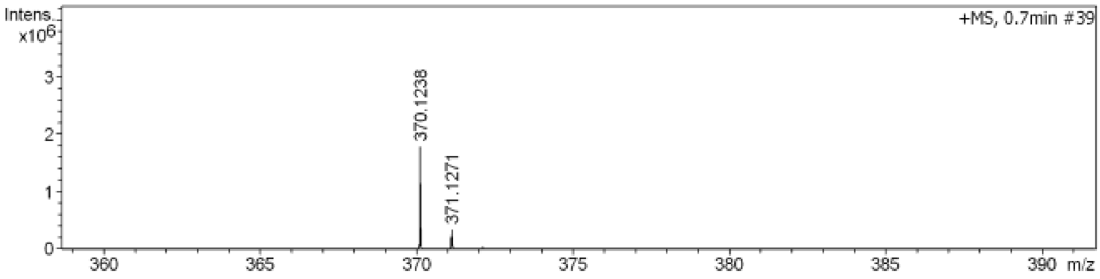

#	m/z	Res.	S/N	I	I %	FWHM
1	370.1238	34219	4105.7	1783904	100.0	0.0108
2	371.1271	23587	768.7	334060	18.7	0.0157
3	372.1308	19910	76.0	33008	1.9	0.0187

Meas. m/z	#	Ion Formula	m/z	err [ppm]	mSigma	#	Score	rdb	e⁻ Conf	N-Rule
370.1238	1	C21H18F2NO3	370.1249	3.0	28.1	9	39.30	12.5	even	ok

Supplementary Fig. 40 HR-ESI-MS spectrum of compound 13.

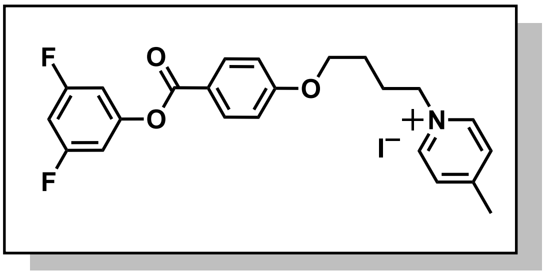

Synthesis of Compound 14: The synthesis of compound 14 was the same as compound 13 (120 mg, 50%). ^{1}H NMR (500 MHz, 298 K, 298 K,DMSO-d_6) δ 8.96-8.94 (d, J = 6.5 Hz, 2H), 8.08-8.06 (dd, J_1 = 2.0 Hz, J_2 = 6.5 Hz, 2H), 8.01-8.00 (d, J = 6.5 Hz, 2H), 7.27-7.22 (m, 1H), 7.20-7,18 (dd, J_1 = 2.5 Hz, J_2 = 8.0 Hz, 2H), 7.12-7.11 (dd, J_1 = 2.0 Hz, J_2 = 6.5 Hz, 2H), 4.63-4.60 (m, 2H), 4.15-4.13 (m, 2H), 2.61 (s, 3H), 2.11-2.05 (m, 2H), 1.79-1.74 (m, 2H).^{13}C NMR (125 MHz, 298 K, DMSO-d_6) δ163.51, 163.17, 158.89, 152.24, 143.76, 132.22, 128.42, 120.32, 114.79, 106.96, 106.90, 106.67, 67.34, 59.59, 39.53, 27.42, 25.07, 21.41. HR-MS (ESI): m/z calcd for $C_{23}H_{22}F_2NO_3$ $[M]^+$: 398.1562; found: 398.1568.

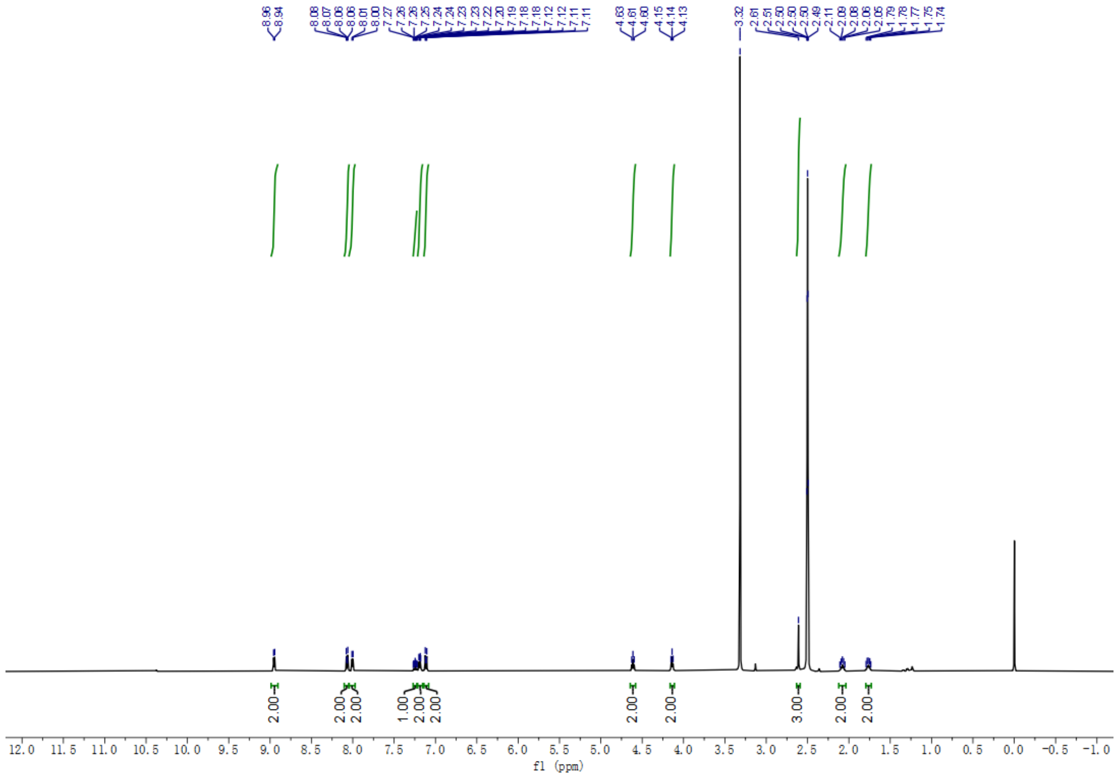

Supplementary Fig. 41 ^{1}H NMR spectrum of compound 14.

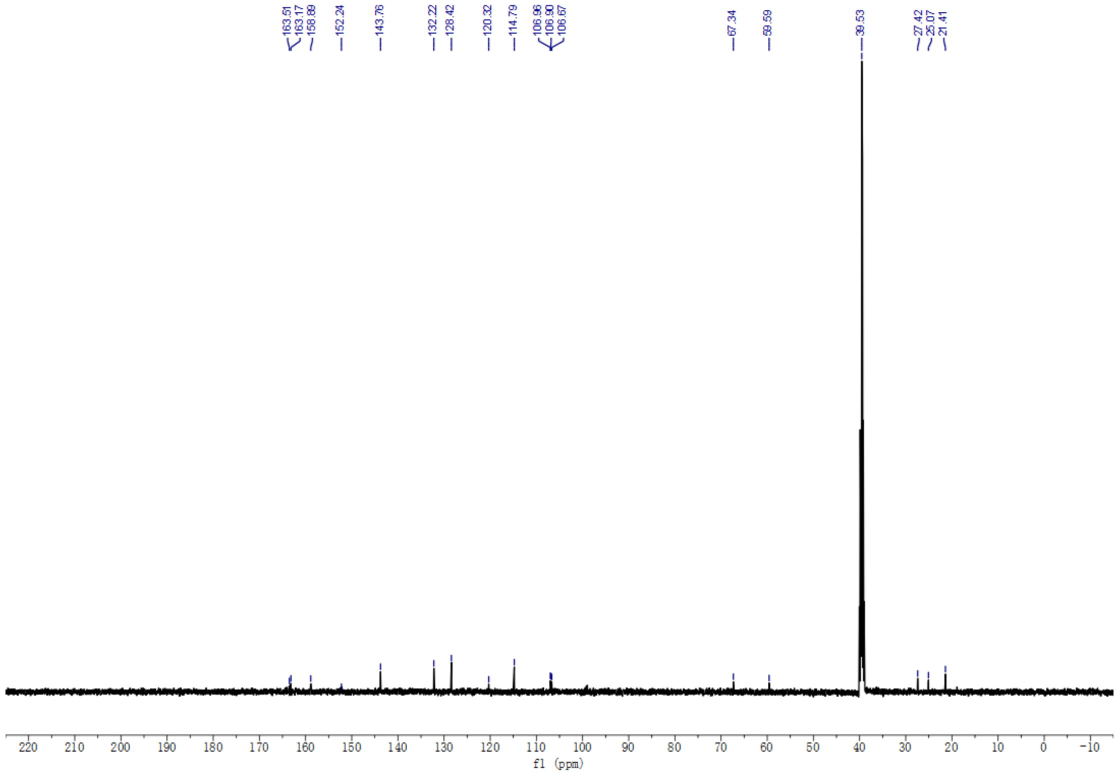

Supplementary Fig. 42 ^{13}C NMR spectrum of compound 14.

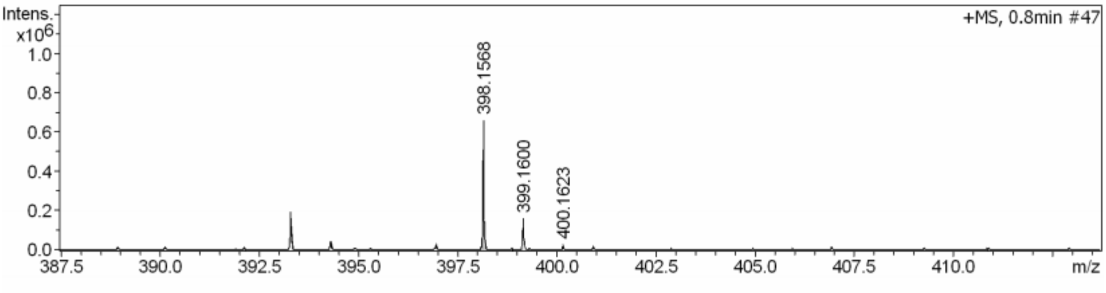

#	m/z	Res.	S/N	I	I %	FWHM
1	398.1568	19841	695.5	659372	100.0	0.0201
2	399.1600	14328	170.6	161728	24.5	0.0279
3	400.1623	16193	26.6	25212	3.8	0.0247

Meas. m/z	#	Ion Formula	m/z	err [ppm]	mSigma	#	Score	rdb	e⁻ Conf	N-Rule
398.1568	1	C23H22F2NO3	398.1562	-1.5	5.5	2	100.00	12.5	even	ok

Supplementary Fig. 43 HR-ESI-MS spectrum of compound 14.

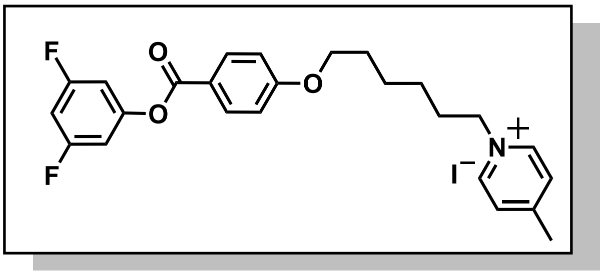

Synthesis of Compound 15: The synthesis of compound 15 was the same as compound 13 (138 mg, 50%). ^{1}H NMR (500 MHz, 298 K, DMSO-d_6) δ 8.94-8.93 (dd, J_1 = 2.0 Hz, J_2 = 5.0 Hz, 2H), 8.07-8.05 (dd, J_1 = 2.0 Hz, J_2 = 6.5 Hz, 2H), 8.00-7.98 (d, J = 7.0 Hz, 2H), 7.26-7.22 (m, 1H), 7.20-7.18 (dd, J_1 = 2.5 Hz, J_2 = 8.0 Hz, 2H), 7.12-7.10 (dd, J_1 = 2.0 Hz, J_2 = 7.0 Hz, 2H), 4.55-4.52 (m, 2H), 4.10-4.08 (m, 2H), 2.61 (s, 3H), 1.96-1.90 (m, 2H), 1.78-1.72 (m, 2H), 1.50-1.44 (m, 2H), 1.36-1.30 (m, 2H). ^{13}C NMR (125 MHz, 298 K, DMSO-d_6) δ 163.52, 163.37, 158.78, 143.71, 135.32, 132.23, 128.36, 125.73, 120.17, 114.73, 106.89, 101.78, 67.87, 59.87, 30.44, 28.17, 25.09, 24.85, 21.36. HR-MS (ESI): m/z calcd for $C_{25}H_{26}F_2NO_3$ $[M]^+$: 426.1875; found: 426.1880.

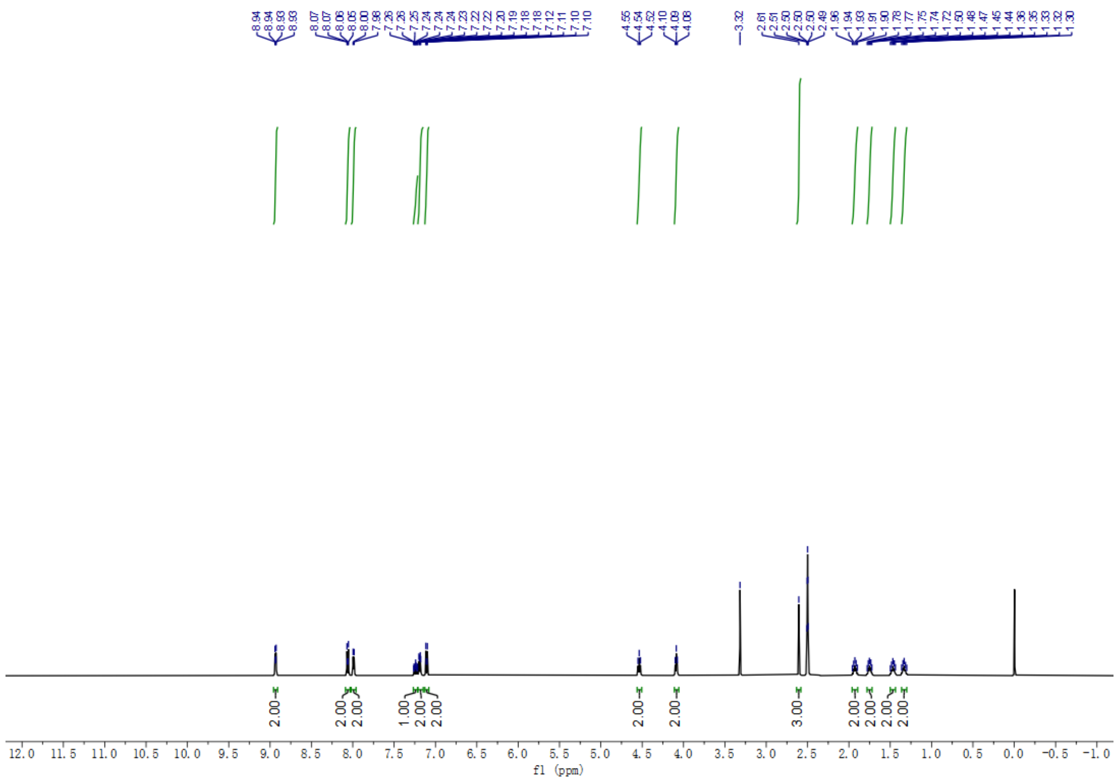

Supplementary Fig. 44 ^{1}H NMR spectrum of compound 15.

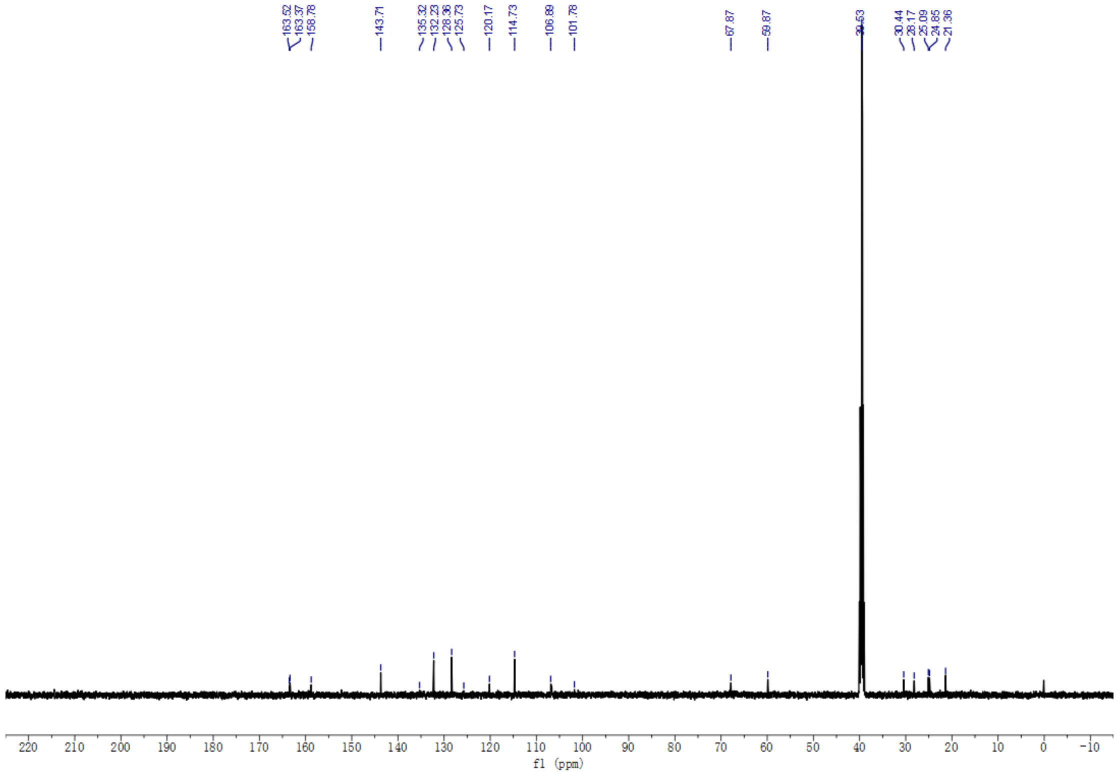

Supplementary Fig. 45 ^{13}C NMR spectrum of compound 15.

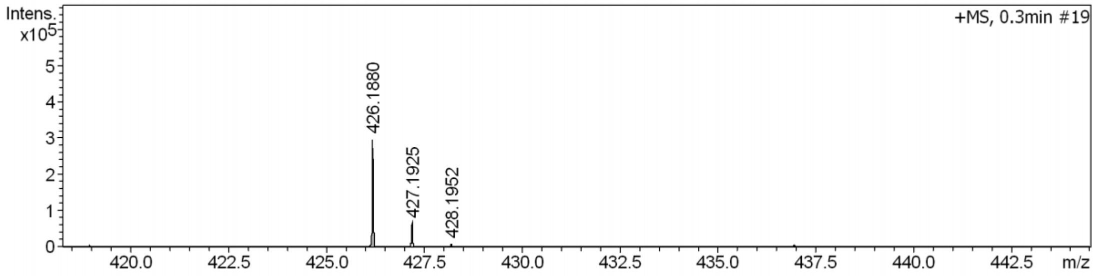

#	m/z	Res.	S/N	I	I %	FWHM
1	426.1880	24296	711.2	296740	100.0	0.0175
2	427.1925	19892	155.0	64664	21.8	0.0215
3	428.1952	17849	19.1	7956	2.7	0.0240

Meas. m/z	#	Ion Formula	m/z	err [ppm]	mSigma		Score	rdb	e⁻ Conf	N-Rule
426.1880	1	C25H26F2NO3	426.1875	-1.2	31.3	10	60.17	12.5	even	ok

Supplementary Fig. 46 HR-ESI-MS spectrum of compound 15.

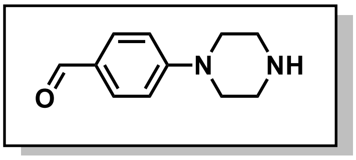

Synthesis of Compound 16: 4-Fluorobenzaldehyde (1.24 g, 10 mmol), K_2CO_3 (2.76 g, 20 mmol) and piperazine (1.72 g, 20 mmol) were dissolved in acetonitrile (30 mL). The reaction mixture was stirred and refluxed for 8 h under a nitrogen atmosphere, the resulting mixture was evaporated to dryness. After the solvent was evaporated under reduced pressure, the crude product was purified by silica gel column chromatography using DCM/MeOH(100:1, v/v) as eluent to get Compound 16 as orange solid (1.48 g, 78%).^{1}H NMR (500 MHz, 298 K, Chloroform-*d*) δ 9.76 (s, 1H), 7.74-7.73 (dd, J_1 = 2.0 Hz, J_2 = 7.0 Hz, 2H), 6.91-6.89 (dd, J_1 = 2.0 Hz, J_2 = 6.5 Hz, 2H), 3.38-3.36 (m, 4H), 3.04-3.02 (m, 4H), 2.87 (s, 1H).^{13}C NMR (125 MHz, 298 K, Chloroform-d) δ 190.40, 155.26, 131.81, 127.18, 113.57, 47.97, 45.59. HR-MS (ESI): m/z calcd for $C_{11}H_{15}N_2O$ $[M+H]^+$: 191.1179; found: 191.1183.

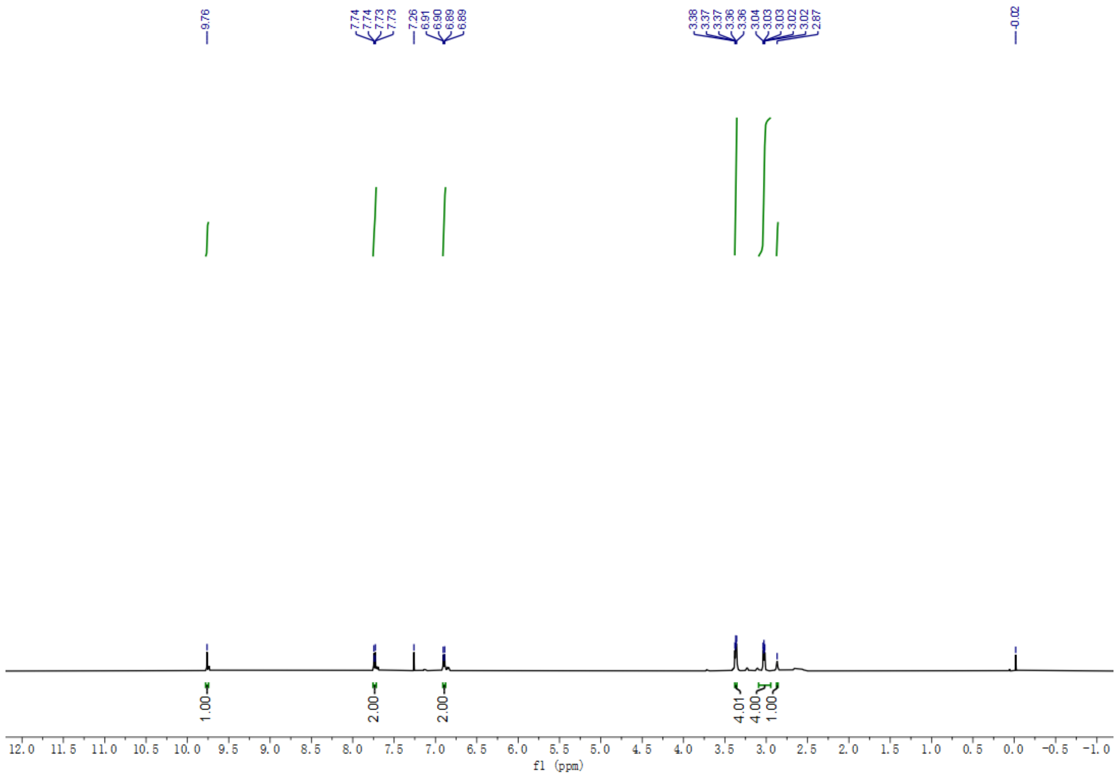

Supplementary Fig. 47 ^{1}H NMR spectrum of compound 16.

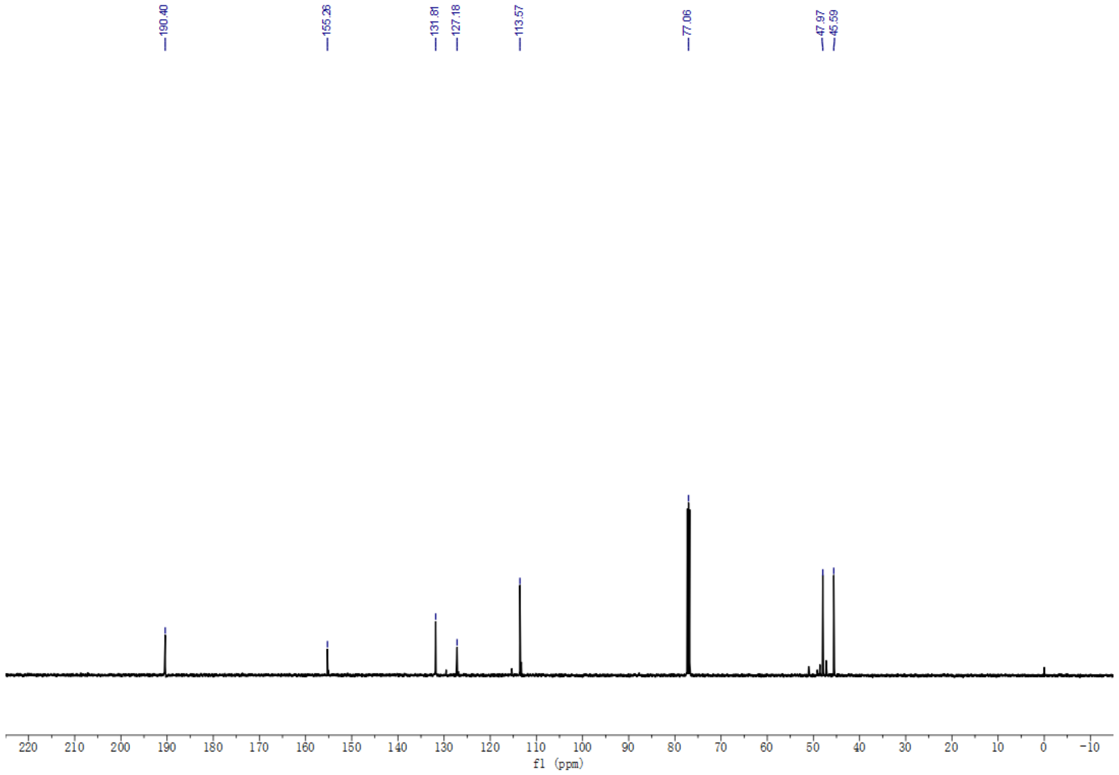

Supplementary Fig. 48 ^{13}C NMR spectrum of compound 16.

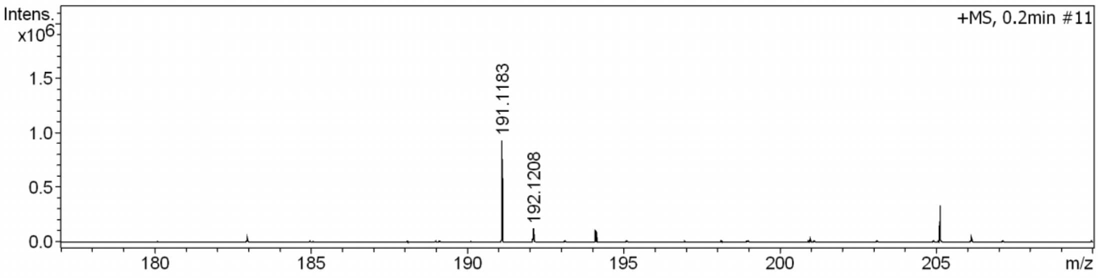

#	m/z	Res.	S/N	I	I %	FWHM
1	191.1183	17330	1123.7	929708	100.0	0.0110
2	192.1208	12769	146.1	121552	13.1	0.0150
3	193.1220	8593	12.2	10180	1.1	0.0225

Meas. m/z	#	Ion Formula	m/z	err [ppm]	mSigma	#	Score	rdb	e⁻ Conf	N-Rule
191.1183	1	C11H15N2O	191.1179	-2.0	1.6	1	100.00	5.5	even	ok

Supplementary Fig. 49 HR-ESI-MS spectrum of compound 16.

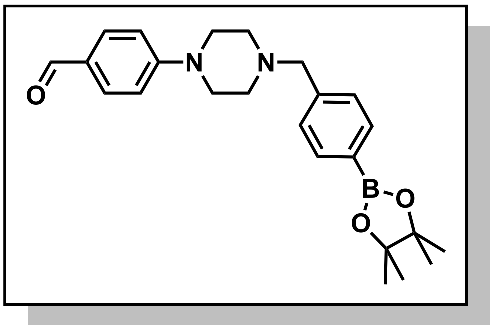

Synthesis of Compound 17: Compound 6 (570 mg, 3.0 mmol), (4-bromomethylphenyl)boronic acid (870 mg, 3.0 mmol) and K_2CO_3 (1.24 g, 9.0 mmol) were dissolved in acetonitrile (20 mL). The reaction mixture was stirred and refluxed for 8 h under a nitrogen atmosphere, the resulting mixture was evaporated to dryness. After the solvent was evaporated under reduced pressure, the crude product was purified by silica gel column chromatography using PE/EA(20:1, v/v) as eluent to get Compound 17 as yellow solid (975 mg, 80%).^{1}H NMR (500 MHz, 298 K, $CDCl_3$-*d*) δ 9.77 (s, 1 H), 7.80-7.78 (dd, J_1 = 1.5 Hz, J_2 = 6.0 Hz, 2H), 7.74-7.73 (dd, J_1 = 2.0 Hz, J_2 = 7.0 Hz, 2H), 7.37-7.36 (d, J = 8.0 Hz, 2 H), 6.90-6.88 (dd, J_1 = 2.0 Hz, J_2 = 7.0 Hz, 2H), 3.58 (s, 2H), 3.41-3.39 (m, 4H), 2.59-2.57 (m, 4H), 1.35 (s, 12H).^{13}C NMR (125 MHz, 298 K, $CDCl_3$-*d*) δ 190.40, 155.04, 134.88, 131.85, 128.56, 127.07, 113.50, 83.81, 83.81, 62.96, 52.65, 47.07, 24.90. HR-MS (ESI): m/z calcd for $C_{24}H_{31}BN_2NaO_3$ $[M+Na]^+$: 429.2324; found: 429.2332.

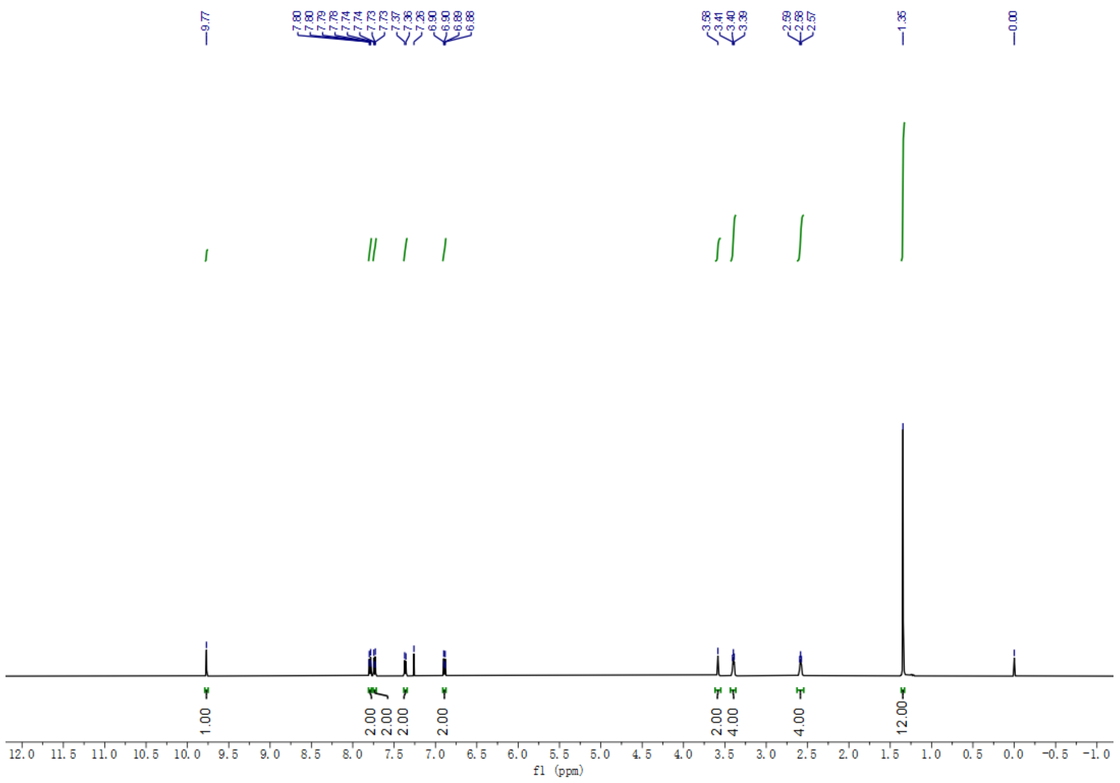

Supplementary Fig. 50 ^{1}H NMR spectrum of compound 17.

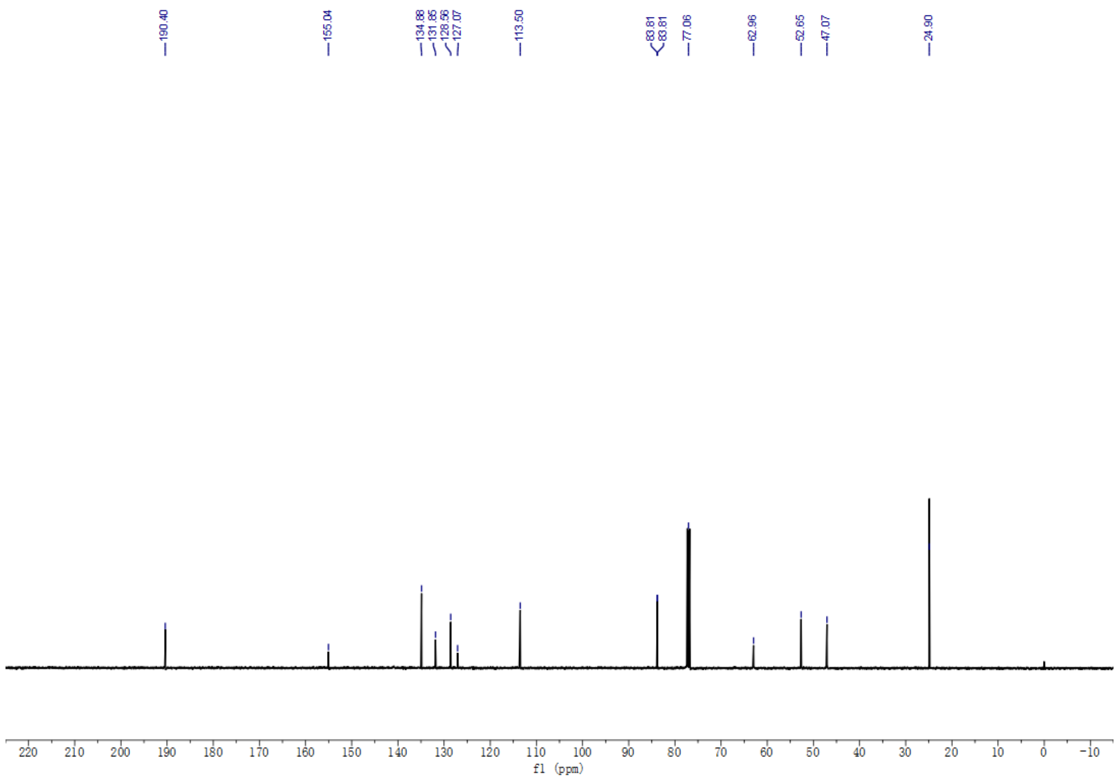

Supplementary Fig. 51 ^{13}C NMR spectrum of compound 17.

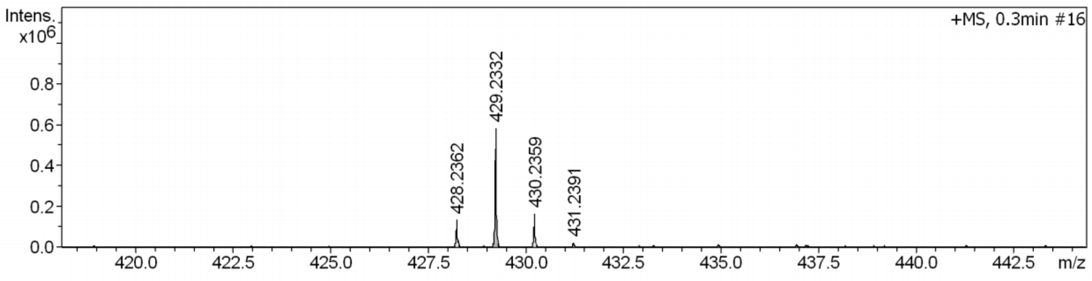

#	m/z	Res.	S/N	I	I %	FWHM
1	428.2362	15390	139.6	135944	23.2	0.0278
2	429.2332	18664	601.4	584936	100.0	0.0230
3	430.2359	15938	172.0	167072	28.6	0.0270
4	431.2391	12435	23.2	22540	3.9	0.0347

Meas. m/z	#	Ion Formula	m/z	err [ppm]	mSigma	#	Score	rdb	e⁻ Conf	N-Rule
429.2332	1	C24H31BN2NaO3	429.2324	-1.8	9.7	1	100.00	10.5	even	ok

Supplementary Fig. 52 HR-ESI-MS spectrum of compound 17.

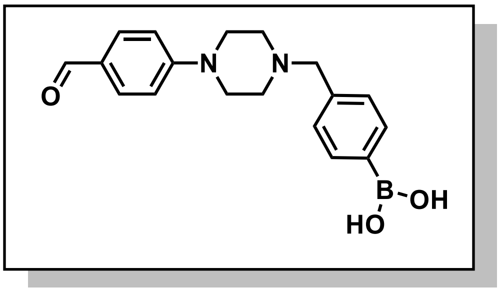

Synthesis of Compound 18: Compound 17 (406 mg, 1.0 mmol) and $NaIO_4$ (640mg, 3 mmol) were dissolved in tetrahydrofuran (10 mL). The reaction mixture was stirred at room temperature for 30 min, then 1.4 ml of 1 M dilute hydrochloric acid was added, and stirred at room temperature for 12 h. The solvent was removed in vacuo, water was added, and the product was extracted with EtOAc. The organic layers were combined, dried over Na_2SO_4, and the solvent was removed in vacuo. the crude product was purified by silica gel column chromatography using DCM/MeOH (20:1, v/v) as eluent to get Compound 18 as green solid (194 mg, 60%). ^{1}H NMR (500 MHz, 298 K, DMSO-d_6) δ 9.71 (s, 1H), 7.98 (s, 1H), 7.77-7.75 (dd, J_1 = 2.5 Hz, J_2 = 8.0 Hz, 2H), 7.71-7.69 (dd, J_1 = 2.5 Hz, J_2 = 8.5 Hz, 2H), 7.31-7.29 (d, J = 10.0 Hz, 2H), 7.04-7.02 (dd, J_1 = 3.0 Hz, J_2 = 8.5 Hz, 2H), 3.53 (s, 2H), 3.40-3.38 (m, 4H), 2.48-2.47 (d, J = 6.0 Hz, 2H). ^{13}C NMR (125 MHz, 298 K, DMSO-d_6) δ 190.61, 153.49, 134.43, 131.51, 131.07, 130.41, 127.33, 114.06, 58.50, 49.89, 43.58. HR-MS (ESI): m/z calcd for $C_{18}H_{22}BN_2O_3$ $[M+H]^+$: 325.1721; found: 325.1723.

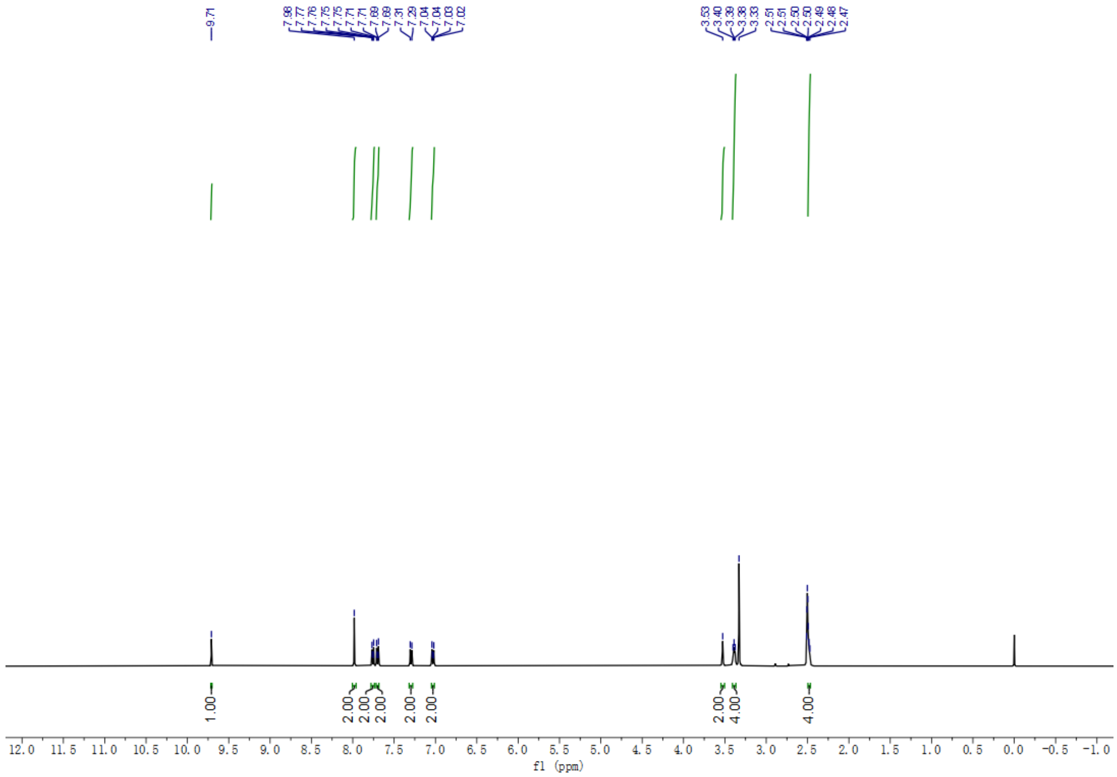

Supplementary Fig. 53 ^{1}H NMR spectrum of compound 18.

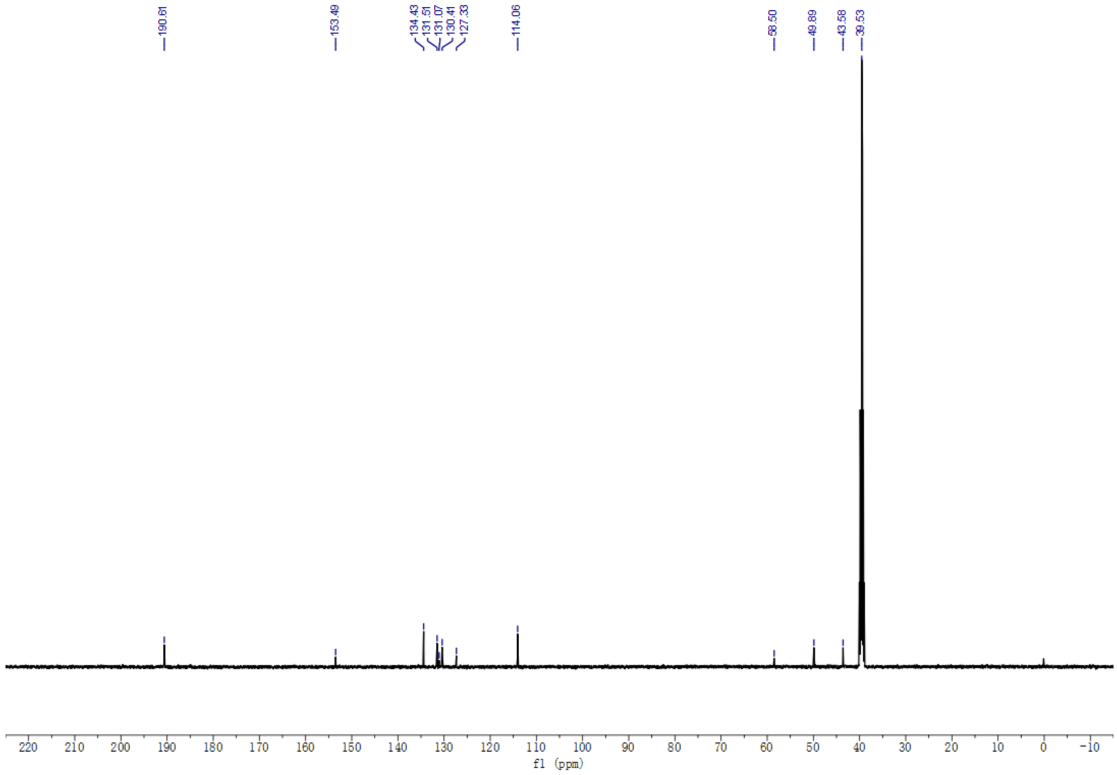

Supplementary Fig. 54 ^{13}C NMR spectrum of compound 18.

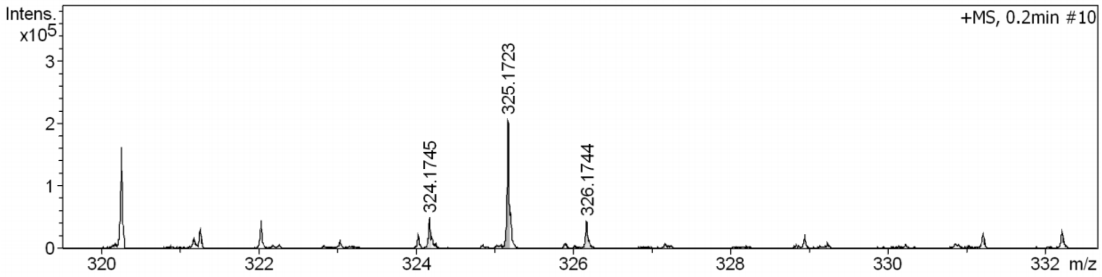

#	m/z	Res.	S/N	I	I %	FWHM
1	324.1745	13003	35.1	48192	23.5	0.0249
2	325.1723	15818	149.6	205480	100.0	0.0206
3	326.1744	13781	31.5	43220	21.0	0.0237

Meas. m/z	#	Ion Formula	m/z	err [ppm]	mSigma	#	Score	rdb	e¯ Conf	N-Rule
325.1723	1	C18H22BN2O3	325.1721	-0.4	7.0	1	100.00	9.5	even	ok

Supplementary Fig. 55 HR-ESI-MS spectrum of compound 18.

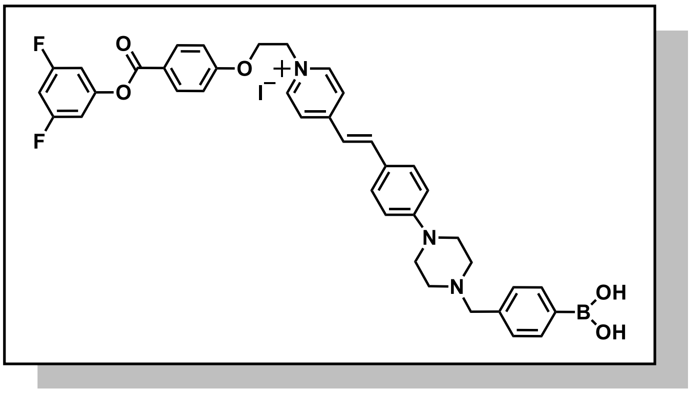

Synthesis of G1: Compound 15 (246 mg, 0.5 mmol) and Compound 18 (194 mg, 0.6 mmol) were dissolved in ethanol (20 mL). The reaction mixture was stirred and refluxed for 8 h under a nitrogen atmosphere, the resulting mixture was evaporated to dryness. After the solvent was evaporated under reduced pressure, the crude product was purified by silica gel column chromatography using DCM/MeOH(100:1, v/v) as eluent to get G1 as orange solid (160 mg, 40%).^{1}H NMR (500 MHz, 298 K, DMSO-d_6) δ 8.86-8.85 (d, J = 6.0 Hz, 2H), 8.13-8.11 (d, J = 6.0 Hz, 2H), 8.02 (s, 2H), 7.76-7.75 (d, J = 2.5 Hz), 7.61-7.60 (d, J = 7.5 Hz, 2H), 7.32-7.29 (m, 4H), 7.24 (s, 1H), 7.13-7.10 (m, 1H), 7.02-7.00 (m, 2H), 6.97-6.96 (dd, J_1 = 1.5 Hz, J_2 = 5.0 Hz, 2H), 4.89-4.87 (m, 2H), 4.54-4.52 (m, 2H), 4.03-4.01 (m, 2H), 3.53 (s, 2H), 1.99 (s, 2H), 1.60-1.57(m, 4H), 1.50-1.42 (m, 4H). ^{13}C NMR (125 MHz, 298 K, DMSO-d_6) δ 170.34, 168.59, 158.18, 154.06, 152.41, 144.30, 144.08, 141.98, 139.81, 134.10, 130.16, 130.05, 129.40, 128.68, 127.96, 124.58, 122.50, 121.48, 118.45, 114.92, 114.32, 113.32, 59.75, 52.32, 46.82, 30.95, 24.05. Maldi-TOF: m/z calcd for $C_{39}H_{37}BF_2N_3O_5$ [M]$^+$: 676.2789; found: 676.269.

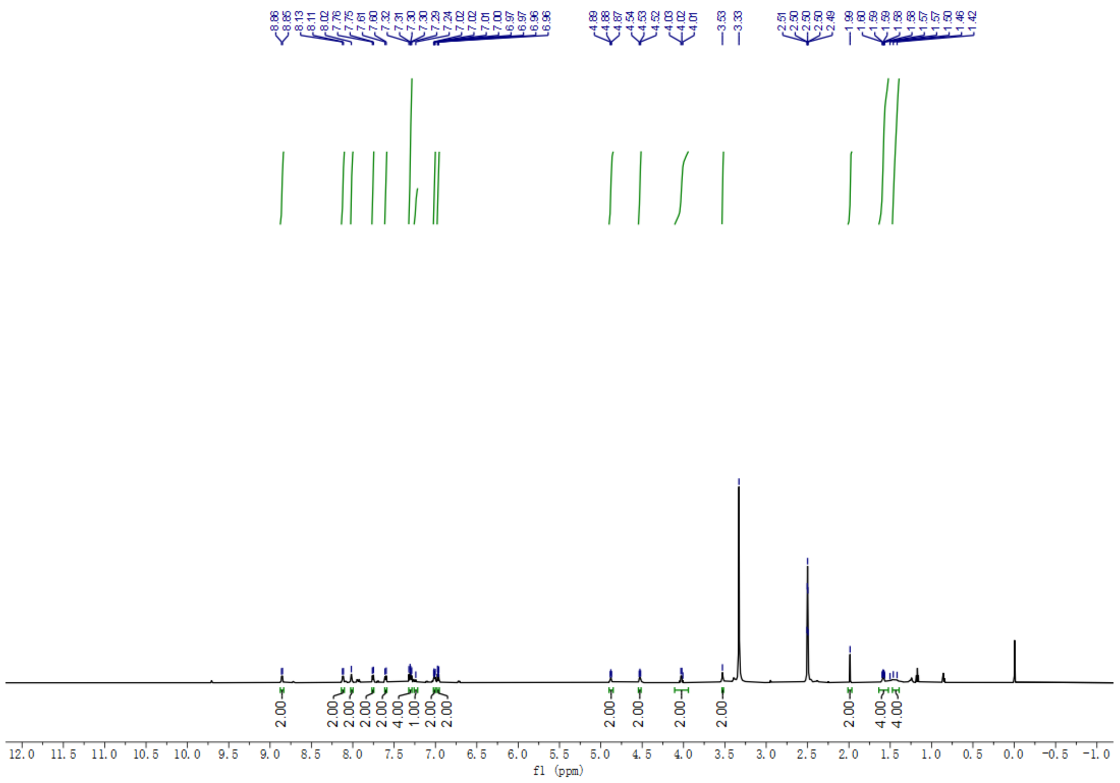

Supplementary Fig. 56 ^{1}H NMR spectrum of G1.

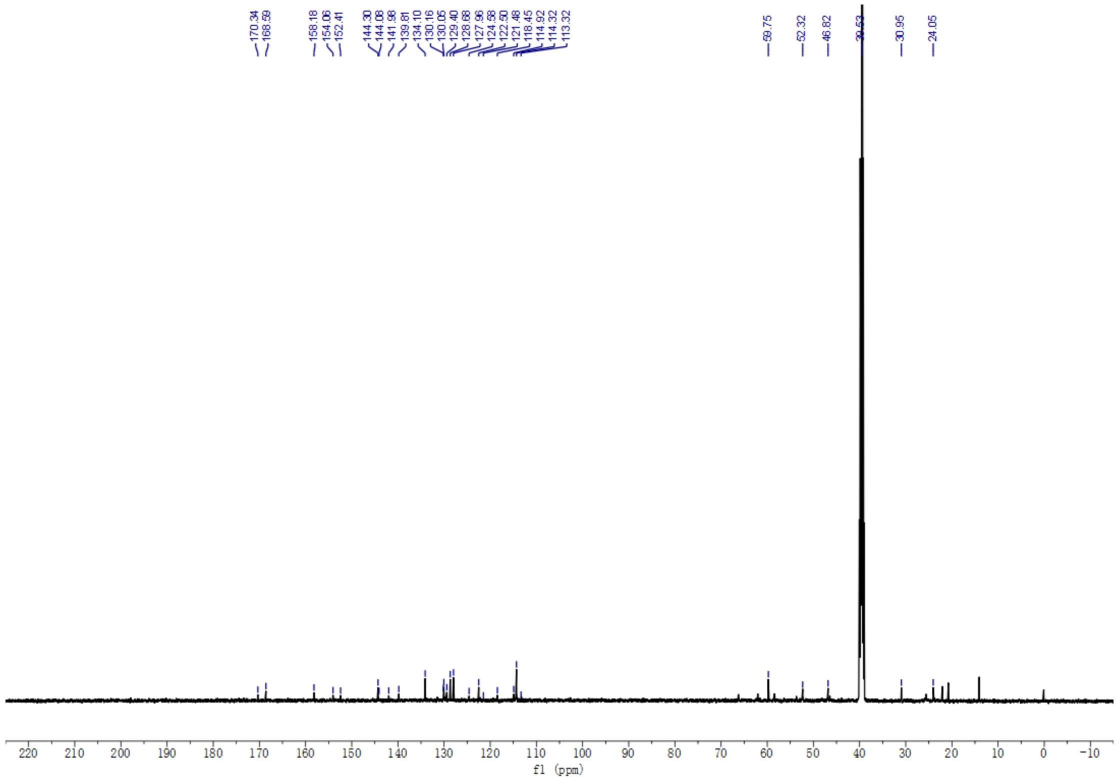

Supplementary Fig. 57 ^{13}C NMR spectrum of G1.

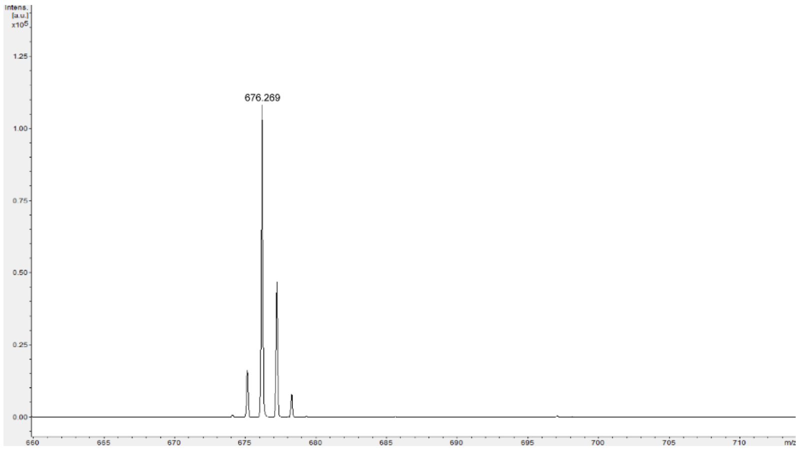

Supplementary Fig. 58 Maldi-TOF mass spectrum of G1.

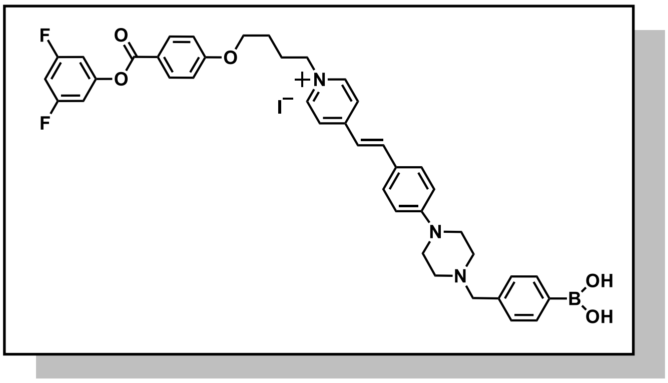

Synthesis of G2: The synthesis of G2 was the same as G1 (178 mg, 43%). ^{1}H NMR (500 MHz, 298 K, DMSO-d_6) δ 8.85-8.84 (d, J = 5.5 Hz, 2H), 8.12-8.11 (d, J = 6.0 Hz, 2H), 7.99 (s, 2H), 7.94-7.92 (d, J = 13.5 Hz, 2H), 7.76-7.75 (d, J = 6.5 Hz, 2H), 7.61-7.60 (d, J = 7.5 Hz, 2H), 7.32-7.29 (m, 4H), 7.26-7.24 (d, J = 13.0 Hz, 1H), 7.02-7.01 (d, J = 7.5 Hz, 2H), 6.96-6.95 (d, J = 7.0 Hz, 2H), 4.53-4.51 (m, 2H), 4.06-4.04 (m, 2H), 3.53 (s, 2H), 2.09-2.04 (m, 2H), 1.77-1.72 (m, 4H), 1.62-1.58 (m, 4H), 1.48 (s, 4H).^{13}C NMR (125 MHz, 298 K, DMSO-d_6) δ 168.79, 162.23, 159.15, 153.62, 152.32, 143.72, 141.61, 139.77, 134.10, 131.46, 131.20, 129.96, 128.66, 128.49, 127.96, 126.21, 124.69, 122.80, 118.55, 114.35, 114.12, 113.26, 66.90, 62.01, 58.91, 52.31, 46.85, 27.45, 24.08. Maldi-TOF: m/z calcd for $C_{41}H_{41}BF_2N_3O_5$ $[M]^+$: 704.3102; found: 704.306.

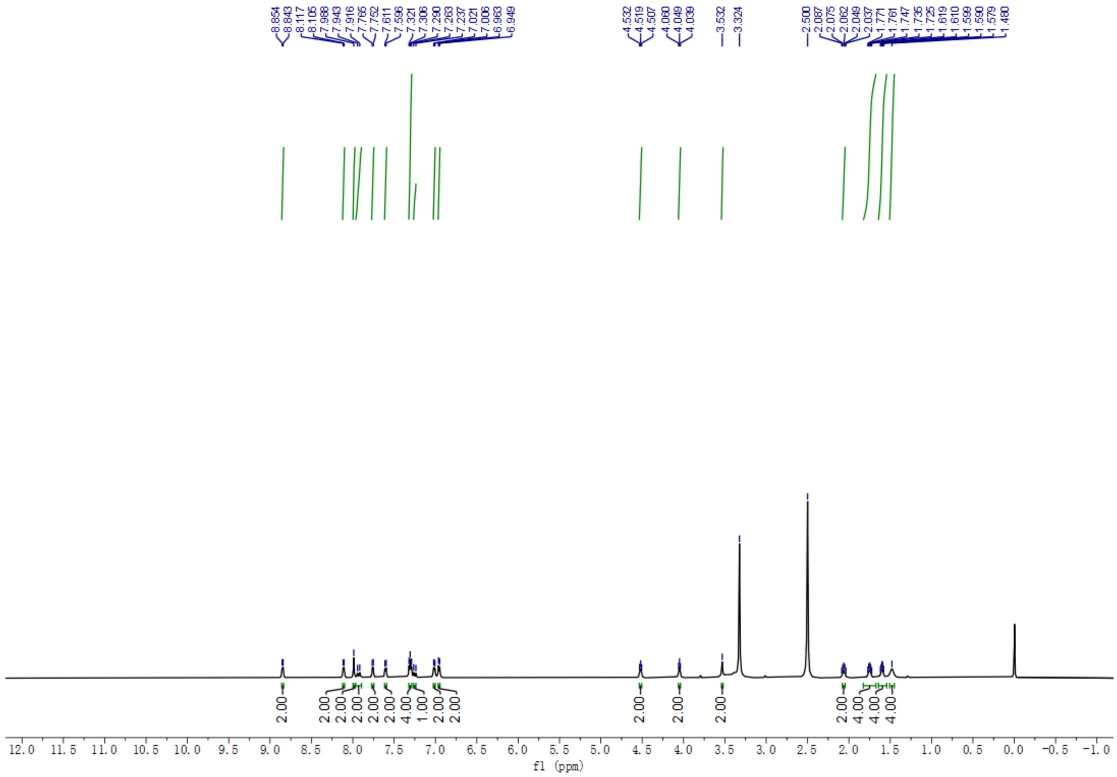

Supplementary Fig. 59 ^{1}H NMR spectrum of G2.

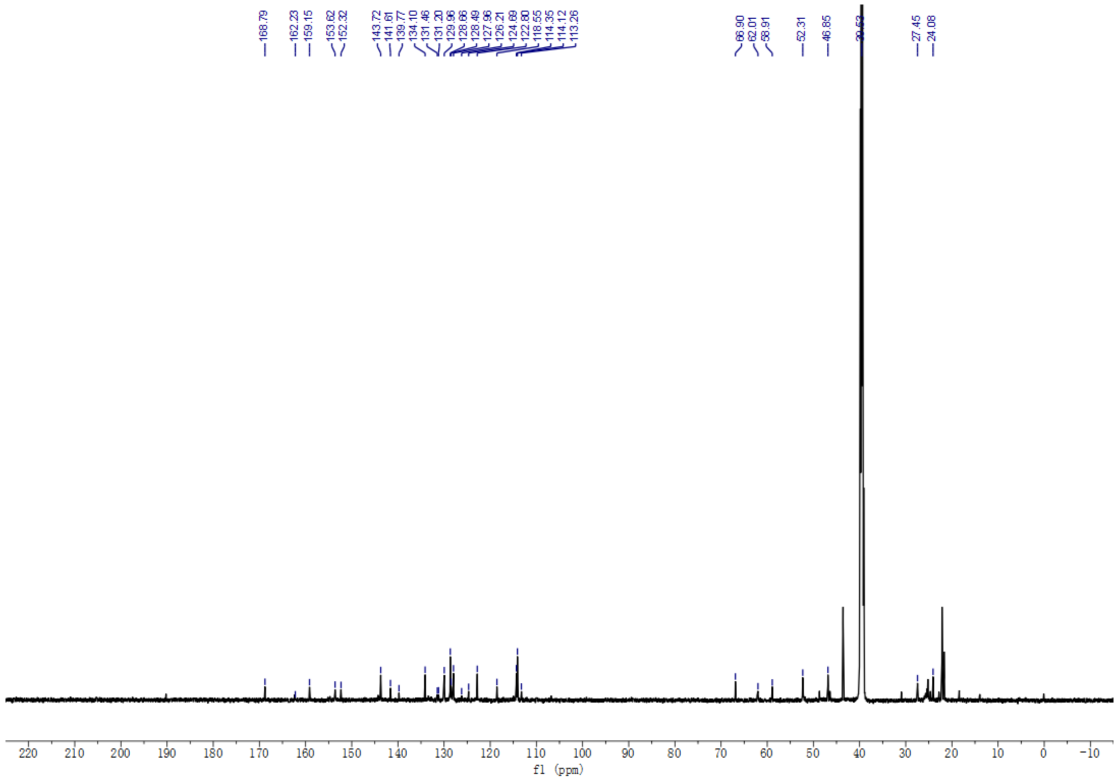

Supplementary Fig. 60 ^{13}C NMR spectrum of G2.

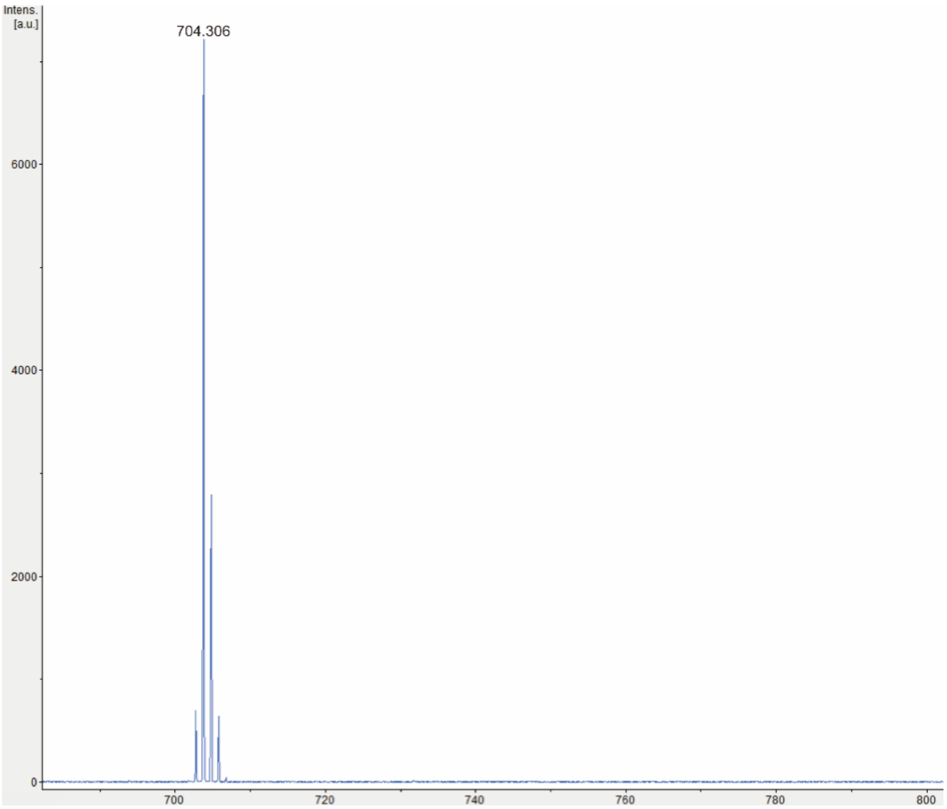

Supplementary Fig. 61 Maldi-TOF mass spectrum of G2.

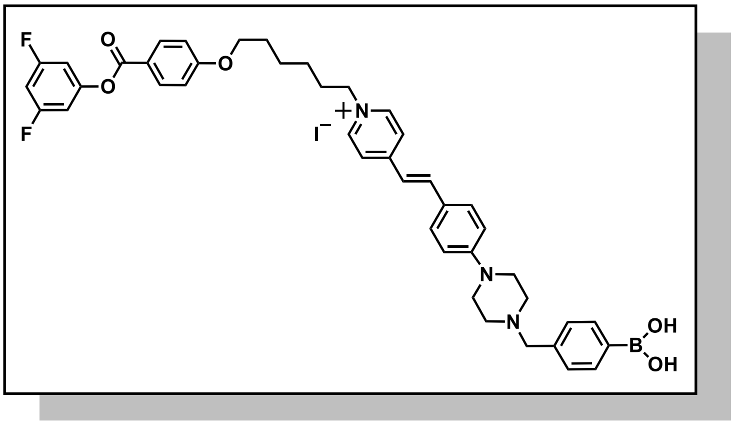

Synthesis of G3: The synthesis of G3 was the same as G1 (176 mg, 41%). ^{1}H NMR (500 MHz, 298 K, DMSO-d_6) δ 8.83-8.82 (d, *J* = 5.0 Hz, 2H), 8.10-8.09 (d, *J* = 5.5 Hz, 2H), 8.00 (s, 2H), 7.94-7.88 (m, 2H), 7.77-7.75 (d, *J* = 6.5 Hz, 2H), 7.61-7.59 (d, *J* = 7.0 Hz, 2H), 7.30-7.29 (d, *J* = 8.5 Hz, 4H), 7.26 (s, 1H), 7.02-7.00 (d, *J* = 7.5 Hz, 2H), 6.93-6.92 (d, *J* = 7.0 Hz, 2H), 4.46-4.43 (m, 2H), 3.99-3.97 (m, 2H), 3.53 (s, 2H), 1.93-1.90 (m, 2H), 1.73-1.71 (m, 2H), 1.59 (s, 2H), 1.47-1.45 (m, 4H), 1.35-1.32 (m, 4H), 1.24 (s, 2H), 0.87-0.84 (m, 2H). ^{13}C NMR (125 MHz, 298 K, DMSO-d_6) δ 168.82, 159.34, 153.56, 152.36, 143.69, 141.59, 134.11, 131.25, 131.11, 131.03, 130.16, 129.95, 128.68, 128.34, 127.96, 122.75, 118.53, 114.93, 114.35, 114.02, 113.34, 113.29, 67.37, 59.25, 52.33, 46.85, 30.96, 30.38, 28.28, 25.14, 24.09. Maldi-TOF: m/z calcd for $C_{43}H_{45}BF_2N_3O_5$ $[M]^+$: 732.3415; found: 732.342.

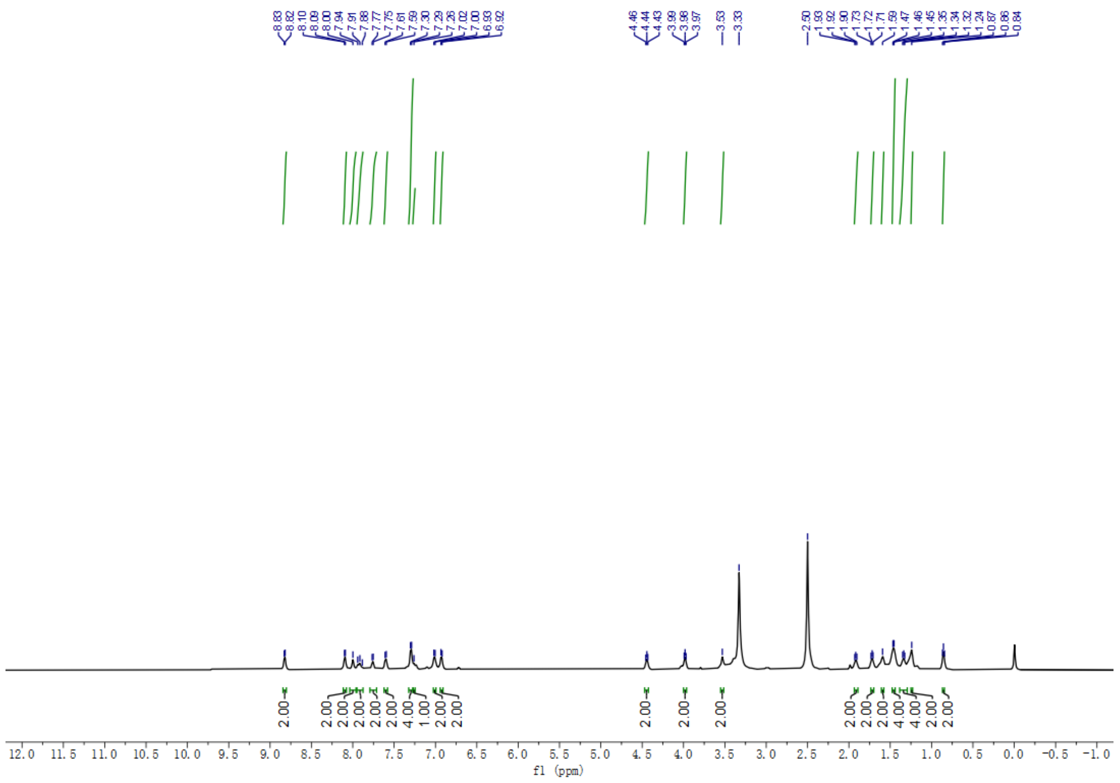

Supplementary Fig. 62 ^{1}H NMR spectrum of G3.

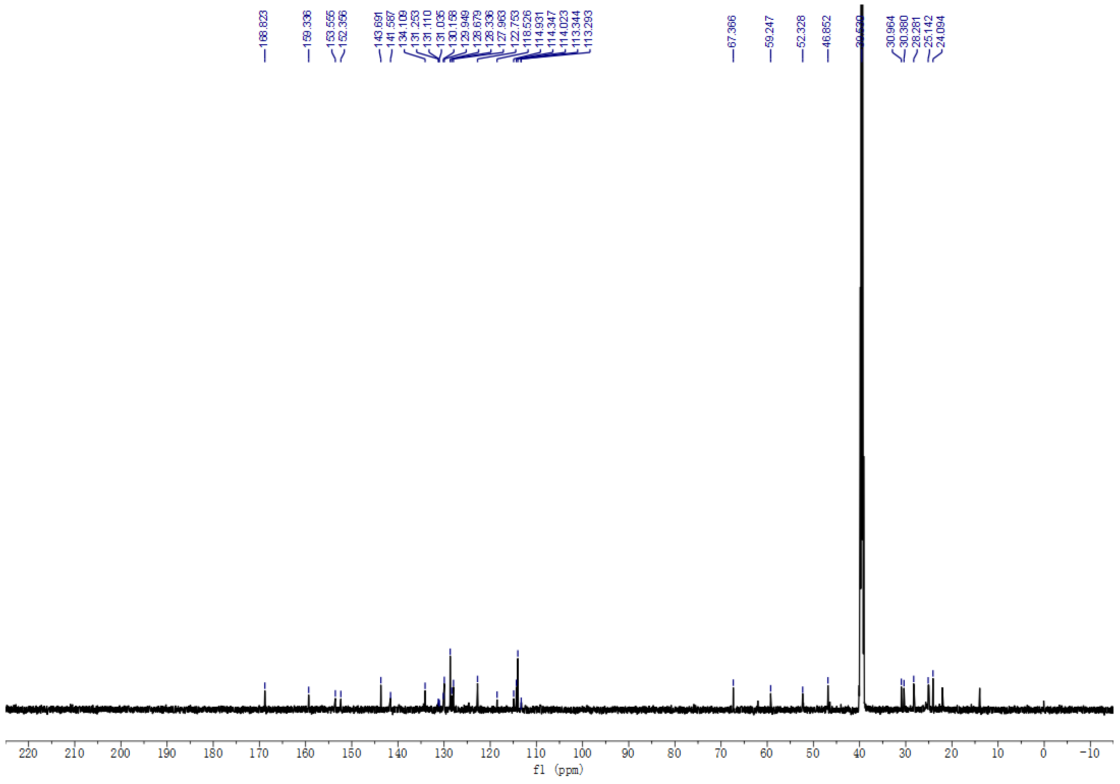

Supplementary Fig. 63 ^{13}C NMR spectrum of G3.

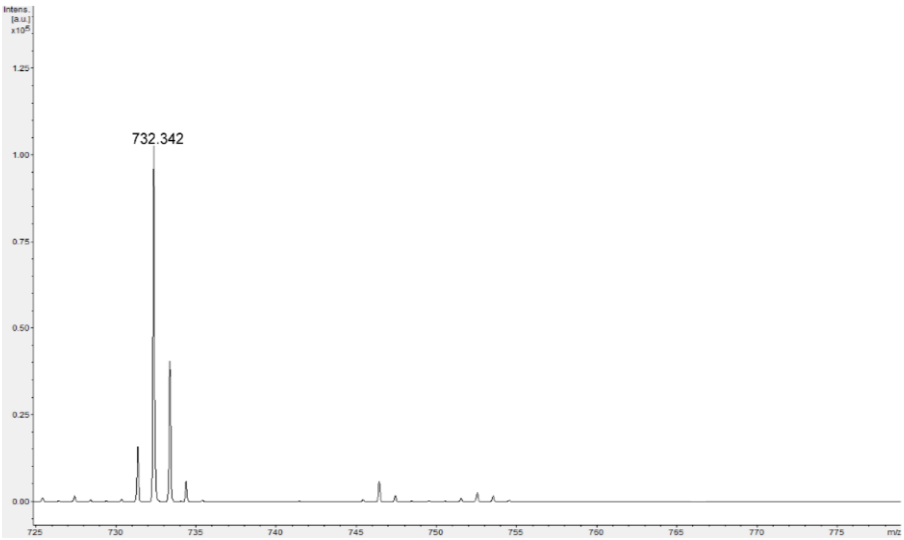

Supplementary Fig. 64 Maldi-TOF mass spectrum of G3.

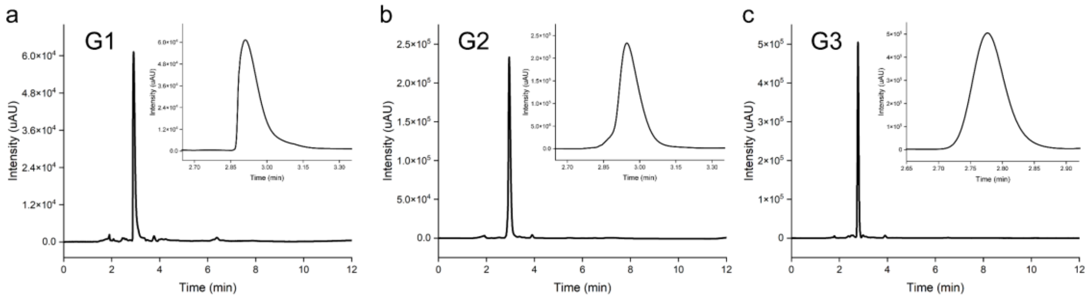

Supplementary Fig. 65 HPLC-MS spectra of (a) G1, (b) G2, (c) G3.

Supplementary Table 1 HPLC-MS results of G1, G2 and G3.

Compound	Retention time (min)	Area (nAU*min)	%Area
G1	2.9801	6516.4527	95.01
G2	2.9467	23819.1264	95.69
G3	2.7764	30550.1641	95.11

2. Optical properties of G1-G3 molecule, MB molecule assembled with different size cavities

Supplementary Table 2 Detailed dimensions of different host molecules of cucurbit[n]urils.

	CB[6]	CB[7]	CB[8]	CB[10]
Portal diameter (Å)	3.9	5.4	6.9	9.0-11.0
Cavity diameter (Å)	5.8	7.3	8.8	10.7-12.6
Cavity volume($Å^3$)	164	279	479	870
Outer diameter (Å)	14.4	16.0	17.5	18.7-21.0
Height (Å)	9.1	9.1	9.1	9.1

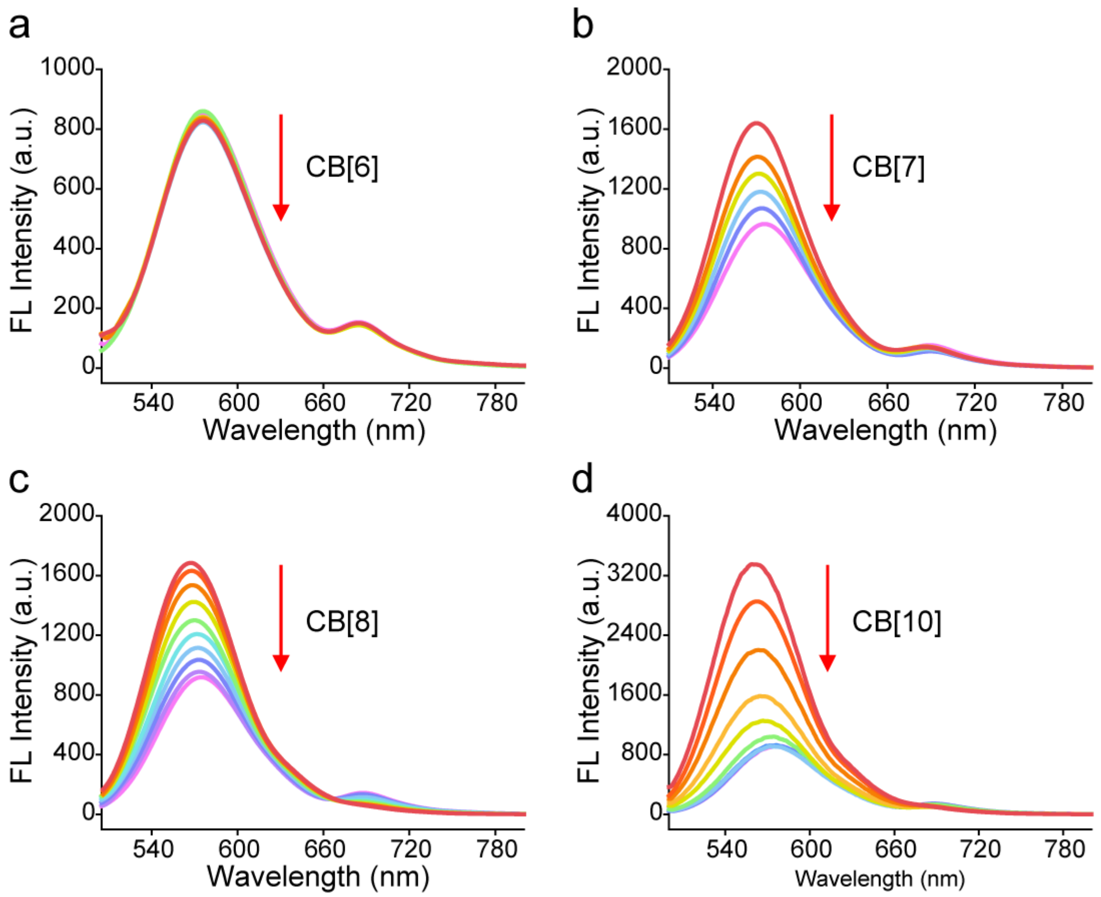

Supplementary Fig. 66 Fluorescent titration spectra of a mixture of guest molecules G2 and MB ([G2]=[MB]=10 μM) toward addition of host molecules (a-d) with different concentrations (0 - 15 μM) in PBS buffer (10 mM, pH = 7.4) containing 0.05% DMSO. (a) CB[6]; (b) CB[7]; (c) CB[8]; (d) CB[10].

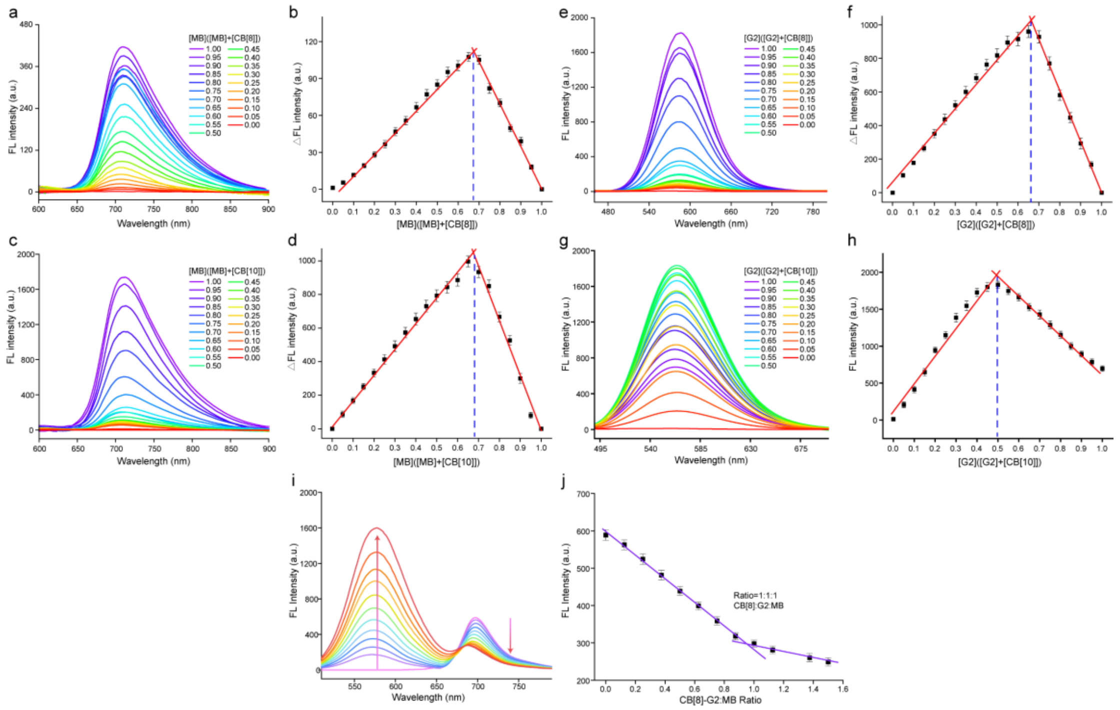

Supplementary Fig. 67 (a) Fluorescence spectra of MB+CB[8] (a), MB+CB[10] (c), G2 + CB[8] (e), and G2 + CB[10] (g) in aqueous solution with difference host : guest ratios; Job's plot for the complexation between MB and CB[8] (b) (at 695 nm), MB and CB[10] (d) (at 695 nm), G2 and CB[8] (f) (at 570 nm) and G2 and CB[10] (h) (at 570 nm) (the total concentration was fixed as 20 μM); (i) Fluorescence titration spectra of MB (10 μM) toward addition of CB[8]-G2 (concentration ratio, 1:1) with different concentration (0-15 μM); (j) CB[8]-G2 with MB assembled molar ratio; (Error bars, n = 5, S.D.).

3. Selectivity, competition tests and response speed of different probes toward determination of EP

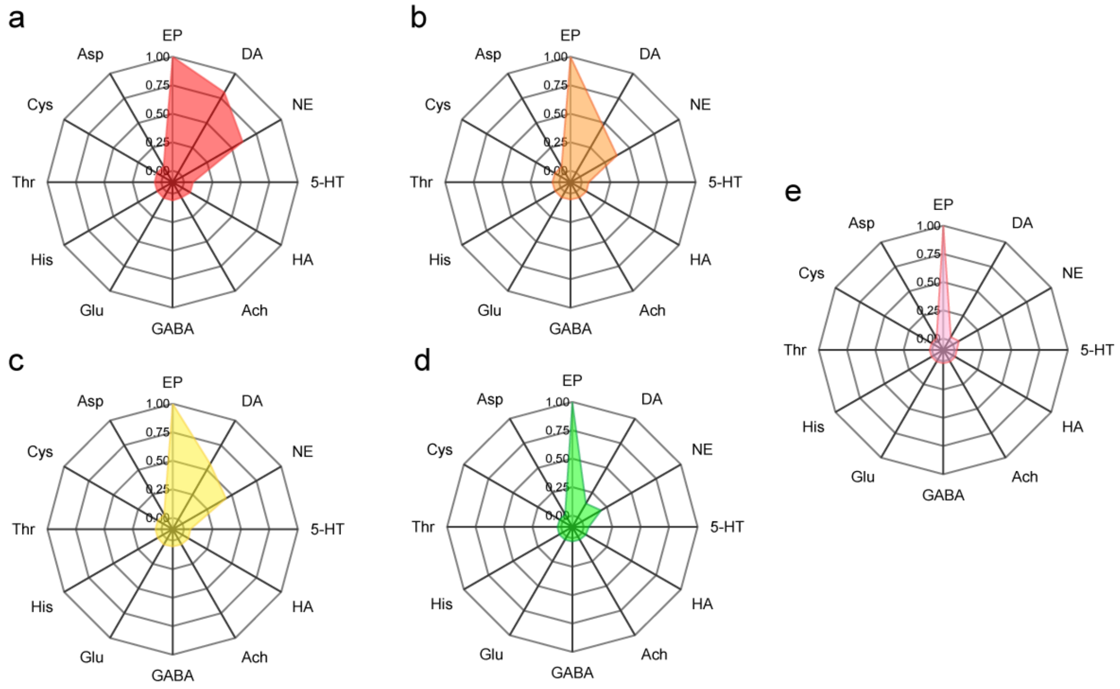

Supplementary Fig. 68 Selectivity tests for the G1-G3 and host-guest probes toward other neurotransmitters and amino acids. (a) G1; (b) G2; (c) G3; (d) CB[8]-G2-MB; (e) CB[10]-G2-MB.

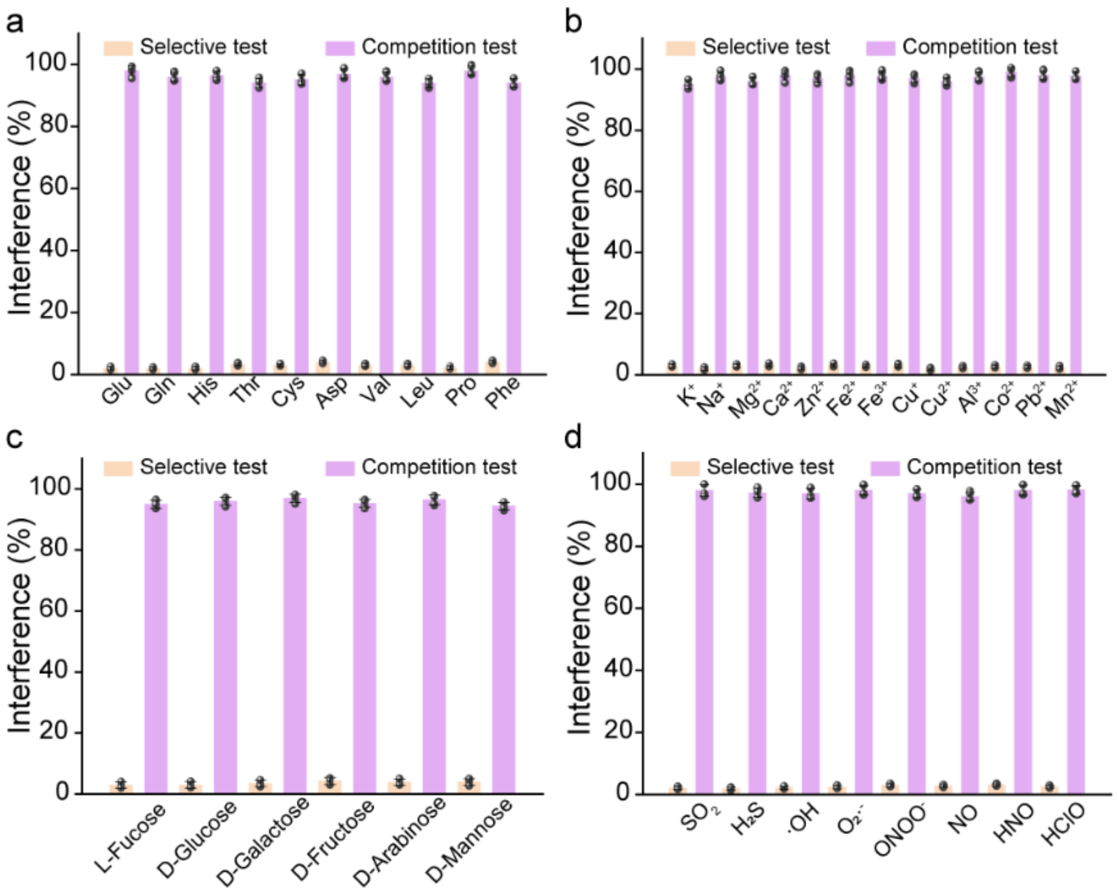

Supplementary Fig. 69 Competition tests for 10.0 μM CMG2 probe toward: (a) amino acids (1.0 mM for each); (b) metal anions (1.0 mM for each); (c) saccharides (1.0 mM for each); (d) ROS (1.0 mM for each); followed by addition of EP. Error bars, n = 5, S. D.

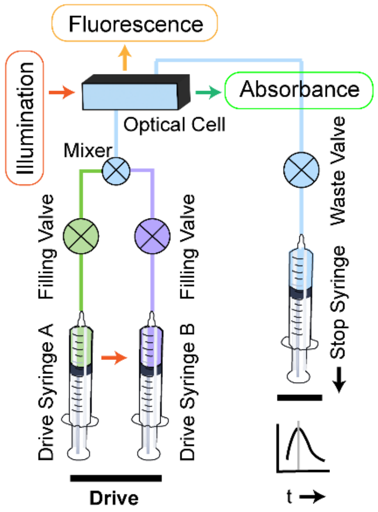

Supplementary Fig. 70 Schematic of the stopped-flow-based kinetics measurement.

4. The theoretical calculation proving high selectivity and fast response speed of CMG2

Supplementary Table 3 The energy associated in the calculation of pka of EP.

Energy	Energy,Hartree	Kcal/mol
$G^{o}_{gas}(EPH)$	-630.21	-395462.78
$G^{o}_{gas}(EP)$	-629.58	-395068.78
$\Delta G^{mod}_{solv((}(EPH)$	-0.03	-21.57
$\Delta G^{mod}_{solv}(EP)$	-0.13	-83.13
ΔG^{1M}_{aq}	---	62.15

Supplementary Table 4 The energy associated in the calculation of pka of DA.

Energy	Energy,Hartree	Kcal/mol
$G^{o}_{gas}(DAH)$	-515.85	-323697.92
$G^{o}_{gas}(DA)$	-515.22	-323307.16
$\Delta G^{mod}_{solv}(DAH)$	-0.03	-17.01
$\Delta G^{mod}_{solv}(DA)$	-0.13	-79.56
ΔG^{1M}_{aq}	---	57.92

Supplementary Table 5 The energy associated in the calculation of pka of NE.

Energy	Energy,Hartree	Kcal/mol
$G^{o}_{gas}(NEH)$	-590.99	-370852.36
$G^{o}_{gas}(NE)$	-590.37	-370460.45
$\Delta G^{mod}_{solv}(NEH)$	-0.04	-22.70
$\Delta G^{mod}_{solv}(NE)$	-0.14	-87.05
ΔG^{1M}_{aq}	---	57.28

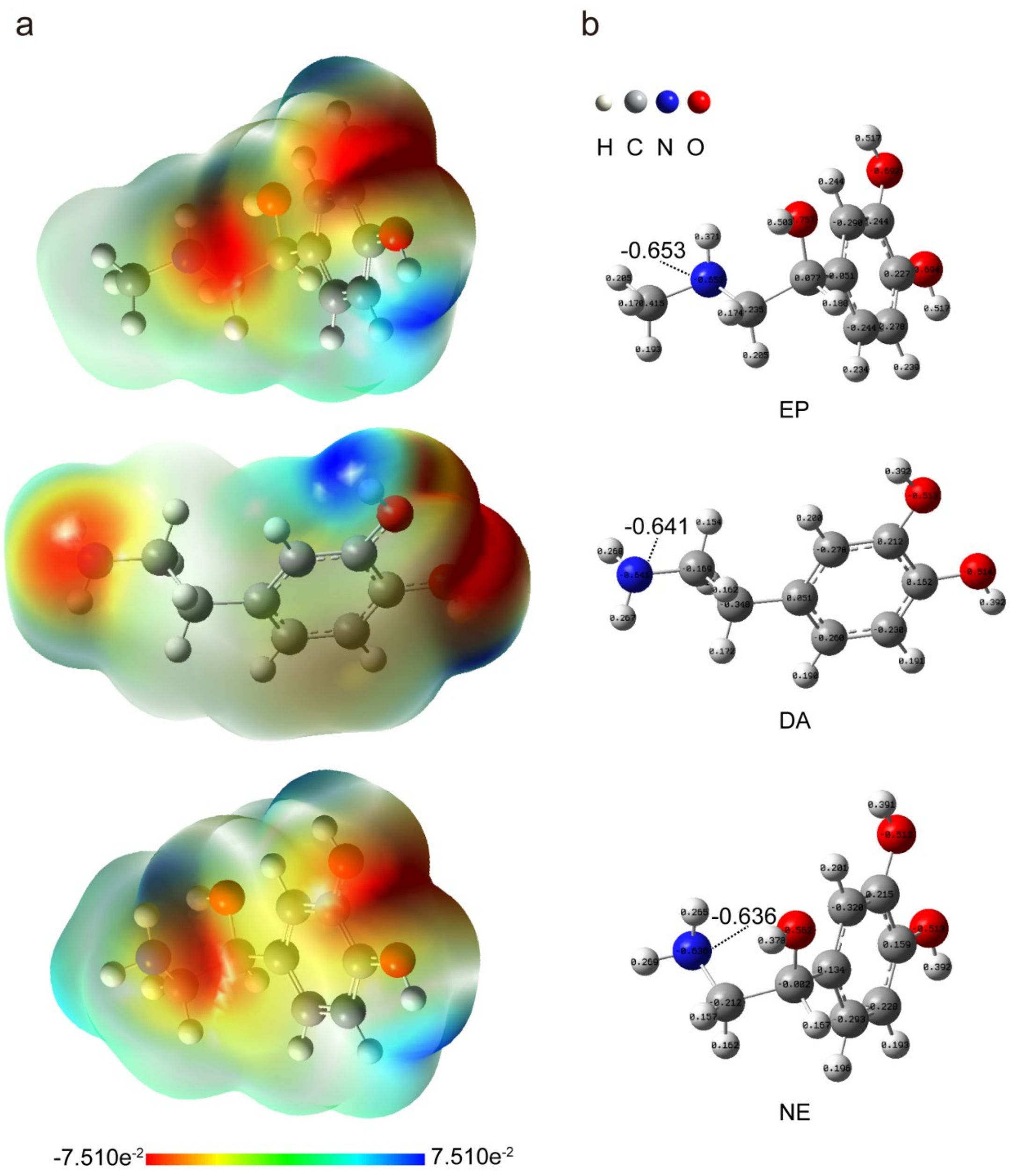

Supplementary Fig. 71 (a) The electron static potential (ESP) and (b) natural population analysis charges of EP, DA and NE.

Cartesia coordinates for EP:

Standard orientation:

Atomic	Coordinates (Angstroms)		
Type	X	Y	Z

C	-2.50332300	-0.55318100	0.08852200
C	-1.94196000	0.71381700	0.32416800
C	-0.64387200	0.98886400	-0.09850100
C	0.11759600	0.01794200	-0.75761100
C	-0.44785800	-1.23596100	-0.99545800
C	-1.74819300	-1.51772700	-0.57299600
O	-3.77704600	-0.75924500	0.52782800
O	-2.72217000	1.62363200	0.97087400
C	1.53950200	0.29501200	-1.19877300
O	1.80039800	1.68039000	-1.06524900
C	2.56715500	-0.51174700	-0.40804500
N	2.53841300	-0.17882600	1.00257000
C	3.66635800	-0.75634400	1.71065600
H	-0.23008200	1.97972800	0.09563200
H	0.12596200	-2.00402600	-1.51860100
H	-2.19672200	-2.49670500	-0.75776400
H	-4.04775700	-1.66426600	0.31576500
H	-2.22944000	2.45092200	1.07125100
H	1.63127400	-0.00791000	-2.25694100
H	2.70281400	1.83863200	-1.37553800
H	2.36544700	-1.58652800	-0.54469300
H	3.56227900	-0.32029600	-0.86553100
H	2.60029300	0.83739900	1.06579000
H	3.66286400	-0.43495700	2.76075300
H	3.59424000	-1.85420100	1.69917600
H	4.64804400	-0.48828500	1.27332400

Cartesia coordinates for DA:

Standard orientation:

Atomic	Coordinates (Angstroms)		
Type	X	Y	Z
C	2.11734000	-0.44915400	-0.12639500
C	1.53277400	0.81740500	0.06490500
C	0.17382200	0.91402700	0.34453100
C	-0.63335600	-0.22916100	0.44847500
C	-0.04249600	-1.47852400	0.25526900
C	1.32261000	-1.58582500	-0.02866000
O	3.45283700	-0.48110300	-0.39590000
O	2.35568000	1.89766500	-0.03535700
C	-2.11371000	-0.09060900	0.69587700
C	-2.89515400	0.14859500	-0.59343400
N	-4.31764200	0.30305300	-0.31470000

H	-0.25729100	1.90889700	0.49079400
H	-0.64923800	-2.38331200	0.33045400
H	1.78873500	-2.56302600	-0.17591300
H	3.73292800	-1.40103400	-0.50878200
H	1.84265900	2.70496900	0.11524700
H	-2.49529700	-1.00597400	1.17615700
H	-2.30948300	0.74712600	1.38244500
H	-2.52402000	1.07177100	-1.06535000
H	-2.66490400	-0.67447700	-1.29648700
H	-4.80991200	0.44950500	-1.19560300
H	-4.66979300	-0.58883300	0.03658800

Cartesia coordinates for NE:

Standard orientation:

Atomic	Coordinates (Angstroms)		
Type	X	Y	Z
C	-2.21770500	-0.44731300	0.05839200
C	-1.57802600	0.80406100	0.01401800
C	-0.21321100	0.87650100	-0.25003200
C	0.53855800	-0.28142200	-0.47619700
C	-0.10358100	-1.51969200	-0.43832700
C	-1.47177700	-1.59978100	-0.17049400
O	-3.55458600	-0.45376500	0.32295700
O	-2.35202500	1.90189600	0.23879800
C	2.03110000	-0.21584600	-0.72115900
O	2.38290100	1.11676100	-1.04735400
C	2.84030700	-0.69404000	0.48311600
N	2.57824800	0.13793500	1.64711900
H	0.26101800	1.85903600	-0.27920800
H	0.46383500	-2.43478900	-0.62193900
H	-1.98080000	-2.56598900	-0.14042200
H	-3.87188200	-1.36826100	0.33499800
H	-1.80110400	2.69626700	0.18413400
H	2.26663100	-0.88922000	-1.56461600
H	3.33726400	1.13790300	-1.20205500
H	2.56511400	-1.73793600	0.69647600
H	3.90387300	-0.70431400	0.17302600
H	3.23443700	-0.11131000	2.38568600
H	2.80957200	1.09912000	1.39298300

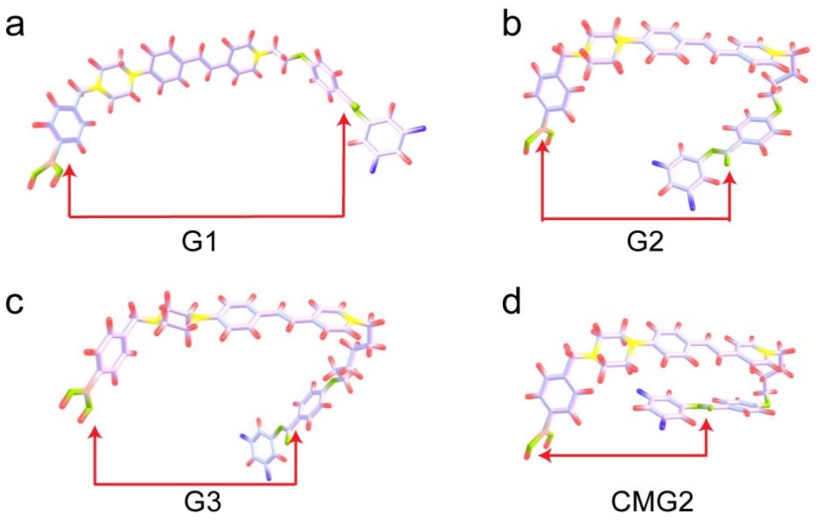

Supplementary Fig. 72 Optimal structure by DFT calculation of G1, G2 G3 and CMG2.

Supplementary Table 6 Quantification of spatial distance between two sites

	G1	G2	G3	CMG2
Distance(Å)	22.358	14.204	16.902	12.509

The calculation formula for the free energy change in the solution phase was shown in (1-3):

$$AH_{aq} = A^{-}_{aq} + H^{+}_{aq} \quad (1)$$

$$pK_a = -\log_{10}(K_a) = \frac{\Delta G_{aq}}{2.303RT} \quad (2)$$

$$\Delta G^{1\,M}_{aq} = G^{0}_{gas}(A^{-}) + G^{0}_{gas}(H^{+}) - G^{0}_{gas}(AH) + \Delta G^{1\,atm \rightarrow 1\,M} + \Delta G^{mod}_{solv}(A^{-}) + \Delta G^{mod}_{solv}(H^{+}) - \Delta G^{mod}_{solv}(AH) \quad (3)$$

5. Optical properties of CMG2 probe in response to EP

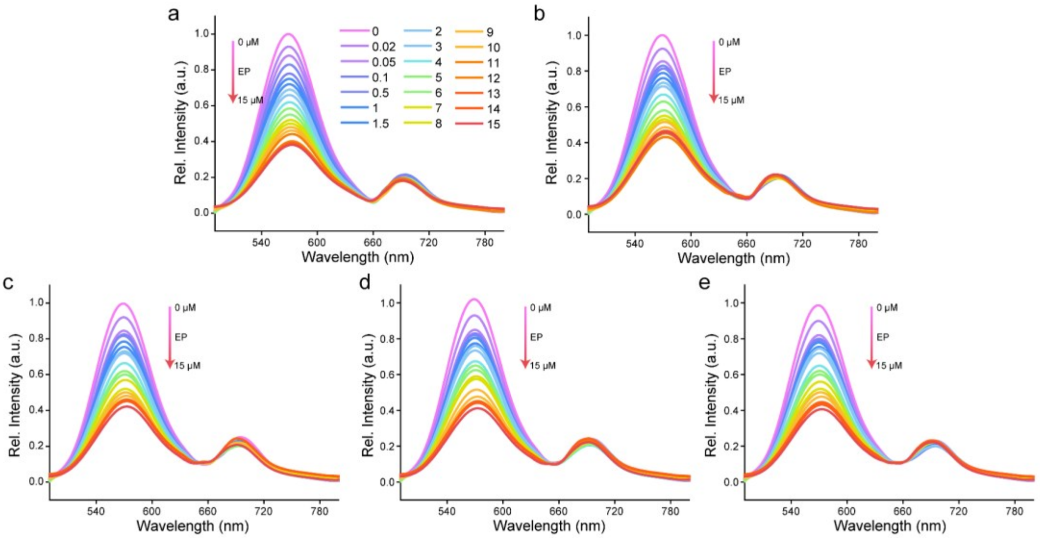

Supplementary Fig. 73 (a-e) Fluorescence spectra of 10.0 μM CMG2 with the addition of EP at different concentrations (0- 15 μM) in cell lysis buffer (10 mM, pH = 7.4) containing 0.05% DMSO excited at 480 nm.

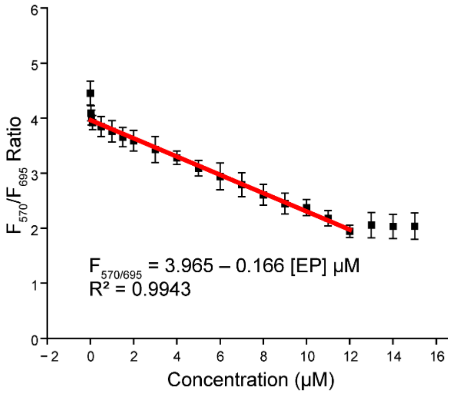

Supplementary Fig. 74 The plot of F_{570}/F_{695} ratio (F_{570}: 505 - 645 nm, F_{695}: 665 - 750 nm) of the CMG2 versus EP concentration (error bars, n = 5, S.D.).

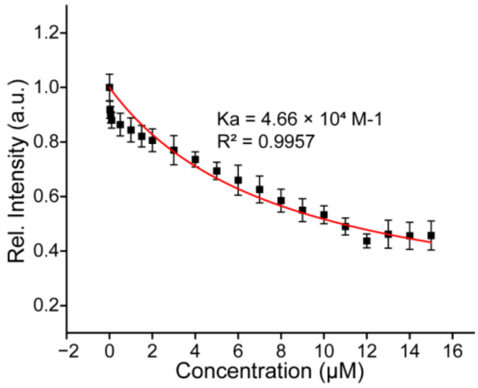

Supplementary Fig. 75 The curve-fitting for CMG2 (10.0 μM) with addition of different concentrations of EP (0 - 15 μM) and the binding affinity between CMG2 and EP. Error bars, n = 5, S. D.

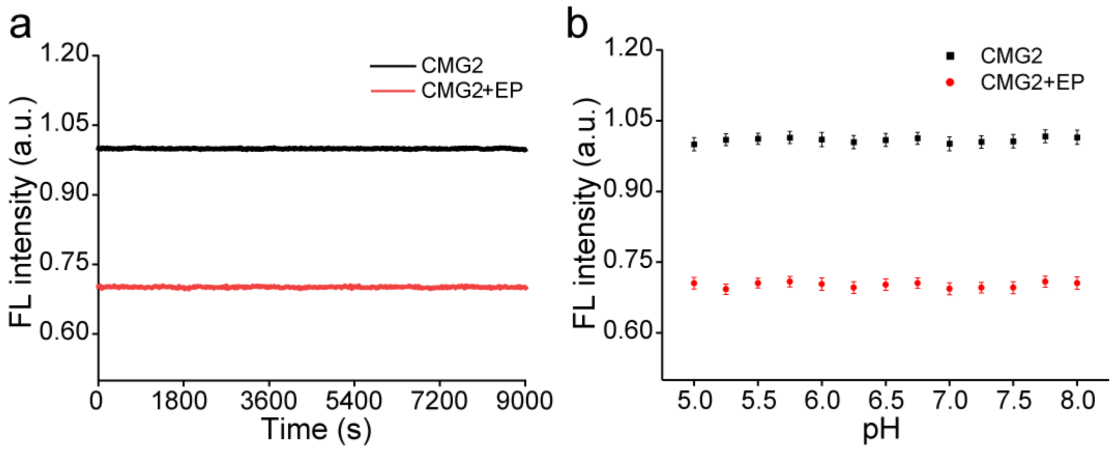

Supplementary Fig. 76 (a) Photostability test of 10.0 μM CMG2 probe before (black line) and after (red line) addition of EP (5.0 μM); (b) The pH stabilities of 10.0 μM CMG2 probe before (black dots) and after (red dots) addition of EP (5.0 μM) with pH changing from 6.0 to 10.0.

6. Sensing mechanism of Host-guest self-assembly of CMG2 and CMG2+EP

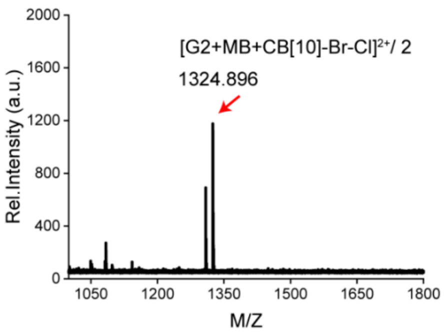

Supplementary Fig. 77 Maldi-TOF mass spectrometry of CMG2.

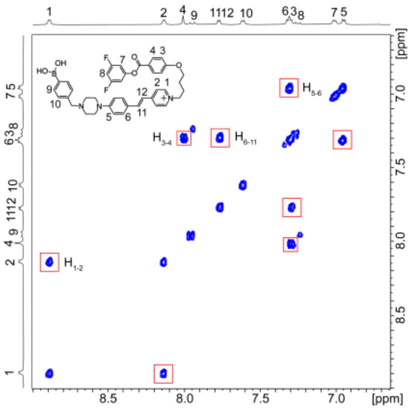

Supplementary Fig. 78 2D COSY spectrum of the guest molecule G2.

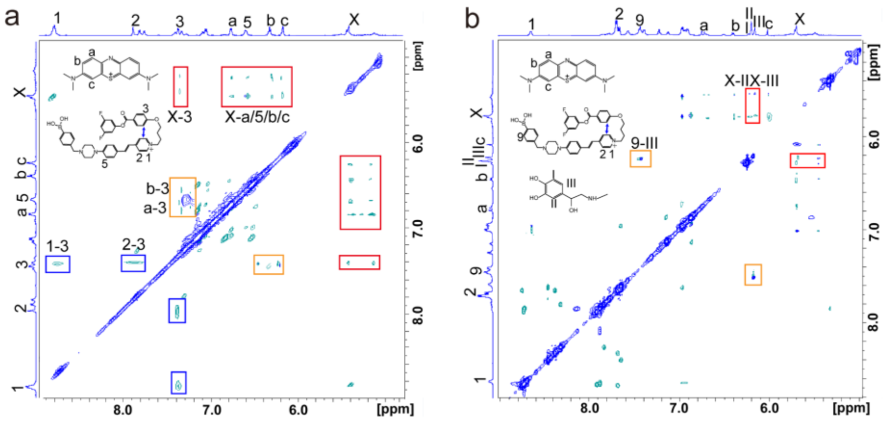

Supplementary Fig. 79 (a) ROESY spectrum of host-guest interaction between CB[10], G2 and MB; (b) ROESY spectrum of host-guest interaction between CMG2 and EP.

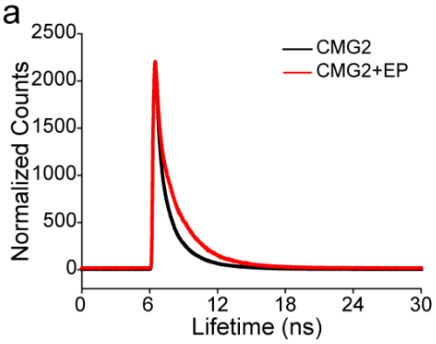

b

τ	CMG2	CMG2+EP
$\tau_{average}$	1.783 ns	2.486 ns
τ_1	2.285 ns	3.538 ns
τ_2	0.616 ns	1.612 ns

Supplementary Fig. 80 (a) Fluorescence lifetime test of 10.0 μM CMG2 probe before (black dots) and after (red dots) addition of EP (5.0 μM); (b) Table of fluorescence lifetime of CMG2 and CMG2+EP.

7. FACS, MTT and photostability measurements of CMG2 probe in live cells

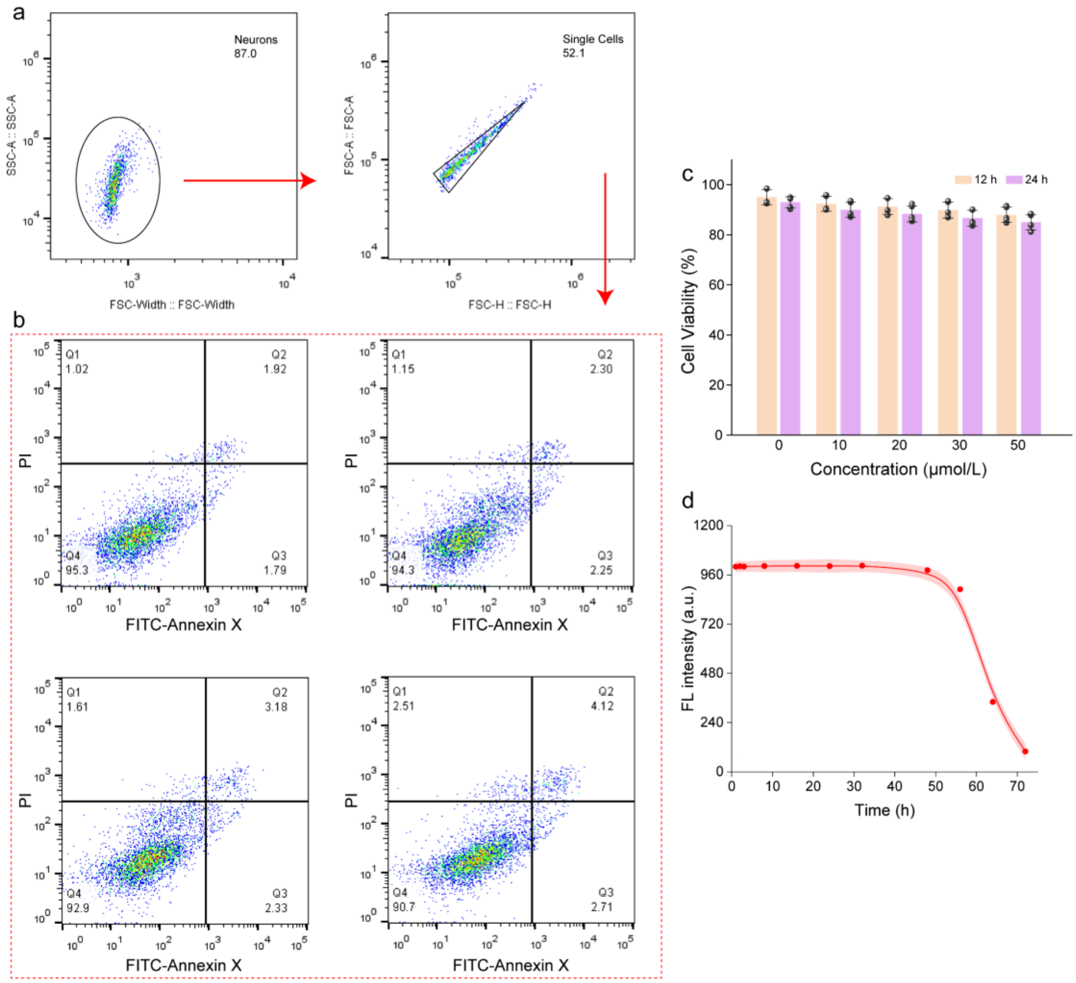

Supplementary Fig. 81 (a) Representative FACS gating strategy of neurons sorting; (b) The apoptosis assay of neurons incubated with CMG2 probe using different concentrations of 0 μM, 10 μM, 30 μM and 50 μM for 24 h. Q1, Q2, Q3, and Q4 represent the regions of dead neurons, late apoptotic neurons, early apoptotic neurons, and normal neurons, respectively; (c) The MTT assay for neurons upon incubation of CMG2 probe with different concentrations (0, 10, 20, 30 and 50 μM) after 12 h and 24 h, respectively; (d) Photostability test of CMG2 probe in neurons. Error bars, n = 5, S. D.

8. Fluorescence imaging and simultaneous quantification EP in vivo

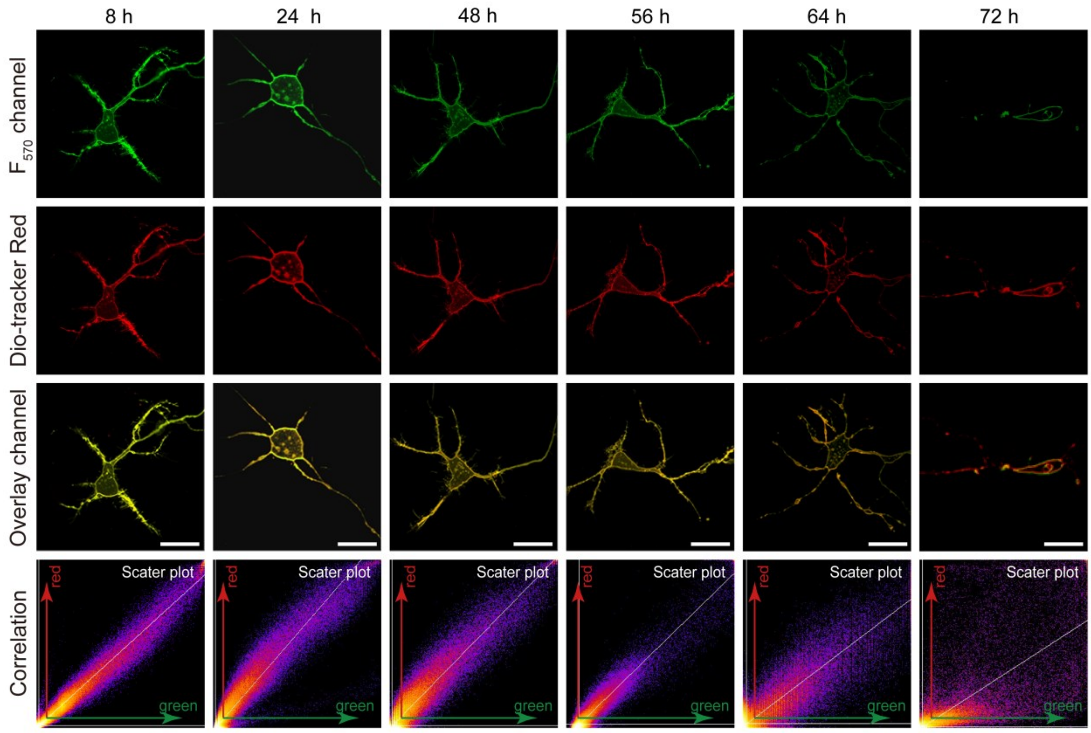

Supplementary Fig. 82 Confocal fluorescence images of neurons treated with CMG2 and Dio-tracker Red for different times (8, 24, 48, 56, 64, 72 h). Scale bar: 15 μm.

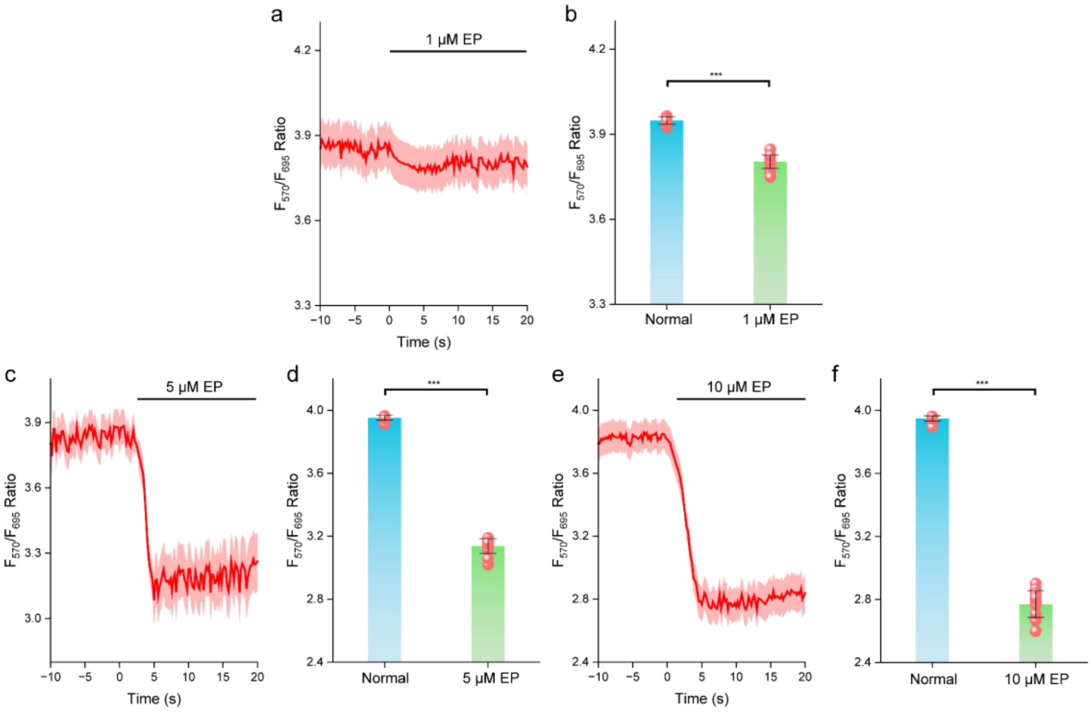

Supplementary Fig. 83 Representative traces of CMG2 in response to (a)1 μM EP; (c) 5 μM EP; (e) 10 μM EP; Dynamic response summary of CMG2 in response to (b) 1 μM EP (EP from 0.09 ± 0.02 μM to 0.99 ± 0.04 μM); (d) 5 μM EP (EP from 0.09 ± 0.02 μM to 4.97 ± 0.07 μM); (f) 10 μM EP (EP from 0.09 ± 0.03 μM to 7.20 ± 0.13 μM). The fluorescence intensity error was represented by the standard deviation (SD), while the concentration error was calculated using the standard error of the mean (SEM). Red dots represent individual data points. Statistical significance was calculated with a two-tailed unpaired t-test (n = 15; ***$p < 0.001$).

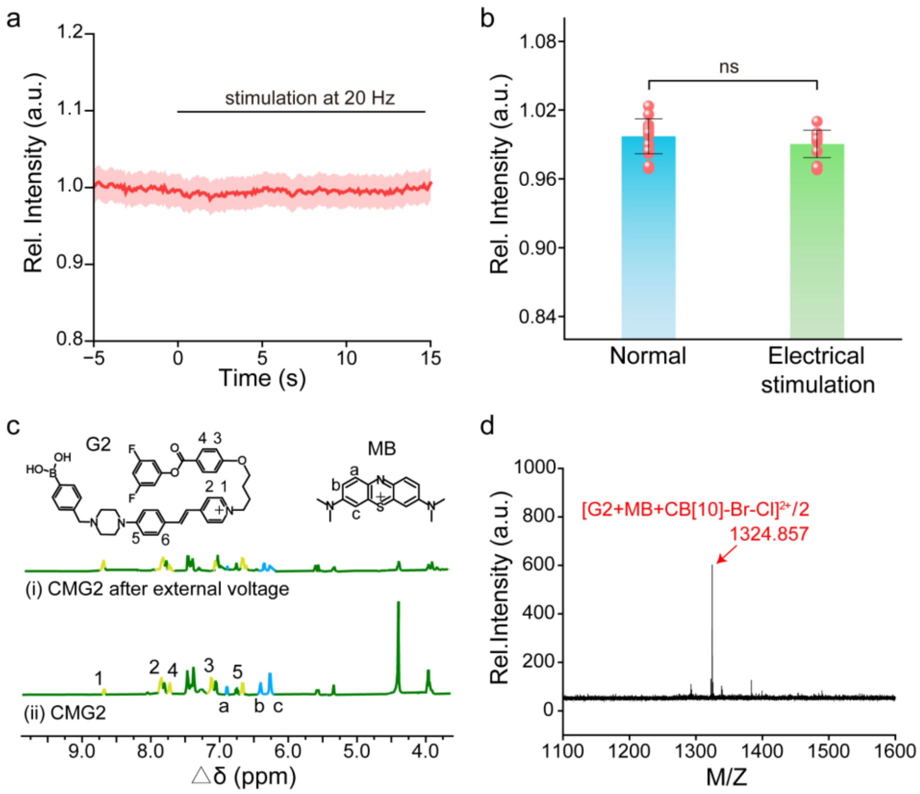

Supplementary Fig. 84 (a) Fluorescent intensity representative traces of CMG2 in solution after electrical stimulation; (b) Dynamic response summary of CMG2 in solution after electrical stimulation. The data is presented as mean ± S.D. Error bars: S.D., red dots represent individual data points. Statistical significance is calculated with a two-tailed unpaired t-test (n = 15; $^{ns}p > 0.05$); (c) ^{1}H NMR spectra of CMG2 after electrical stimulation (20 Hz, 3 V, sine wave for 1.0 s) (i) and CMG2 (ii); (d) Maldi-TOF mass spectrometry of CMG2 after external voltage.

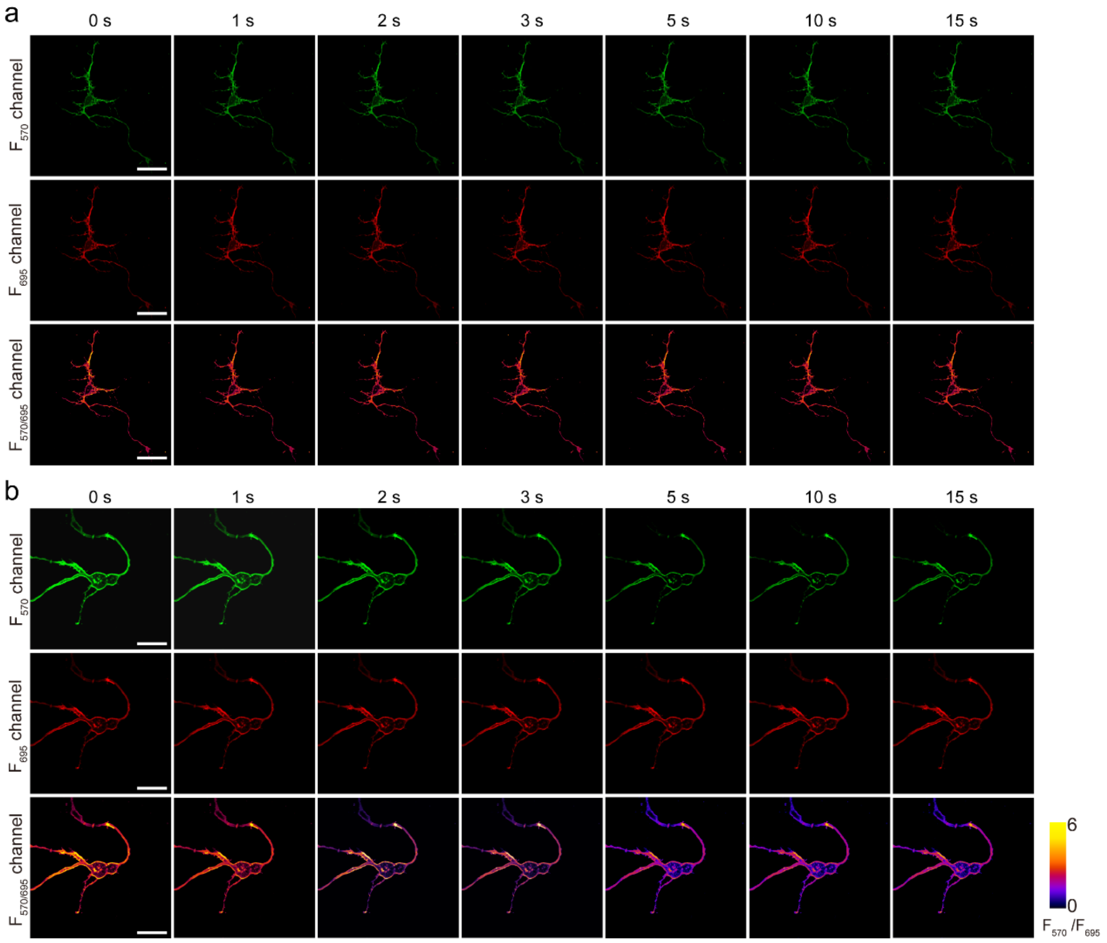

Supplementary Fig. 85 Real-time fluorescence imaging of neurons incubated with CMG2 sensor stimulated by PBS buffer (a) or electrical stimulation (b), respectively. Scale bar: 15 μm.

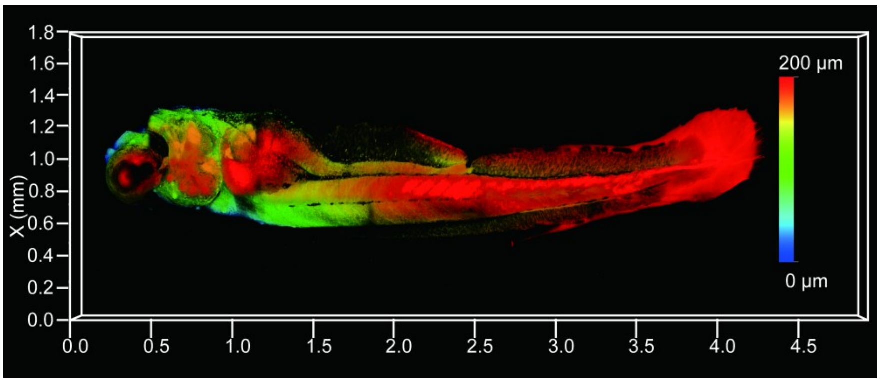

Supplementary Fig. 86 3D imaging of EP in living larval zebrafish.

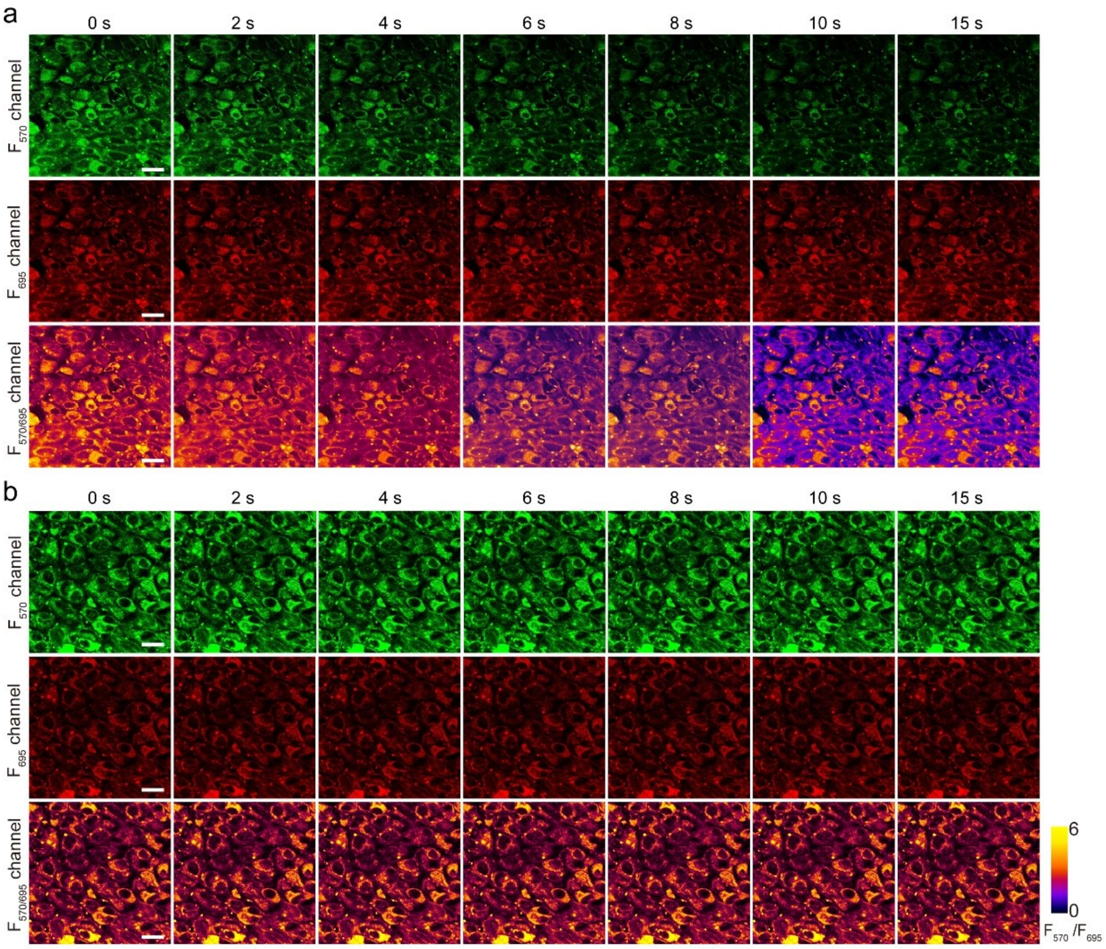

Supplementary Fig. 87 Real-time fluorescence imaging of zebrafish incubated with CMG2 sensor stimulated by electrical stimulation (a) or PBS buffer (b), respectively. Scale bar: 10 μm.

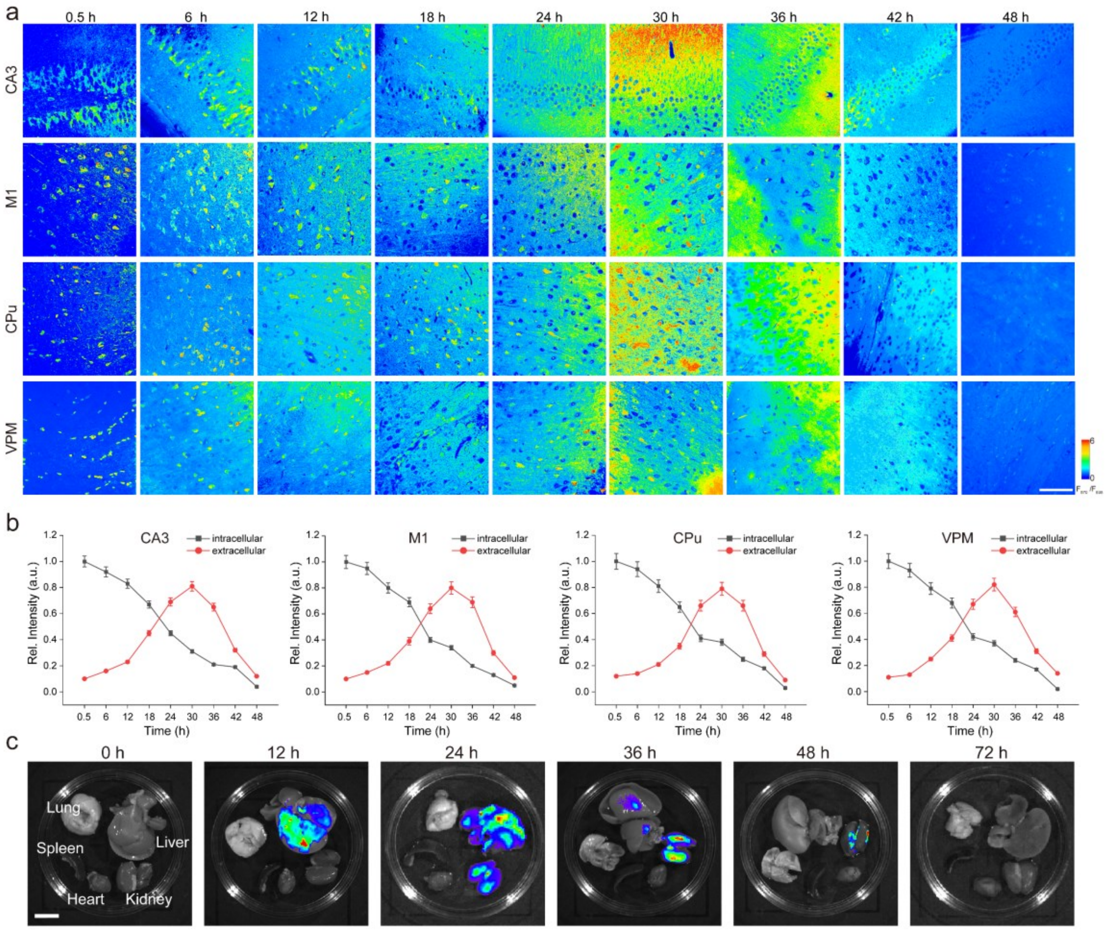

Supplementary Fig. 88 (a) Fluorescence images of brain tissue slices from different brain regions over time; Scale bar: 85 μm. (b) Statistical quantification of intracellular and extracellular fluorescence intensity; (c) Fluorescence imaging of different organs obtained from the live mice after CMG2 probe was injected into the brain for different times (0 h, 12 h, 24 h, 36 h, 48 h, and 72 h), Scale bar: 1 cm.

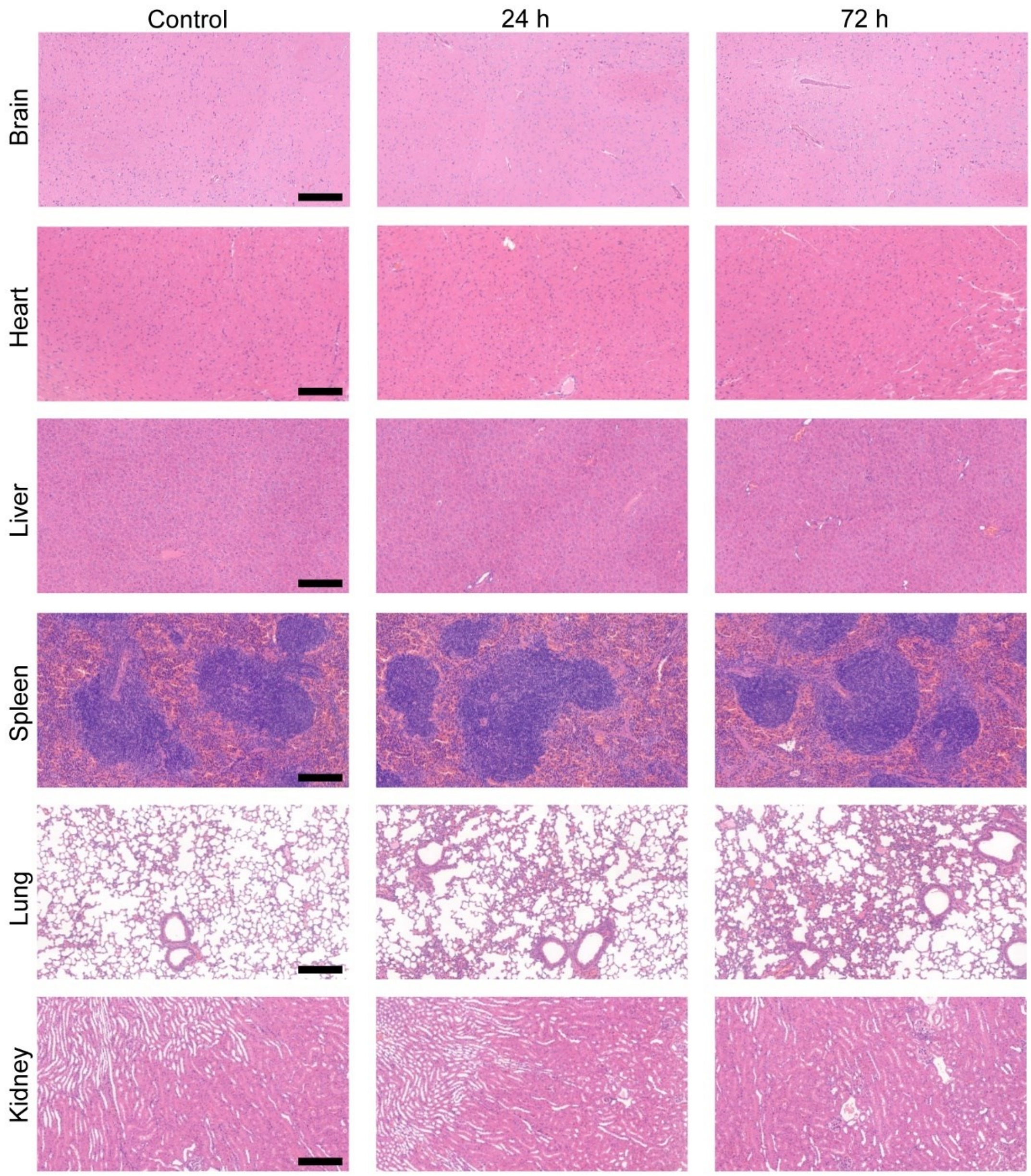

Supplementary Fig. 89 HE staining of brain and different organs (heart, liver, spleen, lung and kidney) tissues after CMG2 probe was injected into mice brain for different times (0 h, 24 h, and 72 h). Scale bar: 200 μm.

9. The optical properties characterization, co-localization biocompatibility of the optical fiber array

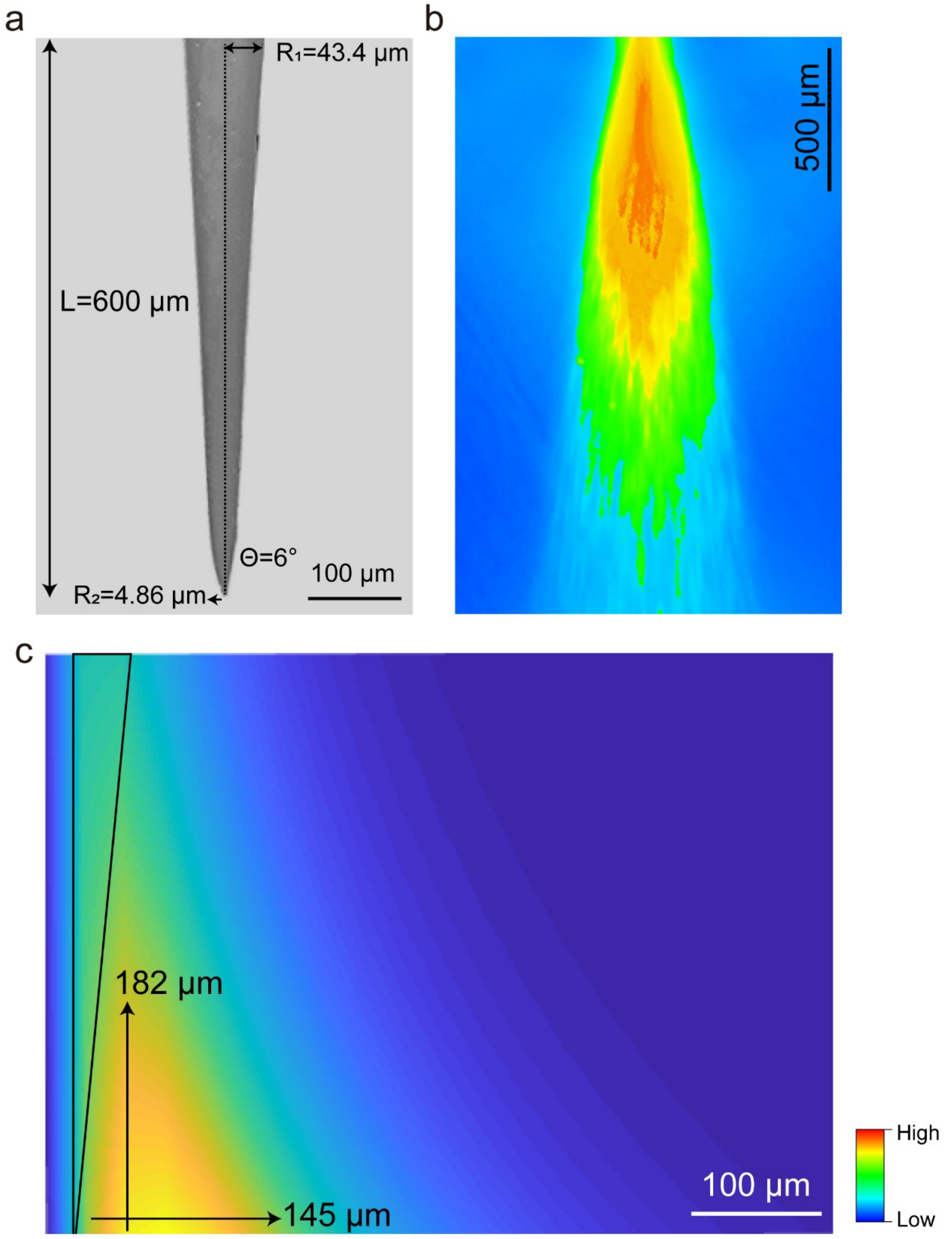

Supplementary Fig. 90 (a) SEM images of the tapered fiber; Scale bar: 100 μm. (b) Light delivery geometry of the tapered fiber in rhodamine solution; Scale bar: 500 μm. (c) Simulation of optical field distribution. Scale bar: 100 μm.

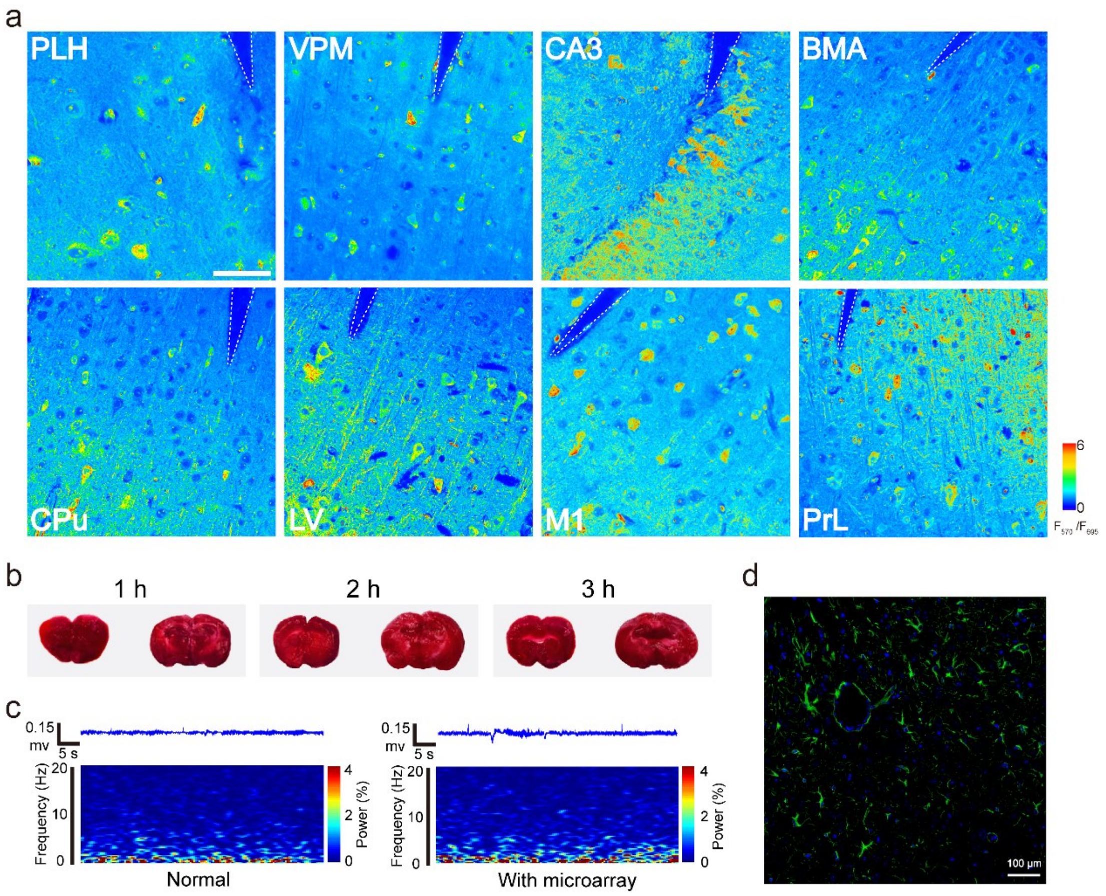

Supplementary Fig. 91 (a) Confocal images of brain tissues in different regions after implantation of multi-fiber microarray; Scale bar: 75 μm. (b) TTC staining of brain tissues obtained from mouse brains after the fiber microarray was implanted into the live brain for different times (1 h, 2 h, and 3 h); (c) LFP signals (left) and power spectra (right) in hippocampus of mice brain in the absence and presence of fiber microarray; (d) Confocal images of astrocytes stained for GFAP (green) surrounding an optical fiber shaft in brain sections cut from the live mouse brain. Scale bar: 100 μm.

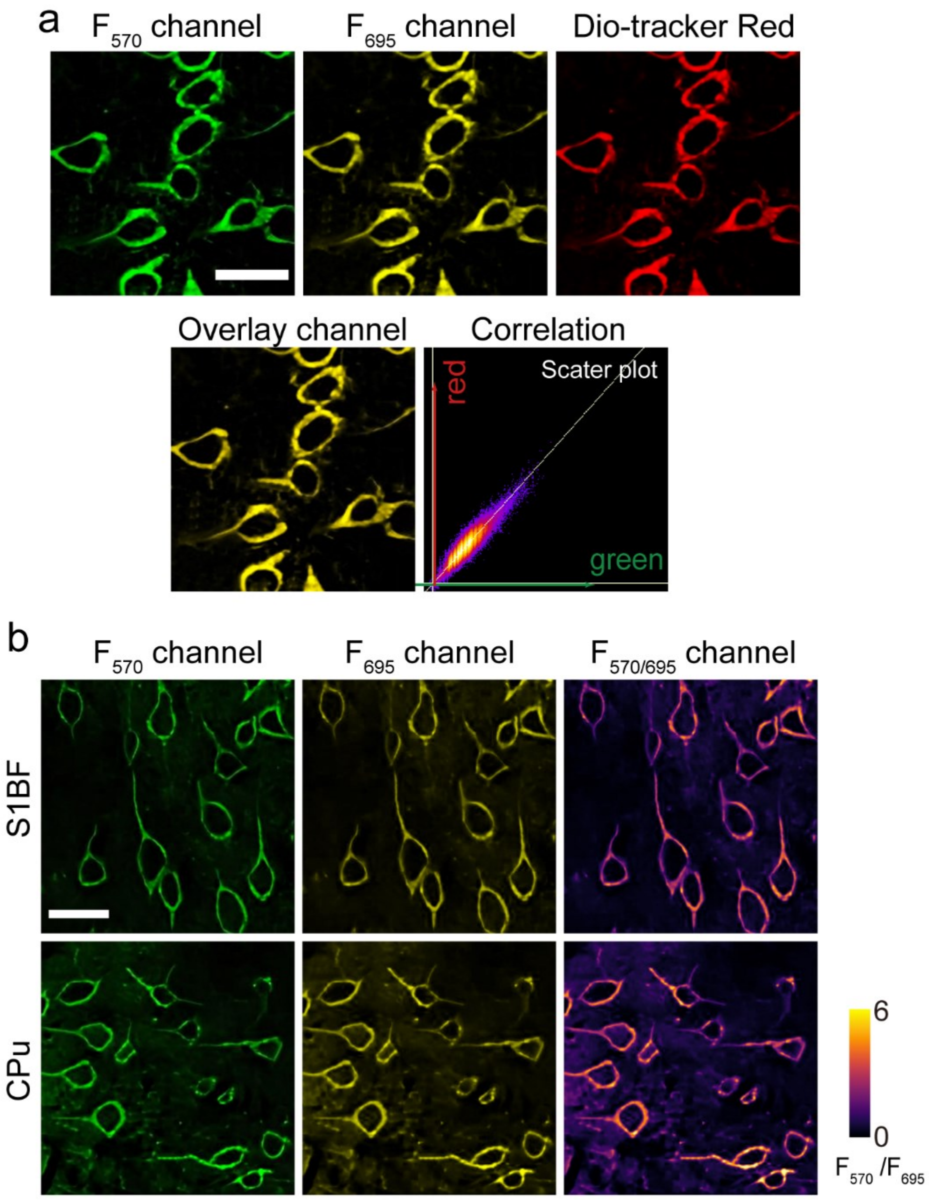

Supplementary Fig. 92 (a) Confocal fluorescence images of brain tissue slices in S1BF costained with CMG2 and a commercial membrane probe (Dio); Scale bar: 50 μm. (b) Fluorescence images of brain tissue slices from different subregions; Scale bar: 50 μm.
